# *De novo* biosynthesis of eriocitrin in *Saccharomyces cerevisiae* through deep learning-guided enzyme screening and systematic metabolic engineering

**DOI:** 10.1016/j.synbio.2026.01.019

**Published:** 2026-02-04

**Authors:** Qiyuan Lu, Xinjia Tan, Siqi Zhang, Yongtong Wang, Yifei Zhao, Juan Liu, Fanglin Hu, Shasha Zuo, Jiaxu Chen, Liusha Fan, Shenghua Ding, Zhiqiang Xiao, Yang Shan

**Affiliations:** a Longping Agricultural College, Hunan University, Changsha, 410125, China; b DongTing Laboratory, Hunan Institute of Agricultural Product Processing and Quality Safety, Hunan Academy of Agricultural Sciences, Changsha, 410125, China

**Keywords:** Deep learning, Flavonoid-7-*O*-Glucosyltransferase, UDP-Glucose, Eriodictyol, Eriocitrin

## Abstract

Eriocitrin, a flavanone-7-*O*-disaccharide known for its antioxidant and anti-inflammatory properties, holds considerable promise for use in functional foods and pharmaceuticals. However, its large-scale production is constrained by the limitations of conventional plant extraction, including low abundance of active compounds and seasonal variability. Here, we established a *de novo* biosynthetic pathway for eriocitrin from glucose in engineered *Saccharomyces cerevisiae* through combinatorial metabolic engineering. First, to verify the feasibility of glycosylation in the eriocitrin synthesis process, two highly efficient flavonoid-7-*O*-glucosyltransferases were identified using an integrated bioinformatics and deep learning approach. Subsequently, through the reconstruction of the UDP-rhamnose synthesis pathway and enhancement of the key glycosylation precursor UDP-glucose supply, an eriocitrin titer of 147.9 mg/L was achieved following supplementation with 300 mg/L eriodictyol. To further achieve the *de novo* synthesis of eriocitrin, an eriodictyol-producing strain was developed by screening the optimal F3′H/CPR pair, engineering promoters, and pathway integration, achieving an eriodictyol titer of 237.8 mg/L. Finally, combining the eriodictyol-producing chassis strain with the previously optimized glycosylation module enabled the *de novo* production of eriocitrin, reaching a titer of 30.5 mg/L. This work demonstrates a proof-of-concept for microbial production of flavonoid disaccharides, providing a foundation for a sustainable and scalable supply of eriocitrin.

## Graphical abstract

**Figure:**
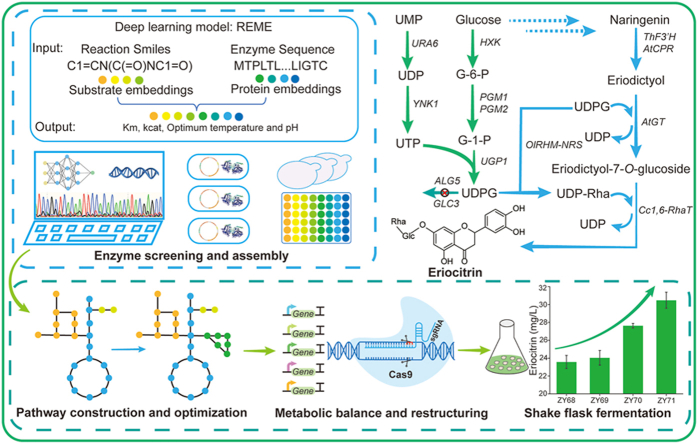

## 1 Introduction

Flavonoids are ubiquitous in plant-derived foods and exhibit diverse biological activities, including antioxidant, anti-inflammatory, lipid-lowering, and anticancer effects [[1], [2], [3], [4]]. However, their extremely low water solubility severely restricts bioavailability and application potential [5]. Glycosylation serves as a common strategy to significantly enhance water solubility, augment bioactivity, and reduce toxicity of flavonoids [6,7]. For instance, glycosylated hesperetin yields neohesperidin, which serves as a precursor for the novel sweetener neohesperidin dihydrochalcone [8]; the water solubility of puerarin was enhanced 14-fold through glucosylation [9]. Eriocitrin is a bioactive flavanone-7-*O*-disaccharide predominantly found in Rutaceae plants and demonstrates significant potential for nutraceutical and pharmaceutical applications [10]. Nevertheless, traditional production methods face significant bottlenecks: plant extraction suffers from limited scalability due to low abundance, complex processing, and agricultural dependency, while chemical synthesis is hampered by industrial constraints, including toxic reagents, harsh conditions, and solvent contamination [11,12]. In contrast, microbial biosynthesis utilizing engineered *S*. *cerevisiae* cell factories provides a scalable industrial platform. This platform has been successfully applied to produce a variety of high-value natural products, such as flavonoids [13], terpenoids [14], and phenolic compounds [15]. By overcoming the constraints of traditional methods, this approach provides key advantages in process controllability, cost-effectiveness, and environmental sustainability.

**Fig. 1:**
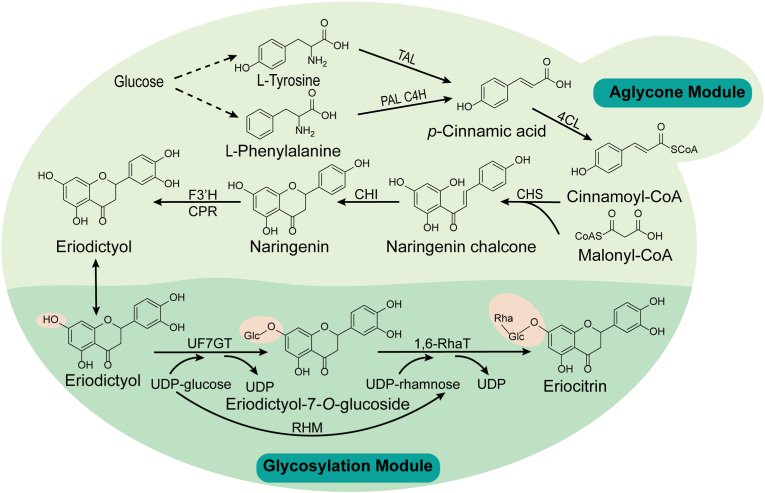
The *de novo* biosynthetic pathway of eriocitrin. The pathway is divided into two modules: the aglycone eriodictyol module and the glycosylation module. Aglycone module: Glucose is metabolized via endogenous pathways to form the aromatic amino acids l-phenylalanine and l-tyrosine. l-Phenylalanine is converted into *p*-coumaric acid by phenylalanine ammonia-lyase (PAL) and cinnamate-4-hydroxylase (C4H), while l-tyrosine is directly transformed into *p*-coumaric acid by tyrosine ammonia-lyase (TAL). *p*-Coumaric acid is subsequently catalyzed sequentially by 4-coumarate: CoA ligase (4CL), chalcone synthase (CHS), and chalcone isomerase (CHI) to yield naringenin. Naringenin is then converted into eriodictyol through the coordinated action of flavonoid 3′-hydroxylase (F3′H) and its cognate cytochrome P450 reductase (CPR). Glycosylation module: Eriodictyol is glucosylated by a flavonoid-7-*O*-glucosyltransferase (UF7GT) to produce eriodictyol-7-*O*-glucoside. This intermediate is further glycosylated by a 1,6-rhamnosyltransferase (1,6-RhaT), using UDP-rhamnose as the sugar donor, leading to the formation of eriocitrin. Notably, UDP-rhamnose is generated from UDP-glucose via catalysis by rhamnose synthase (RHM).

The biosynthesis of eriocitrin comprises two key stages: the synthesis of aglycone eriodictyol and the subsequent glycosylation process (Fig. 1). The eriodictyol biosynthesis originates from the phenylpropanoid pathway. l-Tyrosine is converted to *p*-coumaric acid (*p*-CA) catalyzed by tyrosine ammonia-lyase (TAL) [16]. Alternatively, l-phenylalanine is first deaminated to cinnamic acid by phenylalanine ammonia-lyase (PAL), subsequently being hydroxylated to *p*-CA by cinnamate 4-hydroxylase (C4H). Then, *p*-CA is activated to *p*-coumaroyl-CoA by 4-coumarate-CoA ligase (4CL) [15]. Subsequently, *p*-coumaroyl-CoA undergoes condensation with three malonyl-CoA molecules, catalyzed sequentially by chalcone synthase (CHS) and chalcone isomerase (CHI), yielding naringenin [13,17]. Next, the P450 enzyme flavone 3′-hydroxylase (F3′H) and its cognate cytochrome P450 reductase (CPR) catalyze the conversion of naringenin into eriodictyol [18]. Glycosylation of eriodictyol proceeds in two sequential steps. First, flavonoid-7-*O*-glucosyltransferase (UF7GT) specifically transfers a glucose moiety from UDP-glucose onto the 7-hydroxyl group, forming eriodictyol-7-*O*-glucoside [19]. Second, UDP-rhamnose synthase (RHM) catalyzes the conversion of UDP-glucose to UDP-rhamnose [20]. UDP-rhamnose then serves as the substrate for 1,6-rhamnosyltransferase (1,6-RhaT), which transfers a rhamnose moiety to the C6 position of the glucoside, ultimately synthesizing eriocitrin [21].

The current investigation of eriocitrin biosynthesis predominantly focuses on three critical elements: Precursor compounds (naringenin and eriodictyol) [22,23]; Sugar donors (UDP-glucose and UDP-rhamnose) [24,25]; Catalytic enzymes (glucosyltransferase and rhamnosyltransferase) [26,27]. Regarding the synthesis of flavonoid aglycones, Ru et al. engineered *Yarrowia lipolytica* to achieve high-yield naringenin (8.65 g/L) [28], while Yue et al. enhanced eriodictyol titers to 6.8 g/L in *Y*. *lipolytica* via NADPH regeneration system optimization [29]. Regarding the supply of sugar donors, a novel UDP-rhamnose regeneration system was constructed by Pei et al. using a two-enzyme reaction system, which could enhance the flux towards rhamnose-containing flavonoids like quercetin-3-*O*-rhamnoside [30]. In the category of catalytic enzymes, Yuan et al. identified six flavonoid-7-*O*-glucosyltransferases in pomelo via transcriptome analysis that exhibited promiscuous activity towards a wide range of flavonoid substrates [31]. Xiao et al. pioneered the first microbial platform for eriocitrin biosynthesis in *S. cerevisiae* by supplementing eriodictyol and optimizing UDP-rhamnose regeneration coupled with UDP-glucose synthesis, achieving a titer of 131.3 mg/L [21]. However, industrializing such integrated platforms still poses challenges: (1) Insufficient enzyme specificity: The majority of glucosyltransferases demonstrate low catalytic specificity for eriodictyol; (2) UDP-rhamnose scarcity: The absence of endogenous rhamnose pathways severely limits the activated sugar donor supply; (3) Metabolic resource competition: UDP-glucose allocation between cell wall biogenesis and eriocitrin synthesis generates carbon flux competition, resulting in growth-production trade-offs.

This study utilized *S*. *cerevisiae* as a chassis organism to establish a platform strain for *de novo* eriocitrin biosynthesis from glucose through combinatorial metabolic engineering. First, to verify the feasibility of the eriocitrin glycosylation process, an integrated approach of bioinformatics and deep learning was employed to identify two highly efficient UF7GTs: *Camellia sinensis* (CsGT) and *Arabidopsis thaliana* (AtGT). Second, given that the synthesis of eriocitrin requires substantial glycosyl donors, we therefore optimized the UDP-rhamnose and UDP-glucose modules by reconstructing the endogenous UDP-rhamnose synthesis pathway and overexpressing *YNK1* and *URA6* to enhance UDP-glucose supply, achieving an eriocitrin yield of 147.9 mg/L from eriodictyol. Then, to achieve *de novo* synthesis of eriocitrin, a strain capable of biosynthesizing eriodictyol (the aglycone of eriocitrin) was constructed from a high-yielding naringenin producer by screening optimal F3′H/CPR pairs, promoter engineering, and pathway integration, achieving a titer of 237.8 mg/L. Ultimately, through the integration of previous glycosylation optimization strategies into this high-eriodictyol-producing strain, we accomplished the first *de novo* synthesis of eriocitrin in *S. cerevisiae*, reaching a titer of 30.5 mg/L. This work not only accomplished the *de novo* synthesis of eriocitrin but also established a scalable framework for the glycosylation of subsequent natural compounds.

## 2 Materials and methods

### 2.1 Chemicals and reagents

Flavonoid standards were purchased from Yuanye Bio-Technology (Shanghai, China). Enzymes and Kits (Phanta Max Master Mix, Green Taq Mix, One Step Cloning Kit, DNA Gel Extraction Kit, and Plasmid Mini Kit) were sourced from Vazyme Biotech (Nanjing, China). Ampicillin was supplied by Solarbio Science & Technology (Beijing, China). Tryptone and Yeast Extract were obtained from Oxoid (Hampshire, UK). All codon-optimized genes of plant origin were chemically synthesized by Sangon Biotech (Shanghai, China) and are listed in Table S1 (codon-optimized exogenous gene sequences are listed in Table S2). All primers were synthesized by Sangon Biotech (Shanghai, China); see Table S3 for details.

### 2.2 Plasmid and strain construction

All plasmids were constructed in *Escherichia coli* JM109 and are cataloged in Table S4. Linearized pY26, pRS423, and pRS424 vectors served as backbones for expression cassette assembly via overlap extension PCR [21]. CRISPR guide RNAs targeting designated genomic loci were designed using CHOPCHOP (http://chopchop.cbu.uib.no/; Table S5). All plasmids were assembled through homologous recombination (ClonExpress MultiS One Step Cloning Kit). Recombinant plasmids were initially screened by *E. coli* colony PCR and verified by sequencing.

*S*. *cerevisiae* strains and genotypes are summarized in Table S6. All genetic modifications employed CEN.PK2-1D-derived strain C800 (*MATα*; *ura3-52*; *trp1-289*; *leu2-3112*; *his3Δ1*; *MAL2-8C*; *SUC2*; *gal80::G418*) as the parental strain. Native elements (promoters, terminators, genes) were amplified from C800 genomic DNA, whereas codon-optimized heterologous genes were derived from synthetic fragments. Chromosomal manipulations, including overexpression, deletion, and integration, were performed using CRISPR/Cas9. Recombinant DNA (plasmids, integration fragments, knockout cassettes) was transformed into yeast using the LiAc/ssDNA/PEG method. Transformants were selected on SD medium at 30 °C for 3–5 days, and PCR-confirmed positive clones were subsequently cultured in YPD medium.

### 2.3 Culture media and conditions

*E*. *coli* JM109 was cultured in LB medium (10 g/L tryptone, 5 g/L yeast extract, 10 g/L NaCl, 100 μg/mL ampicillin). *S*. *cerevisiae* strains were maintained in YPD (20 g/L tryptone, 10 g/L yeast extract, 20 g/L glucose). Transformants were screened on SD medium (20 g/L glucose, 1.74 g/L yeast nitrogen base without amino acids, 5 g/L ammonium sulfate) supplemented as required with the appropriate amino acids or uracil (50 mg/L) to complement auxotrophies. Positive clones were then transferred to YPD plates containing 1 g/L 5-fluoroorotic acid to select for gRNA plasmid curing and *URA3* prototrophy restoration.

For shake flask fermentation, a single colony of each yeast strain was inoculated into 5 mL of liquid SD medium and cultured at 30 °C with shaking at 200 rpm for 20 h to prepare the seed culture. Subsequently, a 1 % (v/v) inoculum from the seed culture was transferred to 25 mL of YPD medium in a 250 mL flask. The culture was incubated at 30 °C with shaking at 220 rpm. After 12 h of fermentation, 300 mg/L eriodictyol was added (except for the *de novo* strains). The cultures were harvested after 96 h for HPLC analysis.

### 2.4 UF7GT bioinformatics analysis

Bioinformatic analysis involved retrieving 30 flavonoid glucosyltransferase sequences from the NCBI database (https://www.ncbi.nlm.nih.gov/) for phylogenetic analysis. A maximum likelihood tree was constructed using MEGA11 software (1000 bootstrap replicates) and visualized via the interactive Tree of Life (iTOL) online tool [32] (https://itol.embl.de/). Open reading frames (ORF) in the 30 plant-derived UF7GT sequences were predicted with ORFfinder (https://www.ncbi.nlm.nih.gov/orffinder/). Physicochemical properties of amino acid sequences were analyzed using ExPASy-ProtParam (https://web.expasy.org/protparam/). Multiple sequence alignment was performed and visualized using DNAMAN and GENEDOC, respectively. The enzyme function of UDP-glycosyltransferases was predicted using the REME (reaction enzyme mining and evaluation)database [33] (https://reme.biodesign.ac.cn/).

### 2.5 Analytical methods

Quantitative analysis of flavonoids and flavonoid glycosides was performed by HPLC [21]. The cell culture (0.5 mL) was mixed with an equal volume of methanol, vortexed for 5 min, and centrifuged at 12,000 rpm for 2 min, after which the supernatant was filtered through a 0.22 μm membrane and stored at −20 °C until analysis. Chromatographic separation was employed on a Shimadzu Sustain C18 column (250 mm × 4.6 mm, 5 μm) maintained at 30 °C on an HPLC system with diode array detection at 284 nm. Samples (10 μL) were injected at 1 mL/min flow rate using mobile phases of water with 0.1 % (v/v) formic acid (A) and acetonitrile (B) under the gradient program: 0–10 min with a linear increase from 10 % to 40 % B, 10–20 min increasing to 50 % B, 20–25 min decreasing to 10 % B, 25–30 min maintaining at 10 % B.

### 2.6 Statistical analysis

Data are presented as the mean ± standard deviation from three independent replicates. Statistical significance was determined by one-way ANOVA and Student's *t*-tests, with significance levels indicated as ∗*p* < 0.05 and ∗∗*p* < 0.01.

## 3 Results and discussion

### 3.1 Deep learning-driven discovery and characterization of UF7GT

**Fig. 2:**
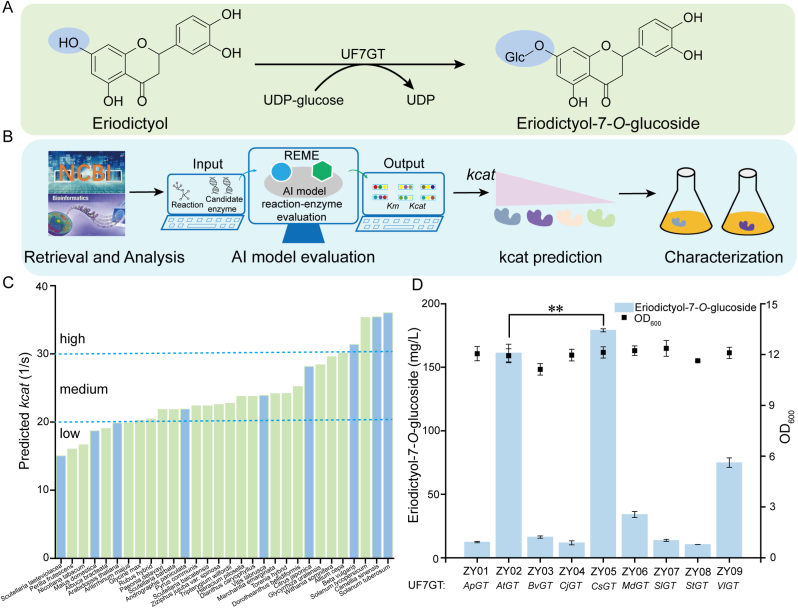
Mining and characterization of UF7GTs. (A) Schematic of the eriodictyol-7-*O*-glucoside biosynthetic pathway in engineered *S. cerevisiae*. (B) Workflow for UF7GT mining and characterization. Relevant UF7GT sequences were retrieved from the NCBI database and subjected to preliminary bioinformatic analysis. The REME platform was then employed for comprehensive reaction and enzyme evaluation, with enzyme selection for experimental validation based on predicted Kcat values. (C) The kcat prediction for UF7GTs using the TurNuP deep learning model within REME. Based on the predicted Kcat values, the UF7GTs were stratified into high, medium, and low intervals, with three UF7GTs selected from each interval for experimental validation. Blue bars indicate the UF7GTs selected for further experimental validation. (D) Production of eriodictyol-7-*O*-glucoside by different UF7GTs following supplementation with 300 mg/L eriodictyol. Bar graph shows eriodictyol-7-*O*-glucoside titers, and scatter plot represents corresponding cell biomass. ApGT: *Andrographis paniculata* glycosyltransferase, AtGT: *Arabidopsis thaliana* glycosyltransferase, BvGT: *Beta vulgaris* glycosyltransferase, CjGT: *Citrus japonica* glycosyltransferase, CsGT: *Camellia sinensis* glycosyltransferase, MdGT: *Malus x domestica* glycosyltransferase, SlGT: *Scutellaria laeteviolacea* glycosyltransferase, StGT: *Solanum tuberosum* glycosyltransferase, VlGT: *Vitis labrusca* glycosyltransferase. Statistical analysis was conducted using the Student's *t*-test (∗∗*p* value < 0.01; sample size, *n* = 3).

Eriocitrin is biosynthesized from eriodictyol through a two-step glycosylation pathway. The initial glycosylation step is catalyzed by UF7GT, converting eriodictyol into eriodictyol-7-*O*-glucoside (Fig. 2A). However, the majority of UF7GTs exhibit limited specificity toward eriodictyol and frequently catalyze off-target glycosylation of structural analogues, such as naringenin and hesperetin [34]. This enzymatic promiscuity reduces final product purity and overall pathway efficiency [35]. To improve the discovery of enzymes with enhanced catalytic performance, the integration of bioinformatics and artificial intelligence (machine learning and deep learning) has become a major focus in enzyme mining [36,37]. To this end, we established an integrated computational-experimental framework for enzyme mining (Fig. 2B). The workflow began with the retrieval of thirty cross-species UF7GT genes from the NCBI database, which were then subjected to bioinformatic analysis. Open reading frame prediction indicated that these UF7GT genes span 1344–1786 bp. Subsequent analysis of the deduced protein physicochemical properties revealed that they encode 450**–**505 amino acids with a theoretical isoelectric point (pI) below 7 (Table S7). Phylogenetic analysis of 30 UF7GT sequences revealed evolutionarily distant relationships among *A*. *thaliana*, *Glycyrrhiza uralensis*, *Paeonia delavayi*, and *Osteospermum ecklonis* relative to other plant species. Major clades contained lineage-specific groups of sequences (Fig. S1). Multiple sequence alignment confirmed that all UF7GT isoforms contain the characteristic GT-B fold and the plant-conserved PSPG motif (Fig. S2). As previously reported, the UDP-sugar binding region was highly conserved across these UGTs [38].

To assess UF7GT activity, we employed the REME platform [33]. This platform integrates six cutting-edge methods rooted in deep learning techniques (ESP, DLKcat, TurNuP, DeepET, KM_prediction, and EpHod) to construct a multidimensional evaluation model for identifying eriodictyol-7-*O*-glucoside synthesis reactions. Notably, the TurNuP model leverages graph neural networks (GNNs) and convolutional neural networks (CNNs) to achieve precise kcat prediction through transfer learning on millions of enzymatic data points (Fig. 2C). Based on the predicted kcat values, candidate enzymes assigned to high (CsGT, StGT, BvGT), medium (CjGT, VlGT, ApGT), and low (AtGT, MdGT, SlGT) activity tiers were selected for experimental validation. The corresponding genes were cloned into pY26 vectors and transformed into the glycoside hydrolase-deficient strain E033 [21], generating strains ZY01–ZY09. During the fermentation process, 300 mg/L of eriodictyol was added to the culture media as a substrate. All nine enzymes exhibited UF7GT catalytic activity (Fig. 2D and Fig. S3). Specifically, the highest catalytic efficiency was observed for *C*. *sinensis* CsGT (strain ZY05) and *A*. *thaliana* AtGT (strain ZY02), producing 179.2 and 161.2 mg/L of eriodictyol-7-*O*-glucoside, respectively. Overall, an integrated approach of rational computation, analysis, and screening enabled the identification of the two most active enzymes, CsGT and AtGT, from a series of candidate enzymes in an engineered strain.

### 3.2 Metabolic reconstitution of UDP-rhamnose for biosynthesis of eriocitrin

**Fig. 3:**
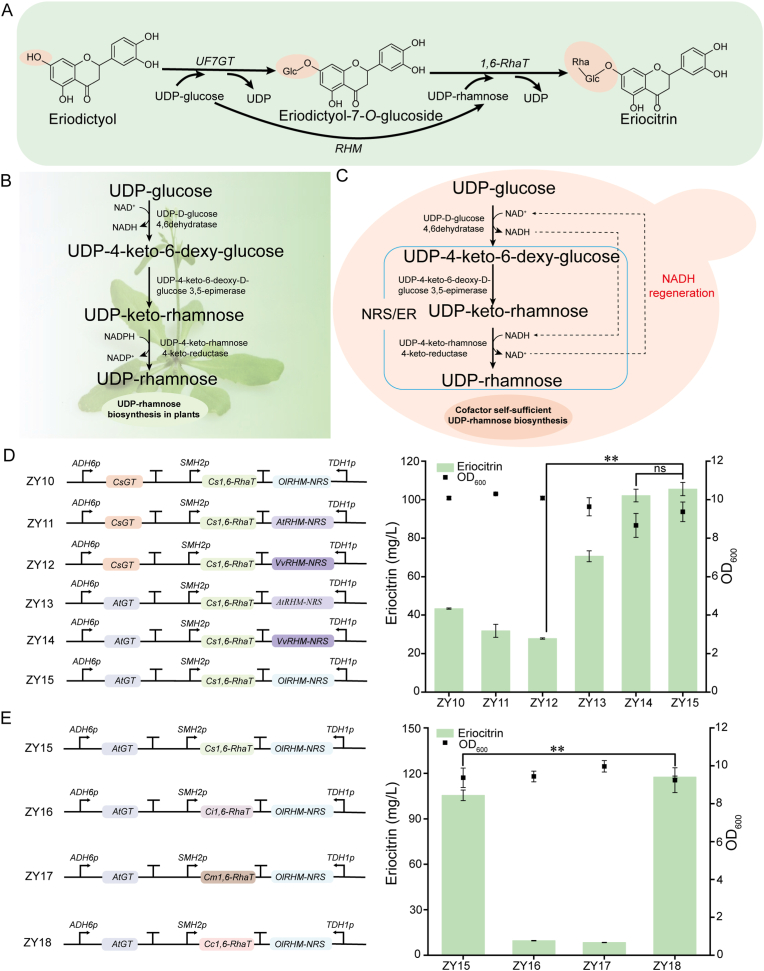
Engineering of eriocitrin biosynthesis. (A) Biosynthetic pathway from eriodictyol to eriocitrin, involving three enzymes (UF7GT, RHM, and 1,6-RhaT) and two glycosyl donors (UDP-glucose and UDP-rhamnose). (B) The native UDP-rhamnose biosynthesis pathway in plants utilizes two cofactors: NAD^+^ and NADPH. (C) Engineered self-sufficient UDP-rhamnose pathway in *S. cerevisiae*. Through the construction of a fusion enzyme, RHM-NRS, only NAD^+^ is required as a cofactor, and it can be regenerated internally to achieve cofactor recycling. (D) Metabolic reconstitution of UDP-rhamnose supply for eriocitrin production. (E) Screening of heterologous rhamnosyltransferases for efficient eriocitrin synthesis. Statistical analysis was conducted using the Student's *t*-test (∗∗*p* value < 0.01; NS represents no significant difference; sample size, *n* = 3).

The second glycosylation step in eriocitrin biosynthesis is catalyzed by 1,6-RhaT. This enzyme uses eriodictyol-7-*O*-glucoside as the glycosyl acceptor and UDP-rhamnose as the glycosyl donor. UDP-rhamnose is synthesized from UDP-glucose by RHM (Fig. 3A). However, *S*. *cerevisiae* lacks an endogenous UDP-rhamnose biosynthesis pathway. Current strategies that rely on exogenous UDP-rhamnose supplementation or chemically synthesized donors are economically unviable due to high costs, inefficient transmembrane transport, and metabolic imbalance [30,39]. To address this limitation, we engineered the yeast metabolic network by introducing a heterologous plant-derived UDP-rhamnose biosynthetic pathway.

In plants, UDP-rhamnose is biosynthesized from UDP-glucose via three sequential reactions catalyzed by the multifunctional enzyme RHM (Fig. 3B), which integrates the following activities: UDP-d-glucose 4,6-dehydrogenase; UDP-4-keto-6-deoxy-d-glucose 3,5-epimerase; and UDP-4-keto-rhamnose 4-keto-reductase [40]. The N-terminal domain of RHM converts UDP-glucose to UDP-4-keto-6-deoxyglucose, while the C-terminal domain converts this intermediate into UDP-rhamnose through epimerization and reduction [20]. This process requires two cofactors (NAD^+^ and NADPH), and the complex cofactor regeneration system impedes industrial application [41]. To address this, the RHM-NRS fusion enzyme was constructed by directly fusing the truncated UDP-d-glucose 4,6-dehydratase catalytic domain of RHM with the UDP-4-keto-6-deoxy-d-glucose 3,5-epimerase/UDP-4-keto-rhamnose 4-keto-reductase (NRS/ER) domain from *A. thaliana* [42]. This chimeric enzyme utilizes NADH as a cofactor and establishes an NADH regeneration system during catalysis (Fig. 3C). Three truncated RHMs from *Ornithogalum longebracteatum* (OlRHM), *A*. *thaliana* (AtRHM), and *Vitis vinifera* (VvRHM) were individually fused to the *A*. *thaliana* NRS/ER. These fusion constructs were then combinatorially expressed with two 7-*O*-glucosyltransferases (CsGT, AtGT) and a *Citrus sinensis* 1,6-rhamnosyltransferase (Cs1,6-RhaT) in the engineered strain E033, generating strains ZY10–ZY15 (Fig. 3D). All engineered strains demonstrated the ability to synthesize eriocitrin (Fig. S4), with strain ZY15 (AtGT-Cs1,6-RhaT-OlRHM-NRS) yielding the highest titer at 105.5 mg/L. Notably, AtGT-containing combinations (ZY13–ZY15) outperformed the CsGT-based combinations (ZY10–ZY12), demonstrating nonlinear synergistic effects among the multi-enzyme modules [43].

To further optimize eriocitrin production, we screened three plant-derived 1,6-RhaTs: Ci1,6-RhaT (*Chrysanthemum indicum*), Cm1,6-RhaT (*Citrus maxima*), and Cc1,6-RhaT (*Citrus × clementina*). Each 1,6-RhaT was assembled with AtGT and OlRHM-NRS and expressed in engineered *S. cerevisiae*, yielding strains ZY16–ZY18. Comparative analysis revealed that Cc1,6-RhaT exhibited the highest catalytic efficiency. The optimized strain ZY18 (AtGT-OlRHM-NRS-Cc1,6-RhaT) produced 117.5 mg/L eriocitrin, representing an 11.37 % increase over ZY15 (Fig. 3E).

### 3.3 Engineering UDP-glucose supply to optimize eriocitrin biosynthesis

**Fig. 4:**
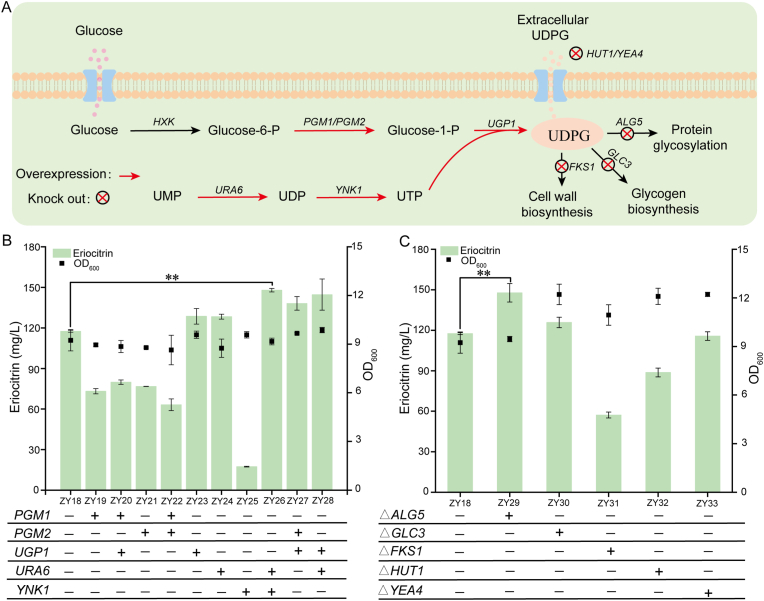
Engineering UDP-glucose metabolism to enhance eriocitrin production. (A) Schematic representation of UDP-glucose biosynthesis, consumption, and transport in *S. cerevisiae*. Red arrows denote gene overexpression, and red crosses indicate gene knockout. (B) Eriocitrin production following overexpression of UDP-glucose synthesis genes. A plus sign denotes gene overexpression, while a minus sign indicates without overexpression. (C) Eriocitrin titers in strains with knockout of UDP-glucose consumption or transporter genes. A plus sign represents gene knockout, and a minus sign indicates without knockout of the gene. Statistical analysis was conducted using the Student's *t*-test (∗∗*p* value < 0.01; sample size, *n* = 3).

UDP-glucose, the core glycosyl donor for eriocitrin biosynthesis, is regulated by three interconnected modules: synthesis, consumption, and transport (Fig. 4A). Its biosynthesis integrates two metabolic precursors: glucose-1-phosphate (G1P) from the nucleotide sugar pathway and uridine triphosphate (UTP) from pyrimidine metabolism. G1P is generated from glucose-6-phosphate by phosphoglucomutases (PGM1 and PGM2), and UTP is produced through sequential phosphorylation catalyzed by uridylate kinase (URA6) and nucleoside diphosphate kinase (YNK1). UDP-glucose pyrophosphorylase (UGP1) then catalyzes the condensation of G1P and UTP to form UDP-glucose [44]. To augment precursor supply, the genes encoding *PGM1*, *PGM2*, *URA6*, *YNK1*, and *UGP1* were overexpressed in strain ZY18, generating engineered strains ZY19–ZY28 (Fig. 4B). Among strains with single-gene overexpression, only ZY23 (overexpressing *UGP1*; 128.6 mg/L) and ZY24 (overexpressing *URA6*; 128.3 mg/L) produced more eriocitrin than strain ZY18 (117.5 mg/L). These results indicate that the enzymatic steps catalyzed by UGP1 (converting G1P to UDP-glucose) and URA6 (involved in the conversion of UMP to UDP) are critical in the UDP-glucose biosynthesis pathway. This further suggests that *UGP1* and *URA6* likely function synergistically [45] or complementarily [46] to enhance the intracellular UDP-glucose pool, thereby increasing metabolic flux toward eriocitrin synthesis. We propose the following mechanistic explanation: overexpression of *URA6* elevates intracellular UDP levels, which promotes subsequent UTP generation. Since UTP serves as the substrate for the UGP1-catalyzed reaction (UTP + G1P → UDP-glucose + PPi), its increased availability amplifies the effect of *UGP1* overexpression, resulting in a coordinated boost in UDP-glucose synthesis flux. Combinatorial overexpression experiments further supported these findings. Compared to ZY18, strain ZY26 (co-expressing *URA6* and *YNK1*) produced 147.9 mg/L eriocitrin (25.87 % increase), ZY27 (co-expressing *UGP1* and *PGM2*) yielded 138.1 mg/L (17.53 % increase), and ZY28 (co-expressing *URA6* and *UGP1*) reached 144.6 mg/L (23.06 % increase). These results collectively demonstrate that engineering key nodes in the UDP-glucose supply pathway can significantly enhance eriocitrin production.

As a central metabolic hub in *S. cerevisiae*, UDP-glucose serves diverse cellular processes, including cell wall biosynthesis (*β*-1,3-glucan elongation by FKS1) [47], glycogen accumulation (branching by GLC3) [48], protein N-glycosylation (initial glucose addition to lipid-linked oligosaccharides by ALG5) [49], trehalose synthesis, and galactose metabolism. This multidirectional flux diverts UDP-glucose toward competing pathways, an effect exacerbated by efflux through membrane transporters (HUT1 and YEA4) [50], collectively limiting glycosylation efficiency. To address this bottleneck, key genes from consumption pathways (*FKS1*, *GLC3*, *ALG5*) and efflux transporters (*HUT1*, *YEA4*) were disrupted, constructing strains ZY29**–**ZY33 (Fig. 4C). Compared to strain ZY18, strains ZY29 (*ΔALG5*) and ZY30 (*ΔGLC3*) achieved higher eriocitrin titers of 147.8 mg/L and 125.8 mg/L, representing yield increases of 25.78 % and 7.06 %, respectively. In contrast, deletion of *FKS1*, *YEA4*, or *HUT1* did not enhance eriocitrin production. These yeast deletion mutants may retain functional isozymes or activate compensatory pathways to sustain cell wall biosynthesis and glycogen metabolism, thereby supporting normal cell growth [51]. For instance, studies have demonstrated that deletion of *FKS1* induces chitin biosynthesis as a compensatory mechanism in yeast [52]. This process consumes UDP-glucose, which may consequently limit the precursor availability for eriocitrin synthesis. Collectively, enhancing UDP-glucose supply is a validated strategy for microbial glycoside synthesis. Our findings specifically demonstrate that coordinated engineering of UDP-glucose biosynthesis and precursor flux allocation is critical for achieving high-efficiency eriocitrin biosynthesis in yeast.

### 3.4 *De novo* biosynthesis of eriodictyol in an engineered *S. cerevisiae* with enhanced naringenin

**Fig. 5:**
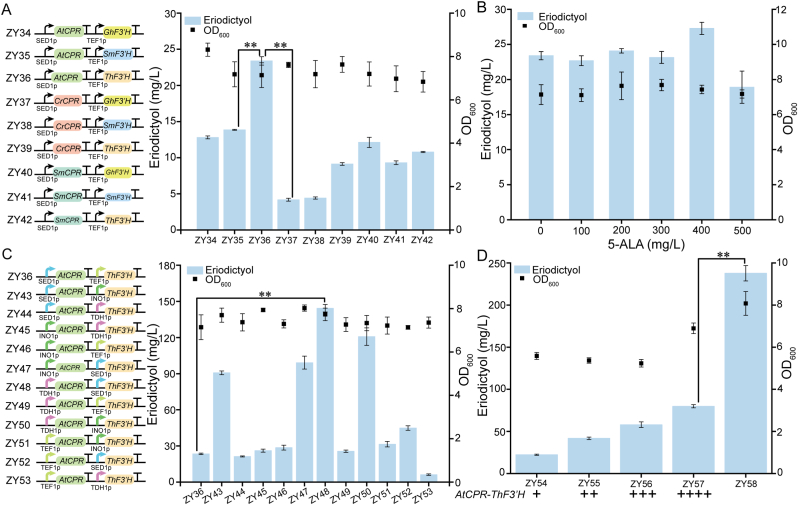
Engineering of *de novo* eriodictyol biosynthesis. (A) Screening of heterologous *F3′H* and *CPR* gene combinations for constructing eriodictyol-producing strains. (B) Effect of supplementing different concentrations of 5-ALA on eriodictyol production. (C) Effect of using promoters with different strengths on eriodictyol synthesis. (D) Effect of increasing gene copy number on eriodictyol yield. Statistical analysis was conducted using the Student's *t*-test (∗∗*p* value < 0.01; sample size, *n* = 3).

To achieve the *de novo* synthesis of eriocitrin, we first established a biosynthetic pathway for its aglycone, eriodictyol, which uses naringenin as its direct precursor. We introduced heterologous cytochrome P450 activity into the high-yielding naringenin chassis strain ZY00 to enable *de novo* eriodictyol production. Although co-expression of heterologous F3′H and CPR mediates the conversion of naringenin to eriodictyol, catalytic efficiency exhibits significant variation depending on the enzyme source organism. To identify optimal enzyme combinations, F3′H orthologs from *Silybum marianum* (SmF3′H), *Gerbera hybrida* (GhF3′H), and *Tricyrtis hirta* (ThF3′H) were systematically co-expressed with CPR isoforms from *S. marianum* (SmCPR), *A*. *thaliana* (AtCPR), and *Catharanthus roseus* (CrCPR) in engineered strains ZY34**–**ZY42 (Fig. 5A). Strain ZY36 (ThF3′H-AtCPR) produced 23.4 mg/L eriodictyol, which is consistent with previous reports of the high efficiency of the ThF3′H-AtCPR pairing [53]. However, the low eriodictyol yield, along with the substantial accumulation of naringenin (Fig. S5), indicated suboptimal catalysis.

Given that heme, the essential P450 cofactor, is biosynthesized from 5-aminolevulinic acid (5-ALA), we supplemented exogenous 5-ALA to enhance heme supply. This strategy successfully enhanced eriodictyol production (Fig. 5B). Supplementation with 400 mg/L 5-ALA resulted in a titer of 27.3 mg/L, corresponding to a 16.7 % increase compared to strain ZY36. However, when the 5-ALA concentration was increased to 500 mg/L, the eriodictyol yield decreased, indicating potential substrate inhibition or cellular toxicity at elevated concentrations. Although 5-ALA supplementation led to a modest improvement in production, the overall increase remained limited, implying that factors other than heme supply were the principal constraints on pathway efficiency. We therefore hypothesized that suboptimal F3′H/CPR binding affinity, which is governed by their strict 1:1 stoichiometric requirement, constitutes a key bottleneck in electron transfer. This view is supported by studies showing that optimized F3′H: CPR expression ratios enhance eriodictyol titers in *S. cerevisiae* [23]. Given promoters' fundamental role in governing transcriptional initiation kinetics and efficiency [54], we employed promoter engineering to achieve a precise stoichiometric balance between ThF3′H and AtCPR expression. We constructed strains ZY43–ZY53 through combinatorial screening of endogenous *S. cerevisiae* promoters with varying strengths: TDH1p (strongest) > INO1p > SED1p > TEF1p (weakest) [55]. Strain ZY48 (TDH1p-AtCPR-SED1p-ThF3′H) achieved 144.3 mg/L eriodictyol, representing a 516.67 % increase over Strain ZY36 (Fig. 5C), demonstrating that regulating the expression ratio of F3′H to CPR through promoter strength effectively enhanced the synthesis of eriodictyol.

To enhance expression stability, the optimized expression cassette (TDH1p-AtCPR-SED1p-ThF3′H) was genomically integrated at the neutral locus *XII-2*. Unexpectedly, integration severely reduced eriodictyol production to 22.3 mg/L, demonstrating the superiority of multi-copy plasmid systems over single-copy genomic integrations for high-level expression. Subsequently, we increased genomic copy numbers (strains ZY54–Z57), yet the four-copy strain ZY57 achieved only 79.9 mg/L, still substantially lower than that achieved by the plasmid system (144.3 mg/L). These results unequivocally establish gene dosage as a critical determinant of expression levels [56]. To circumvent this limitation, the plasmid harboring TDH1p-AtCPR-SED1p-ThF3′H was introduced into ZY57, generating strain ZY58. Fermentation analysis revealed a 237.8 mg/L eriodictyol titer in ZY58 (Fig. 5D), representing a 64.8 % increase over ZY48 and a 2.98-fold enhancement relative to ZY57.

### 3.5 *De novo* biosynthesis pathway engineering for eriocitrin production in *S. cerevisiae*

**Fig. 6:**
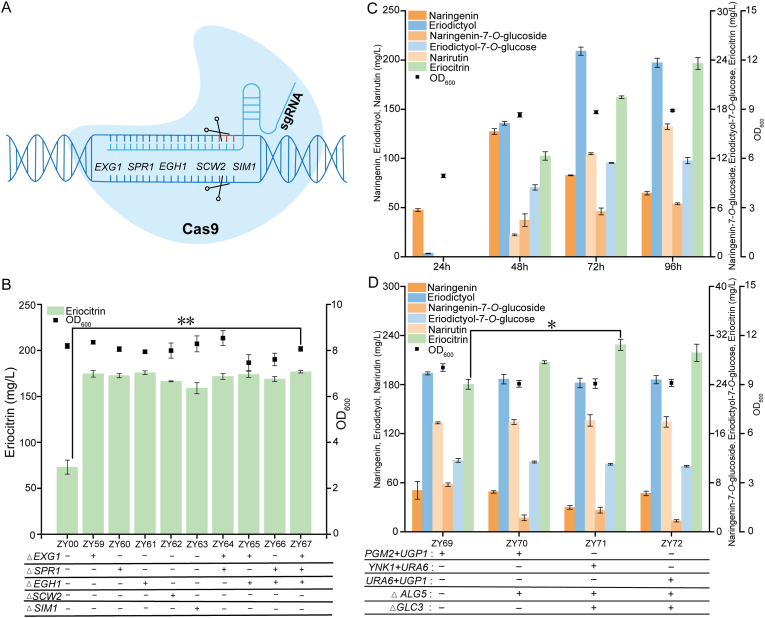
Construction and optimization of a *de novo* eriocitrin biosynthetic pathway. (A) Schematic of CRISPR-Cas9-mediated knockout of endogenous glycoside hydrolase genes in *S. cerevisiae*. (B) Residual eriocitrin after incubation of engineered strains supplemented with 200 mg/L eriocitrin as substrate. A plus sign indicates gene knockout, and a minus sign indicates without knockout of the gene. (C) Metabolite accumulation and cell growth profile of engineered strain ZY68 during *de novo* eriocitrin biosynthesis. (D) Systematic optimization of eriocitrin production through genetic modifications. A plus sign denotes gene overexpression or knockout, a minus sign represents without overexpression or knockout. Statistical analysis was conducted using the Student's *t*-test (∗∗*p* value < 0.01; ∗*p* value < 0.05; sample size, *n* = 3).

Previous studies have demonstrated that *S*. *cerevisiae* endogenously harbors multiple flavonoid glycoside hydrolases, which can catalyze the hydrolysis of flavonoid glycosides, thereby compromising the accumulation of target products [57,58]. To construct a suitable chassis cell for eriocitrin biosynthesis and prevent its degradation during bioproduction, ZY00 was engineered using CRISPR/Cas9 gene editing technology (Fig. 6A). Based on literature reports, five genes potentially involved in flavonoid glycoside hydrolysis were identified, including *EXG1*, *SPR1*, *EGH1*, *SCW2*, and *SIM1* [59]. Single-gene and combinatorial knockout strategies were employed to generate engineered strains ZY59**–**ZY67. To evaluate the knockout effects, 200 mg/L of eriocitrin was added to the culture medium as a substrate during the fermentation process, and the residual eriocitrin was quantified to assess degradation activity (Fig. 6B). Fermentation analysis revealed striking differences in eriocitrin stability between strains. The control strain ZY00 (without glycoside hydrolase knockout) retained only 73.1 mg/L eriocitrin, whereas all engineered strains with glycoside hydrolase deletions maintained eriocitrin levels above 150 mg/L. These results clearly demonstrate that targeted knockout of glycoside hydrolase genes effectively prevents eriocitrin degradation during biosynthesis. Notably, strain ZY67 (lacking *EXG1*, *SPR1*, and *EGH1*) exhibited optimal performance with 176.8 mg/L residual eriocitrin. This optimal knockout combination was subsequently introduced into the high-eriodictyol-producing strain ZY58.

Into the resulting glycoside hydrolyase-deficient strain ZY58 (*ΔEXG1*, *ΔSPR1*, and *ΔEGH1*), we introduced the previously optimized glycosylation module (AtGT, OlRHM-NRS, and Cc1,6-RhaT) to establish a *de novo* eriocitrin biosynthetic pathway, generating strain ZY68 (Fig. 6C). Time-course fermentation analysis revealed distinct metabolite accumulation dynamics. During the initial 24 h, only the precursor compounds naringenin and eriodictyol were detected and found to accumulate. Subsequently, synthesis of various glycosylated intermediates commenced, including naringenin-7-*O*-glucoside, eriodictyol-7-*O*-glucoside, and narirutin, along with the target product eriocitrin. Peak concentrations were attained at 96 h for all glycosylated compounds, with naringenin-7-*O*-glucoside reaching 6.5 mg/L, eriodictyol-7-*O*-glucoside reaching 11.7 mg/L, narirutin reaching 132.3 mg/L, and eriocitrin reaching 23.6 mg/L.

To further enhance eriocitrin production in strain ZY68, we introduced a series of previously validated genetic modifications aimed at strengthening the UDP-glucose supply, resulting in the construction of strains ZY69–ZY72 (Fig. 6D). Fermentation analysis revealed that the overexpression of *PGM2* and *UGP1* in ZY68 (strain ZY69) yielded an eriocitrin titer of 24.1 mg/L. Building on this modification, the additional knockout of *ALG5* in ZY69 (strain ZY70) further increased production to 27.6 mg/L. The co-overexpression of *URA6* and *UGP1*, combined with the knockout of *ALG5* and *GLC3* (strain ZY72), elevated the titer to 29.22 mg/L. Notably, the simultaneous overexpression of *YNK1* and *URA6*, along with the knockout of *ALG5* and *GLC3* (strain ZY71), achieved the highest eriocitrin production, reaching 30.5 mg/L. To our knowledge, this is the first report of *de novo* biosynthesis of eriocitrin in *S. cerevisiae*. However, strains ZY69–ZY72 generated substantial quantities of the by-product narirutin (greater than 130 mg/L), the accumulation of which significantly limited the eriocitrin yield. Narirutin is synthesized from its aglycone naringenin through two sequential glycosylation steps: glucosylation followed by rhamnosylation. This pathway competes with that of eriocitrin for the shared glycosyl donors. In future work, we aim to combine enzyme engineering with metabolic pathway optimization to minimize the formation of the byproduct narirutin and thereby enhance the production efficiency of eriocitrin.

In summary, this study establishes essential metabolic engineering strategies for achieving *de novo* biosynthesis of eriocitrin in *S. cerevisiae*. First, to overcome the low specificity of 7-*O*-glucosyltransferases toward eriodictyol, we combined bioinformatics with deep learning to identify efficient glucosyltransferases, enabling the initial glycosylation step from eriodictyol to eriodictyol-7-*O*-glucoside. Subsequently, to address UDP-rhamnose scarcity, we introduced a heterologous UDP-rhamnose biosynthetic pathway and optimized a two-enzyme reaction system, identifying AtGT, OlRHM-NRS, and Cc1,6-RhaT as the optimal combination to complete the glycosylation pathway. This system achieved an eriocitrin titer of 117.5 mg/L from eriodictyol. Since UDP-glucose serves as a key glycosylation precursor, we implemented a push-block strategy to enhance its supply by reinforcing biosynthesis and blocking competing pathways, thereby alleviating metabolic resource competition between cell growth and product synthesis. To accomplish *de novo* synthesis of eriocitrin, we then constructed the biosynthetic pathway for eriodictyol (the aglycone of eriocitrin) by screening optimal F3′H/CPR pairs, engineering promoters, and integrating pathway modules, resulting in a strain producing 237.8 mg/L eriodictyol. Finally, by integrating the optimized glycosylation pathway and enhanced UDP-glucose supply into the high-producing eriodictyol strain, we accomplished *de novo* biosynthesis of eriocitrin at a titer of 30.5 mg/L in *S. cerevisiae*. To our knowledge, this work represents the first report of *de novo* eriocitrin production in *S. cerevisiae*, providing a scalable platform for the sustainable microbial production of flavonoid glycosides.

## CRediT authorship contribution statement

**Qiyuan Lu:** Writing – original draft, Methodology, Investigation, Data curation, Conceptualization. **Xinjia Tan:** Methodology, Formal analysis, Data curation. **Siqi Zhang:** Methodology, Formal analysis, Conceptualization. **Yongtong Wang:** Visualization, Software, Formal analysis. **Yifei Zhao:** Methodology, Formal analysis. **Juan Liu:** Supervision, Formal analysis. **Fanglin Hu:** Formal analysis. **Shasha Zuo:** Software. **Jiaxu Chen:** Software. **Liusha Fan:** Software. **Shenghua Ding:** Supervision, Project administration. **Zhiqiang Xiao:** Writing – review & editing, Supervision, Funding acquisition, Conceptualization. **Yang Shan:** Supervision, Project administration, Funding acquisition, Conceptualization.

## Declaration of competing interest

The authors declare that they have no known competing financial interests or personal relationships that could have appeared to influence the work reported in this paper.

## References

[1] Zhao C., Wang F., Lian Y., Xiao H., Zheng J. (2020). Biosynthesis of citrus flavonoids and their health effects. Crit Rev Food Sci Nutr.

[2] Li H., Lyv Y., Zhou S., Yu S., Zhou J. (2022). Microbial cell factories for the production of flavonoids-barriers and opportunities. Bioresour Technol.

[3] Matsui K., Walker A.R. (2020). Biosynthesis and regulation of flavonoids in buckwheat. Breed Sci.

[4] Ruwanthi P., Ali R., Matt G., Jaspreet S. (2022). Oral delivery of hydrophobic flavonoids and their incorporation into functional foods: opportunities and challenges. Food Hydrocoll.

[5] Yuan J., Xiong R., Zhu T., Ni R., Fu J., Lou H., Cheng A. (2021). Cloning and functional characterization of three flavonoid *O*-glucosyltransferase genes from the liverworts *Marchantia emarginata* and *Marchantia paleacea*. J Plant Physiol.

[6] Nabavi S.M., Šamec D., Tomczyk M., Milella L., Russo D., Xu S., Shirooie S. (2020). Flavonoid biosynthetic pathways in plants: versatile targets for metabolic engineering. Biotechnol Adv.

[7] Slámová K., Kapešová J., Valentová K. (2018). "Sweet flavonoids": glycosidase-catalyzed modifications. Int J Mol Sci.

[8] Zou Y., Li X., Xin X., Xu H., Zhao G. (2024). Microbial-driven synthesis and hydrolysis of neohesperidin dihydrochalcone: biotransformation process and feasibility investigation. J Agric Food Chem.

[9] Li D., Park S., Shim J., Lee H., Tang S., Park C., Park K. (2004). In vitro enzymatic modification of puerarin to puerarin glycosides by maltogenic amylase. Carbohydr Res.

[10] Yao L., Liu W., Bashir M., Nisar M.F., Eriocitrin C. Wan (2022). A review of pharmacological effects. Biomed Pharmacother.

[11] Feng C., Chiko O., Yuichi O. (2022). Innovatively identifying naringin and hesperidin by using terahertz spectroscopy and evaluating flavonoids extracts from waste orange peels by coupling with multivariate analysis. Food Control.

[12] Xu H., Li Z., Wu Y., Luo D., Qiu L., Xie J., Li X. (2019). Advances on synthesis of flavonoid glycosides. Chin J Org Chem.

[13] Li H., Ma W., Wang W., Gao S., Shan X., Zhou J. (2023). Synergetic engineering of multiple pathways for *de novo* (*2S*)-naringenin biosynthesis in *Saccharomyces cerevisiae*. ACS Sustainable Chem Eng.

[14] Li Z., Gan Y., Gou C., Ye Q., Wu Y., Wu Y., Yang T., Fan B., Ji A., Shen Q., Duan L. (2025). Efficient biosynthesis of beta-caryophyllene in *Saccharomyces cerevisiae* by beta-caryophyllene synthase from *Artemisia argyi*. Synth Syst Biotechnol.

[15] Liu Q., Yu T., Li X., Chen Y., Campbell K., Nielsen J., Chen Y. (2019). Rewiring carbon metabolism in yeast for high level production of aromatic chemicals. Nat Commun.

[16] Pang X., Zhu J., Li Y., Xiao J., Zhang X., Ren D., Zhou P. (2025). Development of a highly efficient *p*-coumaric acid-responsive biosensor in *Saccharomyces cerevisiae*. Synth Syst Biotechnol.

[17] Zhou S., Lyu Y., Li H., Koffas M.A.G., Zhou J. (2019). Fine-tuning the (*2S*)-naringenin synthetic pathway using an iterative high-throughput balancing strategy. Biotechnol Bioeng.

[18] Zhang S., Liu J., Xiao Z., Tan X., Wang Y., Zhao Y., Jiang N., Shan Y. (2024). Systems metabolic engineering of *Saccharomyces cerevisiae* for the high-level production of (*2S*)-eriodictyol. J Fungi.

[19] Zhang S., Xiao F., Liu J., Xiao Z., Tan X., Wang Y., Zhao Y., Lu Q., Zuo S., Hu F., Jiang N., Lyu Y., Shan Y. (2025). Systematic engineering of eriodictyol-7-*O*-glucoside biosynthetic pathway through enzyme screening and gene regulation. J Agric Food Chem.

[20] Oka T., Nemoto T., Jigami Y. (2007). Functional analysis of *Arabidopsis thaliana* RHM2/MUM4, a multidomain protein involved in UDP-D-glucose to UDP-L-rhamnose conversion. J Biol Chem.

[21] Xiao Z., Wang Y., Liu J., Zhang S., Tan X., Zhao Y., Mao J., Jiang N., Zhou J., Shan Y. (2023). Systematic engineering of *Saccharomyces cerevisiae* chassis for efficient flavonoid-7-*O*-disaccharide biosynthesis. ACS Synth Biol.

[22] Liu M., Wu J., Yue M., Ning Y., Guan X., Gao S., Zhou J. (2024). YaliCMulti and YaliHMulti: stable, efficient multi-copy integration tools for engineering *Yarrowia lipolytica*. Metab Eng.

[23] Gao S., Xu X., Zeng W., Xu S., Lyv Y., Feng Y., Kai G., Zhou J., Chen J. (2020). Efficient biosynthesis of (*2S*)-eriodictyol from (*2S*)-naringenin in *Saccharomyces cerevisiae* through a combination of promoter adjustment and directed evolution. ACS Synth Biol.

[24] Feng Y., Yao M., Wang Y., Ding M., Zha J., Xiao W., Yuan Y. (2020). Advances in engineering UDP-sugar supply for recombinant biosynthesis of glycosides in microbes. Biotechnol Adv.

[25] Wang Y., Sun Q., Chi Y., Liu Z., Liu H., Li C., Feng X. (2023). Constructing an intensified UDP recycling system for the glycosylation of natural products by phosphorylation of byproduct fructose. J Agric Food Chem.

[26] Yang C., Tian F., Ma J., Chen M., Shi X., Chen D., Xie Y., Zhou X., Zhou Z., Dai X., Xia T., Gao L. (2023). Glycosylation of secondary metabolites: a multifunctional UDP-glycosyltransferase, CsUGT74Y1, promotes the growth of plants. J Agric Food Chem.

[27] Wu Q., Wei M., Feng L., Ding L., Wei W., Yang J., Lin X., Liang H., Zhan R., Ma D. (2022). Rhamnosyltransferases involved in the biosynthesis of flavone rutinosides in *Chrysanthemum* species. Plant Physiol.

[28] Ru Z., Liu M., Chen Q., Li H., Ning Y., Zeng W., Zhou J. (2025). High-level *de novo* production of (*2S*)-naringenin in *Yarrowia lipolytica* using metabolic and enzyme engineering. ACS Agric Sci Technol.

[29] Yue M., Liu M., Gao S., Ren X., Zhou S., Rao Y., Zhou J. (2024). High-level *de novo* production of (*2S*)-eriodictyol in *Yarrowia lipolytica* by metabolic pathway and NADPH regeneration engineering. J Agric Food Chem.

[30] Pei J., Chen A., Sun Q., Zhao L., Cao F., Tang F. (2018). Construction of a novel UDP-rhamnose regeneration system by a two-enzyme reaction system and application in glycosylation of flavonoid. Biochem Eng J.

[31] Yuan Z., Li G., Zhang H., Peng Z., Ding W., Wen H. (2024). Four novel Cit7GlcTs functional in flavonoid 7-*O*-glucoside biosynthesis are vital to flavonoid biosynthesis shunting in citrus. Hortic Res.

[32] Letunic I., Bork P. (2024). Interactive Tree of Life (iTOL) v6: recent updates to the phylogenetic tree display and annotation tool. Nucleic Acids Res.

[33] Shi Z., Wang D., Li Y., Deng R., Lin J., Liu C., Li H., Wang R., Zhao M., Mao Z., Yuan Q., Liao X., Ma H. (2024). REME: an integrated platform f or reaction enzyme mining and evaluation. Nucleic Acids Res.

[34] Wang Y., Huang R., Gao S., Yue M., Zhang X., Zeng W., Tang B., Zhou J., Huang D., Xu S., Wang R.H.Y., Gao S., Zeng W., Tang B., Zhou J., Huang D., Xu S. (2025). Identification of two new flavone 4'-*O*-methyltransferases and their application in *de novo* biosynthesis of (*2S*)-hesperetin in *Yarrowia lipolytica*. Synth Syst Biotechnol.

[35] Zhang P., Wei W., Shang Y., Ye B. (2023). Metabolic engineering of *Yarrowia lipolytica* for high-level production of scutellarin. Bioresour Technol.

[36] Wang Q., Zhao M., Zabed H.M., Tang X., Chen J., Wang J., Li H., Qi X. (2025). Data-driven mining of a thermostable lipase with molecular insights into mechanisms of its substrate specificity. J Agric Food Chem.

[37] Wang Z., Xie D., Wu D., Luo X., Wang S., Li Y., Yang Y., Li W., Zheng L. (2025). Robust enzyme discovery and engineering with deep learning using CataPro. Nat Commun.

[38] Maharjan R., Fukuda Y., Nakayama T., Nakayama T., Hamada H., Ozaki S.I., Inoue T. (2022). Structural basis for substrate recognition in the *Phytolacca americana* glycosyltransferase PaGT3. Acta Crystallogr D Struct Biol.

[39] Bkassiny S., N'Go I., Sevrain C.M., Tikad A., Vincent S.P. (2014). Synthesis of a novel UDP-carbasugar as UDP-galactopyranose mutase inhibitor. Org Lett.

[40] Seifert G.J. (2004). Nucleotide sugar interconversions and cell wall biosynthesis: how to bring the inside to the outside. Curr Opin Plant Biol.

[41] Yin S., Liu M., Kong J. (2016). Functional analyses of OcRhS1 and OcUER1 involved in UDP-L-rhamnose biosynthesis in *Ornithogalum caudatum*. Plant Physiol Biochem.

[42] Han X., Qian L., Zhang L., Liu X. (2015). Structural and biochemical insights into nucleotide-rhamnose synthase/epimerase-reductase from *Arabidopsis thaliana*. Biochim Biophys Acta.

[43] Smith M.R., Gao H., Prabhu P., Bugada L.F., Roth C., Mutukuri D., Yee C.M., Lee L., Ziff R.M., Lee J.K., Wen F. (2019). Elucidating structure-performance relationships in whole-cell cooperative enzyme catalysis. Nat Catal.

[44] Wang P., Wang J., Zhao G., Yan X., Zhou Z. (2021). Systematic optimization of the yeast cell factory for sustainable and high efficiency production of bioactive ginsenoside compound K. Synth Syst Biotechnol.

[45] Guo Q., Xu J., Li J., Tang S., Cheng Y., Gao B., Xiong L., Xiong J., Wang F., Wei D. (2024). Synergistic increase in coproporphyrin III biosynthesis by mitochondrial compartmentalization in engineered *Saccharomyces cerevisiae*. Synth Syst Biotechnol.

[46] Wang S., Zhu Q., Liu C., Dong H., Xia M., Jin Z., Zhang D. (2026). Engineering riboflavin-overproducing *Bacillus subtilis* via pathway gene overexpression. Synth Syst Biotechnol.

[47] Hu X., Yang P., Chai C., Liu J., Sun H., Wu Y., Zhang M., Zhang M., Liu X., Yu H. (2023). Structural and mechanistic insights into fungal *β*-1,3-glucan synthase FKS1. Nature.

[48] Rowen D.W., Meinke M., LaPorte D.C. (1992). GLC3 and GHA1 of *Saccharomyces cerevisiae* are allelic and encode the glycogen branching enzyme. Mol Cell Biol.

[49] Li Q., Liu W., Zhang Y., Jin J., Ji P., Yuan Z., Zhang Y., Feng P., Wu Y., Shen H., Wang P. (2025). ALG5 downregulation inhibits osteogenesis and promotes adipogenesis by regulating the N-glycosylation of SLC6A9 in osteoporosis. Cell Mol Life Sci.

[50] Zhu S., Li N., Liu Z., Yuan Y., Li B. (2025). Engineering budding yeast for the de novo synthesis of valuable flavanone derivatives. Green Chem.

[51] Wang J., Mao J., Yang G., Zheng F., Niu C., Li Y., Liu C., Li Q. (2018). The FKS family genes cause changes in cell wall morphology resulted in regulation of anti-autolytic ability in *Saccharomyces cerevisiae*. Bioresour Technol.

[52] Rodriguez L.J., Trilla J.A., Castro C., Valdivieso M.H., Durán A., Roncero C. (2000). Characterization of the chitin biosynthesis process as a compensatory mechanism in the fks1 mutant of *Saccharomyces cerevisiae*. FEBS Lett.

[53] Liu J., Xiao Z., Zhang S., Wang Z., Chen Y., Shan Y. (2023). Restricting promiscuity of plant flavonoid 3'-hydroxylase and 4'-*O*-methyltransferase improves the biosynthesis of (*2S*)-hesperetin in *E. coli*. J Agric Food Chem.

[54] He S., Zhang Z., Lu W. (2023). Natural promoters and promoter engineering strategies for metabolic regulation in *Saccharomyces cerevisiae*. J Ind Microbiol Biotechnol.

[55] Gao S., Zhou H., Zhou J., Chen J. (2020). Promoter-library-based pathway optimization for efficient (*2S*)-Naringenin production from *p*-Coumaric acid in *Saccharomyces cerevisiae*. J Agric Food Chem.

[56] Luelf U.J., Böhmer L.M., Li S., Urlacher V.B. (2023). Effect of chromosomal integration on catalytic performance of a multi-component P450 system in *Escherichia coli*. Biotechnol Bioeng.

[57] Huang Y., Zhong W., Varga K.E., Benko Z., Pocsi I., Yang C., Molnar I. (2025). Promoting the glycosylation of drug-like natural products in a *Saccharomyces cerevisiae* chassis by deletion of endogenous glycosidases. Bioresour Technol.

[58] Wang H., Yang Y., Lin L., Zhou W., Liu M., Cheng K., Wang W. (2016). Engineering *Saccharomyces cerevisiae* with the deletion of endogenous glucosidases for the production of flavonoid glucosides. Microb Cell Fact.

[59] Li S., Luo S., Zhao X., Gao S., Shan X., Lu J., Zhou J. (2024). Efficient conversion of stevioside to rebaudioside M in *Saccharomyces cerevisiae* by a engineering hydrolase system and prolonging the growth cycle. J Agric Food Chem.

