# Supplementary material for: De novo biosynthesis of eriocitrin in Saccharomyces cerevisiae through deep learning-guided enzyme screening and systematic metabolic engineering
Source: Synth Syst Biotechnol. 2026 Feb 4;13:143–53. doi: 10.1016/j.synbio.2026.01.019 (PMC12905779; doi:10.1016/j.synbio.2026.01.019)
Supplement: Multimedia component 1 [file mmc1.docx]

**Supporting Information**

## *De Novo* Biosynthesis of Eriocitrin in *Saccharomyces cerevisiae* through Deep Learning-Guided Enzyme Screening and Systematic Metabolic Engineering

Qiyuan Lu^a,b^, Xinjia Tan^a,b^, Siqi Zhang^a,b^, Yongtong Wang^a,b^, Yifei Zhao^a,b^, Juan Liu^b^, Fanglin Hu^a,b^, Shasha Zuo^a,b^, Jiaxu Chen^a,b^, Liusha Fan^a,b^, Shenghua Ding^b^, Zhiqiang Xiao^b⁎^, and Yang Shan^b⁎^

a Longping Agricultural College, Hunan University, Changsha 410125, China.

b DongTing Laboratory, Hunan Institute of Agricultural Product Processing and Quality Safety, Hunan Academy of Agricultural Sciences, Changsha 410125, China.

*Corresponding author: Zhiqiang Xiao, and Yang Shan

E-mail addresses: [xiaozhiqiang@hnu.edu.cn](mailto:xiaozhiqiang@hnu.edu.cn) (Zhiqiang Xiao), and 1057771365@qq.com (Yang Shan)

### Table S1

**Table S1.** Genes used in this study.

| Gene | Original organisms | Sources |
| --- | --- | --- |
| *ApGT* | *Andrographis paniculata* | MH379335 |
| *AtGT* | *Arabidopsis thaliana* | AY090273 |
| *BvGT* | *Beta vulgaris* | AY526081 |
| *CjGT* | *Citrus japonica* | MN393519 |
| *CsGT* | *Camellia sinensis* | KP682364 |
| *MdGT* | *Malus x domestica* | AY786997 |
| *SlGT* | *Scutellaria laeteviolacea* | AB362989 |
| *StGT* | *Solanum tuberosum* | MF134427 |
| *VlGT* | *Vitis labrusca* | EF533705 |
| *OlRHM* | *Ornithogalum longebracteatum* | KX289608.1 |
| *AtRHM1* | *Arabidopsis thaliana* | NM_106504.4 |
| *VvRHM* | *Vitis vinifera* | XM_002285598.3 |
| *AtNRS/ER* | *Arabidopsis thaliana* | NM_104978.5 |
| *Cs1,6-RhaT* | *Citrus sinensis* | NM_001288900.1 |
| *Ci1,6-RhaT* | *Chrysanthemum indicum* | OL422134 |
| *Cm1,6-RhaT* | *Citrus maxima* | LC057678 |
| *Cc1,6-RhaT* | *Citrus x clementina* | XP_006421028.1 |
| *AtCPR* | *Arabidopsis thaliana* | NP_001328167.1 |
| *CrCPR* | *Catharanthus roseus* | X69791.1 |
| *SmCPR* | *Silybum marianum* | [1] |
| *GhF3'H* | *Gerbera hybrid* | DQ218417.1 |
| *SmF3'H* | *Silybum marianum* | [1] |
| *ThF3'H* | *Tricyrtis hirta* | AB480691.1 |

### Table S2

**Table S2.** Codon-optimized exogenous gene sequences involved in this study.

| Gene | | | Sequence (5'-3') |  |
| --- | --- | --- | --- | --- |
| *ApGT* | | ATGGGCTACCATAGCCACATCGGTGTTCTGGCGTTCCCGTTCGGCTCTCACGCGGCGCCGCTGCTGGCGCTGGTTCGTCAGCTGGCGGAAAGCAGCCCAGGTACCTGCTTCTCTTTCTTCAACACCGCTGATTGCAACCGTGCGATCCTGGCTGACCACGTGAGCCCGAACATCAAAGCGTATGATGTTCGTGATGGTGCACCGGAAGGTGCGGCAGCTGCAGCAAGCCATATGGAAGCGATCCGTCTGTTCCTGGCTGCTTCTCCGGGCAACTTCGAAGCAGGTATGGAAGAAGCAGAACGTGCGGCGGGTGTTGGCATCGGTTGCCTGATCACCGATGCGTTCCTGTGGTTCGCTGCTGATCTGGCGGCTAACCGCCGTATCCCGTGGGTTGCTCTGTGGACCGCAGCGGCGTGTGCGCTGGCTACCCACATGTATACCCAGGAAATCGTTAAAGCTGTTGCTAGCCCGGCTGAAGCAACCGGTGAAAGCATCCAGCAGGTTCTGTCTTTCACTCCGCCGGGTCTGCCGCCGCTGCAGATTGCGGATCTGCAGCCGGAAATCTTTATCGATGACCGTAACCCGAGCCCGCTGGCAGCTACCATCAACAACATGGTTGAAAAACTGCCGAAAAGCACCGCGGTGGTTCTGAACTCCTTCGAAGAAATCGATCCGATCGTGGCGCAGGACCTGAAAAGCAAATTCAACCACTTCCTGAGCGTTGGTCCGGCAGCACTGCGTCGCCTGCCGCAGCCGCCGCCGGGTGACGATTCCGGTTGCCTGTCTTGGCTGGAAAAACTGCCGACCCCGCGTTCCGCTATCTATGTGTCCTTCGGCACTGTTATCGTTCCGCCGGAAAACGAACTGCTGGCTCTGGCGGAAGCACTGGAATTTTGCAAATTCCCGTTCCTGTGGTCTCTGAAAGAAGCCGCTGCAAAAGCGCTGCCGGAAGGCTTCCTGCGTCGCACCGCGGAATACGGTCGTGTTGTTCCGTGGGCGCCGCAGCCGTGGATTCTGCGTCACGGCAGCGTGGGTCTGTTCGTTACTCACGGTGGTTGGAACTCTATCCTGGAATCCATTTGCGGCGGTGTACCGATGGTTTGCCGTCCGTTCTTTGGCGATCAGAAACTGAACGGTAAAATGGTTGAAGATTCTTGGAAAATCGGCGTTCGTGTTCGTGATGGCGTGTTTCGTAAAGATGAAACTGTTCGCGTTCTGCAGCGTATGATGAGCTCTCGTGAAGGTGATGATGCTCGCGAAAACGTTGTTAAAATGAAAGAAAAAGCGGAAAAAGCGGTTGGTAACGATGGTTCTTCTACCAAAAACTTCAAAAAACTGCTGGAAATCATCGGCATCTCTAAATAA | |  |
| *AtGT* | | ATGGGCACCCCGGTTGAAGTTAGCAAACTGCACTTCCTGCTGTTCCCGTTCATGGCGCACGGCCACATGATCCCGACCCTGGATATGGCAAAACTGTTCGCGACCAAAGGTGCGAAAAGCACCATTCTGACCACCCCGCTGAACGCTAAACTGTTCTTTGAAAAACCGATCAAAAGCTTCAACCAGGATAATCCGGGCCTGGAAGATATCACCATTCAGATCCTGAACTTCCCGTGCACCGAACTGGGTCTGCCGGATGGTTGCGAAAACACCGATTTCATTTTCAGCACCCCGGATCTGAACGTAGGTGATCTGTCCCAGAAATTTCTGCTGGCGATGAAATACTTCGAAGAACCGCTGGAAGAACTGCTGGTTACCATGCGCCCGGATTGCCTGGTTGGTAACATGTTCTTCCCGTGGTCTACCAAAGTTGCGGAAAAATTCGGTGTGCCGCGTCTGGTGTTCCACGGCACCGGTTACTTCAGCCTGTGCGCGTCCCACTGCATTCGTCTGCCGAAAAACGTAGCGACCAGCTCTGAACCGTTCGTGATCCCGGATCTGCCGGGTGACATCCTGATTACTGAAGAACAGGTGATGGAAACCGAAGAAGAAAGCGTGATGGGTCGTTTCATGAAAGCGATCCGTGATTCTGAACGTGACAGCTTCGGTGTTCTGGTTAACTCTTTCTACGAACTGGAACAGGCGTATAGCGACTATTTCAAAAGCTTCGTTGCGAAACGTGCATGGCACATCGGTCCGCTGAGCCTGGGTAACCGCAAATTTGAAGAAAAAGCGGAACGTGGCAAAAAAGCGTCCATCGATGAACACGAATGTCTGAAATGGCTGGATTCCAAAAAATGCGACAGCGTGATCTACATGGCTTTCGGCACCATGTCCTCTTTCAAAAACGAACAGCTGATCGAAATCGCAGCCGGCCTGGATATGTCTGGCCATGACTTCGTTTGGGTGGTTAACCGCAAAGGTTCTCAGGTTGAAAAAGAAGATTGGCTGCCGGAAGGCTTCGAAGAAAAAACCAAAGGCAAAGGCCTGATCATCCGTGGCTGGGCGCCGCAGGTTCTGATTCTGGAACACAAAGCTATCGGCGGTTTCCTGACCCACTGCGGCTGGAACAGCCTGCTGGAAGGTGTTGCCGCGGGCCTGCCGATGGTAACCTGGCCGGTTGGTGCGGAACAGTTCTACAACGAAAAACTGGTTACCCAGGTTCTGAAAACCGGTGTTAGCGTTGGCGTTAAAAAGATGATGCAGGTTGTTGGCGATTTCATTTCTCGTGAAAAAGTGGAAGGCGCGGTTCGTGAAGTTATGGTTGGTGAAGAACGTCGTAAACGCGCGAAAGAACTGGCTGAAATGGCGAAAAACGCAGTTAAAGAAGGTGGCTCTTCTGATCTGGAAGTTGATCGTCTGATGGAAGAACTGACCCTGGTTAAACTGCAGAAAGAAAAAGTTTAA | |  |
| *BvGT* | | atggatgataagtctcaacagcttcatattgttctttttccttttatggctcatggacatatgattccaactcttgatattgctagattgtttgctgctagaggagttaagacaacattgattacaactcctagaaatgctcctactttcttgactgctattgaaaaaggtaataagtctggtgctcccactattaatgttgaagtttttaatttccaggctcaatcttttggacttcctgaaggttgtgaaaatcttgaacaagctttgggacctggaattagagatagattctttaaggctgctgctatgcttagagatcaattggaacattttcttgaaaaaactagacctaactgtcttgttgctgatatgtttttcccttgggctactgattctgctgctaagttcaatatccctagattggtttttcatggtcattgtctttttgctctttgtgctcttgaaattattagattgcatgaaccttataataatgcttcttctgatgaagagcctttccttttgcctcatttgccacatgaaattgaacttactagattgcaattttctgaagaactttggaagaatggtggagattcagattataaagaaagatctaaagctattaaggaatctgaacttaagtgttacggagttcttgttaattctttttatgaattagagcctgattacgctgaatattttagaaaagatttgggtagaagagcttggaatattggtcctgttagtctttataatagatctaatgaagaaaaggctcaaagaggtaaacaagcttctattgacgagcatgaatgtttgaaatggttgaattctaagaaacctaattctgttatctatatttgtttcggttctactatgcatatgattccttctcaattgaatgagattgctatgggtcttgaagctagtggtaaagattttatttgggttgttagaaatgaggacgatctgggagagttcgagcaacgcatggaaggaaaaggccttatcattcgaggttgggctccacaggttcttatactggagcacgaagtcatcggggctttcgtcactcactgcggctggaattccacgatcgagggtatagctgcaggcgttccaatggttacttggccagtgttcgctgagcagttcttgaacgagaaactgattactcgagtgctgcgtatcggaataccagtgggcgccaagaagtgggattgtaagccgtcagaggagtacgttgtgaaaaagaacgatatcgaaaaggctttgagagaggtcatggaggggaatgaggctgaggaacgtagaacgcgggctaaggaatataaggagatggcttggaaagccttacaagagggaggttctagttacagtgacctttcagctctaattgatgaattgagaggtctttctacttga | |  |
| *CjGT* | | ATGACCATGCGTAAACTGAACCTGGTTTTCACCTCTACCCCAGGTATCGGTAACCTGGTTCCGGTTGTTGAATTTGCGCGTCTGCTGACCAACCGTGATCGTCGTTTCTCTGCGACCGTGCTGATCATCACCATCCCGGAACGTCCGATTGTTAACTCTTACATCCAGACCCGTGGTACCGCGCTGTCCGTTCACGATAACGATGATGTTAACTTCCTGCACCTGCCGACCGTTGATCCGCTGAGCCCGGATGAATACCAGAGCAGCCTGGGCTACCTGTGCACCCTGATCGAAAAACACAAACCGCACGTAAAACACGCTATCGCAAACCTGATGGCGACCGAAAGCGGTTCTGATAACGCGGTTTCTGTGCGTGTTGCTGGTCTGTTCGTTGACATGTTCTGCACTTCTATGATTGATGTGGCGAACGAACTGGGCATTCCGTCTTACCTGTACTTCGCGAGCCCGGCTTCTTTCTTGGGTTTTCTGCTGTACTTCCCGACCCTGGACGCGCAGCTGGCAACCGAATTTGTTGATTCTGATACCGAGTTCATCGTGCCGAAAGACAGCTCTATCACTGAACTGAAAATCCCGTCTTTCGCTAACCCGCTGCCGCCGCTGGTGCTGCCAACCACCGCGCTGAAACGCAAACAGGATGGCTATATGTGGTATCTGTATCATGGTCGCCGTTACCTGGAAACCAAAGGCATGATTGTGAACACCTTCCAGGAACTGGAGCCGTACGCGATTGATAGCCTGCGCGTTACCGAAATGCCCCCGGTTTACCCGATTGGTCCGGTTCTGGATCTGCATGGCCTGGCGCAGTGGCACCCGGACCGTGCATCCCAGGAAAAAATTATGCGTTGGCTGGACGATCAGCCGCCATCTTCTGTGGTTTTCCTGTGCTTCGGTAGCATGGGCAGCCTGTCTGAAGCACAGCTGCGTGAAATCGCGGTAGGCCTGGAACGCACCGGCTTCCGTTTCCTGTGGAGCATCCGTGAACCGAGCAAAGGCACCATCTATCTGCCGGGTGAATACACCAACCTGGAAGAAATCCTGCCGGAAGGTTTCTTCCACCGTACCGCCAAAATCGGTCTGGTTTGCGGTTGGGTTCCGCAGGTTACCATCCTAGCGCACCAGGCGGTGGGTGGCTTTGTTAGCCACTGCGGCTGGAACTCTATCCTGGAATCCCTGTGGTTCGGCGTTCCGATGGCGACCTGGCCGGTATACGCAGAACAGCAGATGAACGCGTTCCAGCTGGTTAAAGAATTTGGCCTGGCGGTTGAAATCCGTCTGGACTACCGTGAAGGCTCTGACGTTGTGCTGGCTGAAGAACTGGAAAAAGGTCTGCAGCAGCTGATGGATGGTGATGATGAAGTGCGTCGTAAAGTTAAACAGATGAAAGAAAAAAGCTGGACCGCAATGATGGAAGATGGCAGCAGCTATAAATCTCTGGGTTCTCTGATCGAAGAACTGATGGCTAACATCGGCTGCTAA | |  |
| *CsGT* | | ATGGTTCAGCACGGCCACATCCTGCTGCTGACCTTCCCGGCGCAGGGTCACATCAACCCGAGCCTGCAGTTCGCTAAACGTCTGATTAACATGGGTCTGCAGGTTACCTTCGCGACTAGCGTTTTCGCGCAGCGTCGTATCAGCAAAACCACCGGCACCACCGCGAAAGGCCTGAAATTCGCGGCTTTCAGCGATGGTTACGATGACGGTTTCCAGCCGGGTAACGATGTGCAGCACAAATTTAGCGAAATCCGTATCAACAGCAGCCTGGCAATCCGTGAAATTATCGCAGCGTCCGCGGCAGAAGGCCGTCCGGTGACCTGCCTGGTTTATACTCTGCTGCTGCCGTGGGCGGCTAAAGTTGCACGTGACTGCCACATCCCGAGCGCCCTGCTGTGGATTCAGCCGGCGACCGTTCTGGATATCTACTATTACTATTTCAACGGTTACAAAGAAGTTATCACCAAAAACTGCAACGGTAAAGACTCTTCTAGCAGCTGCAGCATCGAACTGCCGGGTCTGCCGCTGCTGACTAGCCACGATCTGCCGTCTTTCCTGTTCAGCAGCTCTTCCGATATCTACAGCCTGAGCCTGCCGACCTTCAAAGAACACATTGAAACCCTGGATGCTGAAACCTCCCCGAAAGTTCTGGTTAACACCTTCGATGCGCTGGAACCGGAAGGCCTGAAAGCGATCGGTAAATACAACCTGATCGGTATCGGCCCGCTGATCCCGAGCGTTTTCCTGGATGGTAAAGATCCGAGCGACACCAGCTTCAAAGGTGACCTGTTCCACGCATCCGGTAACTACATTGAATGGCTGAGCAGCAAACCGAAATCTTCTGTTGTTTACGTTTCCTTCGGTTCTCTGCTGGTTCTGCCGATGCCGAAACGTCAGATGGAAGAAATTGGCCGCGGTCTGCTGGAATCTCACCGCCCGTTTCTGTGGGTTATGCGTGGTGAAGAAGAAAAAGTTGAAGAAGATCGCCTGTCCTGCATTGAAGAACTGAAACAGCAGGGCATGATCGTGCCGTGGTGCAGCCAGCTGGAAGTTCTGAGCCACCCGAGCCTGGGTTGCTTCGTAACCCACTGCGGTTGGAACTCCACCCTGGAATCCTTTGCGTCCGGTGTTCCGATGGTTGCGTTCCCGCAGTGGACCGATCAGTGGACCAACGCGAAACTGGTTGAAGATGTTTGGAAAACCGGTGTTCGTGTTCGTCGTAACGAAGAAGGTATTGTTGAAGGTGATGAAATTAAACGTTGCATTGAAATGGTTATGGAAGATGGTGTTCGTGGTGAACAGATGCGTCGTAACGCGAAAAAATGGGGTGATCTGGGCCGTGAAGCTGTTAAAAAAGCGGGCTCTTCTAACAAAAACCTGATGAGCTTCGTTGAAGAAGTTGGTGGTGATTGCCTGTAA | |  |
| *MdGT* | | atggttcaacatagatttttgttagtaacatttccagctcaaggtcacattaatccttctcttcaatttgctaaaagattgattaatactactggtgctcatgttacatacgtaactagtttgtctgctcatagacgtattggtaatggatctattcctgatggattgacttatgctccattttctgatggttatgatgatggttttaaaccaggagataatgttgatgattacatgtctgaacttagaagaagaggtgttcaagctattactgatcttgttgttgctagcgctaatgaaggtcatccttacacttgtttagtttattctttgcttttaccatggtctgctggaatggctcatgaattgcatttgccttctgttcttttatggattcaacctgctacagtttttgatatatactactattactttaatggttataaggatttgattagagataatactagtagtggaactaataatgttctaccttgttctattgaattgcctggattgcctttatctttcactagtagagatttgccatcttttatggttgatactaatccatataattttgcacttcctttatttcaagaacaaatggaattattagaaagagaaactaatcctactattcttgtaaacacatttgatgctctagaacctgaagctcttaaagctattgataaatataatttgattggtgttggacctttgattccttcagcatttcttgatggtaaagatccttctgataagtcatttggtggagacttatttcaaaaatcaaaggattctagttatcttgaatggttaaattctaaacctgaaggttcagttatctatgtttcttttggttctatttctgttttaggaaaggcccaaatggaagaaatagctaaaggtttgcttgattgcggtttaccatttttatgggtaattagagataaagtaggtaaaaaaggagatgataatgaagctaagaaagaagaagaaatgttgagatgtagagaagaattagaagaattgggaatgattgttccttggtgttctcaggttgaagttcttagtagtccttctttaggatgttttgttacacactgtggttggaatagttcacttgaatctttagtatctggtgttcctgttgttgcatttccacaatggactgatcagggtactaatgctaaactaattgaagattattggaaaacaggtgttagagtaactcctaatgaagaaggaattgttacaggtgaggaattgaagagatgtttagatttggttttaggtagtggtgaaataggtgaagatgttagaagaaatgctaagaaatggaaggatcttgctagagaagcagttagtgaaggagattcttctgataagaatttgagagcttttcttgatcaaattaaggttcttaaggatgctagacattga | |  |
| *SlGT* | | ATGGAAGATACCATCGTGATCTACACCACCCCGGAACACCTGAACACCATGGCTGTTCTGGCTAAATTCATCAGCAAACACCACCCGTCTGTTCCGATCATCCTGATCTCTACCGCGGCGGAATCCGCAGCGGCGTCTATTGCAGCGGTTCCGTCTATCACCTACCACCGTCTGCCGCTGCCGGAAATCCCGCCGAGCCTGACCAAAGATCGTGTTGAACTGTTCTTCGAACTGCCGCGTCTGTCTAACCCGAACCTGCGTCTGGCTCTGCAGGAAATCTCCCAGAAAGCTCGTATCCGTGCTTTCGTTATCGACTTCTTCTGCAACGCTGCATTCGAAGTTAGCACCTCCCTGAGCATCCCGACCTTCTACTACTTTAGCTCTGGTAGCCCGACCGCGACCCTGGTGCTGCACTTTCAGACCCTGGACGAAACCATTCCGGGTGATCTGAAAGACCTGGATGATTTCGTTGAAATTCCGGGCCTGCCGCCGATCTATTCCCTGGATATCCCGGTTGCTCTGCTGACCCGCCAGAGCCTGGTTTATCAGTCTAGCGTTGATATCAGCAAAAACCTGCGTAAATCCGCAGGTTTCCTGGTTAACGGTTTCGATGCACTGGAATTTCGTGCGAAAGAAGCGATCGTGAACGGTCTGTGCGTGCCGAACGGTCCGACCCCGCCGGTTTACTTTATCGGTCCGCTTGTTGGTGATGTGGATGCAAAAGCGGGCGGTGAAGAACACGAATGCCTGCGTTGGCTGGATACCCAGCCGAGCAAAAGCGTTATCTTCCTGTGCTTCGGTCGCCGTGGCGTTTTCTCTGCTGAACAGCTGAAAGAAACCGCGGTTGCGCTGGAAAACTCTGGTCATCGTTTCCTGTGGAGCGTTCGTAACCCGCCGGAAATCATGAAAAACTCTGATGAACCTGATCTGGATGAACTGCTGCCGGAAGGTTTCCTGGAACGTACCAAAGATCGTGGTTTCGTTATCAAATCTTGGGCGCCGCAGAAAGAAGTTCTGAGCCACGATAGCGTGGGTGGCTTCGTTACCCACTGCGGTCGTTCTTCCATCTCCGAAGGTGTTTGGTTCGGCGTGCCGATGATCGGTTGGCCGGTGGATGCAGAACAGAAACTGAACCGTACCGTGCTGGTTGAAGAAATGCAGGTTGCGCTGCCGATGGAAGAAGCTGAAGGTGGCTTCGTTACCGCGGCTGAATTAGAAAAACGTGTTCGCGAACTGATGGAATCTAAAGTTGGTAAAGCAGTTCGCCAGCGCGTTGGTGAACTGAAATGCTCTGCGCGCGCGGCGGTTACCGGTAACGGTAGCAGCCTGTCTGATTTCAAAAAATTCCTGCTGGCGACCCGTGATTAA | |  |
| *StGT* | | atggctatggaacaaaatgaagaaactgctatgcctcatgttgtttttattccttatgctatgacttctcatattacgcctcttgttcatattgctagactttttgctcttcatggattgaaggttactattattgctcctcaacataatgctcttttgttccaatcttctgttgatagagatagattgttttctggttctaatattactgttagaactattcagtttccatctgaagaagtgggacttcctgttggtattgaaaattttattgcttctccatctatggaaattgttggtaaagttcattatggttttattttattgcaaaagattatggaacaacttattagagagattaatccaaattgtattgtttctgatatgttctttccttggactgttgatcttgctgaggaaatgcaaattcctagattttctttccaacctgctacttctattcatcaatgtgcttgggtttttattagagaattcaaaccttataagaatgttgctagtgatgctgaaaagtttttgattcctggacttcctctcgatattaagatgaaagtttctgaaattgaagattttcttaaagaggaaactgaatatactaagactgttgatgatgttcttcaagctgaagttagatctcatggaattattcataatacttgttctgaattggaacctggtgttgctcaactttatgaaaaggctagaggagttaagggttggcatattggtccattggctttgttcattaataagtatgaagctgaaatttcttctaagcaaatttctaactctaacatcaactcctgctctgatccctggaagggatacggagattgctttaactggctcgagaaccagcagcctaacagcgtgctcttcgtttgctttgggtctatgattagattctccgatgatcagctcaaggaaatggccgtgggcctcaaggctgccaactgtcctaccatctgggtgttcagggagcaggataagaatgaggttgatgagaaggatgagcactcggactggtctcgtaacggtttcaaggagatgatcggggagaagatgttcatcattcagggttgggctcctcagcagttgatcttgaagcatcaggctataggtggatttttaactcattgcggatggaatagtattttagaatctttagctgtaggagttccattaataacttggccattgtttagtgataatttctatacagataaattacttgaaacgttaggattagctattggtattggtgctgatgtttggaatcctggttttattctttcatgtcctcctctttctggcgaaaagattgaattggctgtgaaaagactcatgaataatagtgaagaatcaagaaagattagagaaaacgctaaattgatggctaaaaagttgaaatctgctactgaagaaggtggttcttctcatagtcaattaataggtttgattgaagaaattaagagatgtgcttttaagaagtctttttga | |  |
| *VlGT* | | ATGGATAAACACCACTTCCTGCTGCTGAGCTGCCCGGCGCAGGGCCACATCAACCCGACCCTGCACCTGGCTAAACTGCTGCTGCGTCTGGGTGTTCGTGTTACCTTCGCGACCTTCGTTAGCGGTCTGCGTCGCATTGCGACCCTGCCGACCATTCCGGGCCTGCACTTCGCTAGCTTTAGCGATGGCTACGATGATGGCAACAACAGCAACTATAGCATGGAAGAAATGAAACGTGTTGGTTCCCAGAGCCTGTCTAACCTGCTGCTGTCTCTGAGCAACGAACGTGGTCCGGTTACTTACCTGATCTACGGTTTCCTGCTGCCGTGGGCGGCGACTGTTGCGCGTGAACACGGTATCCCGTCCGCGTTCCTGTCTACCCAGAGCGCAACCGCCATCGCGGTTTACCACCGTTATTTCAAAGCGCACGATGGTCTGTTCAACACCGAACTGGGCAACAGCCTGAACATCAGCCTGGAACTGCCGGGTCTGCCGCCGCTGAAATACGAAGATCTGCCGAGCATTCTGCTGCCGACCTCTCCGCACGCGTGGGTTGTTCCGTCTTTCCAGGAACTGATCCAGAACCTGGAACAAGATCCGAACCCGTGCGTTCTGATCAACACCTTCAACGCACTGGAAGAAGATGTTATTAAAGCATTAGGCGATTTCATGAACGTGGTTGCAATTGGTCCGCTGATGCAGCTGGATTCTTCTATCAGCTGCGACCTGTTCGGTCGTAGCAAAGACTATCACCCGTGGCTGAACAGCAAACCGGAAGGTAGCGTTATCTACGTGTCTTTCGGCAGCCTGGCGACCCTGCAGAAAAAACAGATGGAAGAAATCTTCCACGGTCTGATGGAAAGCCACCGTCCGTTCCTGTGGGTTATTCGTTCCATGGAATCCGAACTGGAAGAAAAAATGAACTCTAGCCTGAGCGAAGAACAGGGCCTGATCGTTCAGTGGTGCTCCCAGGTTGAAGTGCTGTGCCACCAGGCGGTTGGCTGCTTTCTGACCCATTGCGGCTGGAACTCTACCATGGAATCTCTGGTTGCGGGTGTGCCGGTGGTTGCTTGCCCGCAGTTCAGCGACCAGACCACCAACGCAAAACTGGTTGAAGTTTGGGGTACCGGTGTGAAAGCTCGTGCGAACGAAGAAGGCGTTGTTGAACGTGAAGAAATCAAAAAATGCCTGGAAATGGTTATGGAAGGTGGCGAAAAAGGCGACGAAATGCGTCGCAACGCTAACAAATGGAAAGGCCTGGCCGTTGAATCTATGGAATACGGCTCCAGCGGCGAAACCAACCTGAAACACTTCGTTGAATCTCTGGAAGTTCGTACCCACTAA | |  |
| *AtRHM1* | | ATGGCTTCCTACACTCCAAAGAATATTTTGATTACTGGTGCTGCTGGTTTCATTGCTTCTCACGTTGCTAATAGACTAATTAGATCCTACCCAGATTACAAGATAGTCGTTTTGGATAAATTAGATTACTGTTCTAACTTGAAGAACTTGAACCCATCTAAGCACAGCCCAAATTTTAAGTTCGTTAAGGGTGACATTGCCTCTGCTGATTTGGTTAATCACTTGTTAATTACCGAAGGTATCGACACTATTATGCATTTCGCTGCTCAAACTCACGTTGATAACTCTTTTGGTAATTCTTTTGAATTCACCAAGAATAATATATATGGTACTCATGTTTTGTTGGAAGCATGTAAGGTCACTGGTCAAATTAGAAGATTCATTCACGTTTCCACCGATGAAGTTTACGGTGAAACCGATGAAGATGCTTTGGTTGGTAACCACGAAGCTTCCCAATTGTTACCTACTAACCCATATTCTGCAACTAAAGCTGGTGCTGAAATGTTGGTTATGGCATACGGTAGAAGCTATGGTTTGCCAGTGATTACAACTAGAGGTAATAACGTTTACGGTCCAAACCAATTCCCTGAAAAGTTGATTCCTAAGTTCATCTTGTTGGCCATGAGAGGTCAAGTTTTACCAATCCATGGCGATGGTTCTAACGTCAGATCTTACTTATACTGTGAAGATGTTGCTGAAGCTTTTGAAGTCGTTTTGCATAAAGGTGAAGTTGGTCACGTTTATAATATTGGTACTAAGAAAGAAAGAAGAGTTAATGACGTTGCTAAAGATATTTGTAAATTGTTCAACATGGATCCAGAAGCTAATATCAAGTTTGTAGATAACCGTCCTTTTAATGATCAAAGATACTTTTTGGACGACCAAAAGTTGAAAAAGTTAGGTTGGTCTGAACGTACTACTTGGGAAGAAGGTTTGAAGAAAACTATGGATTGGTATACTCAAAACCCAGAATGGTGGGGCGATGTCTCTGGCGCTTTATTGCCACATCCTAGAATGTTGATGATGCCAGGTGGTCGTCATTTCGACGGTTCCGAAGATAATTCTTTGGCCGCTACTTTGTCCGAAAAGCCATCTCAAACTCATATGGTTGTTCCTTCTCAAAGATCTAACGGTACCCCACAAAAGCCATCTTTGAAGTTTTTGATCTACGGAAAAACCGGTTGGATCGGTGGTCTATTAGGTAAAATTTGTGATAAGCAAGGTATTGCGTACGAATACGGTAAAGGTAGATTAGAGGACAGATCTTCTTTGTTGCAAGACATTCAGAGTGTTAAACCTACTCACGTTTTTAACTCTGCTGGTGTGACTGGTAGACCTAATGTTGATTGGTGTGAATCCCATAAGACAGAAACTATTAGAGCTAACGTTGCTGGTACTTTGACATTAGCTGATGTTTGTAGAGAACACGGCTTATTAATGATGAATTTCGCTACTGGTTGTATTTTTGAATACGATGACAAACACCCTGAAGGTTCTGGTATTGGTTTTAAAGAAGAAGATACACCTAACTTCACTGGTTCCTTTTACTCAAAAACTAAAGCCATGGTTGAAGAATTGTTGAAAGAATATGATAATGTTTGTACTTTAAGAGTTAGAATGCCTATTTCTTCTGATTTGAATAATCCTAGAAACTTTATTACTAAAATTTCAAGATACAATAAGGTTGTTAACATTCCAAACTCTATGACTGTTTTGGATGAATTATTACCAATTTCTATCGAAATGGCTAAGAGAAATTTGAAAGGTATTTGGAACTTCACTAACCCAGGTGTTGTTTCTCATAATGAAATTTTAGAAATGTACCGTGATTACATTAACCCAGAATTCAAGTGGGCTAATTTCACTTTAGAAGAACAAGCTAAAGTTATCGTCGCCCCTAGATCTAACAACGAAATGGATGCTTCTAAGTTGAAGAAAGAATTCCCAGAATTATTGTCTATTAAGGAATCTTTGATTAAGTACGCTTACGGTCCAAACAAGAAAACCTAA | |  |
| *OlRHM* | | ATGGCTTCTCATACCCCAAAGAACATTTTGATTACCGGTGCCGCAGGTTTCATTGCTTCTCATGTTGCTAATAGATTGGTTAGAAAGTATCCACAATACAAAATTGTTGTTTTGGATAAATTAGATTACTGTTCTAACTTGAAGAACTTGAGACCTTCACAATTGTCCCCAAACTTTAAGTTCGTTAAAGGTGACATCGCTTCAGCCGACTTAGTTAATTATTTGTTGATTACTGAATCTATTGACACTATTATGCATTTCGCTGCTCAAACTCACGTTGACAACAGTTTCGGTAATTCTTTTGAATTCACAAAAAATAATATCTACGGTACTCACGTTTTGTTGGAAGCTTGTAAAGTTACTGGTCAAATTAAGCGTTTCATCCATGTTTCTACTGATGAAGTCTACGGTGAAACCGATGAGGACGCCGTAGTCGGTAACCACGAAGCTTCCCAATTGTTACCAACTAACCCATACTCTGCTACTAAGGCTGGTGCTGAAATGTTAGTTATGGCTTACGGTAGATCTTATGGTTTGCCAGTTATTACTACCAGAGGTAATAATGTTTATGGTCCAAACCAATTTCCTGAAAAGTTGATTCCAAAATTCATTTTATTAGCTATGAGAGGTAAATCTTTGCCAATTCATGGTGACGGTTCTAACGTCCGTTCCTACTTATATTGTGAAGATGTTGCTGAAGCTTTCGAATTAATTTTACATAAAGGTGAAGTTGGTCATGTTTATAACATTGGTACCAAAAAAGAAAGAAGAGTCATTGATGTAGCTAAAGACGTTTGTAAGTTATTCTCTTTGGATGCTGATTCCGTTATCAAATTCGTTGAAAATAGACCTTTCAATGATCAAAGATATTTCTTGGATGACCAAAAATTGACTAACTTGGGTTGGTCTGAAAGAACCACTTGGGAAGAAGGTTTGAGAAAGACTATGGAATGGTATACTTCTAATCCAGAATGGTGGGGAGATGTTTCTGGTGCCTTATTGCCTCACCCTCGTATGTTAATGATGCCTGGTATTGAAAAACAATTTGATGGCCCAGCTGATATTAACGGCACTTTGTCTGAATTAATGAAAAAGCCAACACAAACTGAAAAAGAAGTTGAAGCTTCTAAGAGAACCGCTAACTCACCACAAAAGCCAATGTTGAAATTTTTGATCTACGGTAGAACTGGTTGGATTGGTGGCTTACTTGGTCAAATTTGTGAAAAGCAAGGTATTCCATACGAATATGGTAAAGGTAGATTACAAGAAAGATCTCAATTAGTTAGTGATATTCAATCTGTTAAGCCAACTCATGTTTTTAACGCAGCAGGTGTGACTGGTAGACCAAATGTCGATTGGTGTGAATCCCACAAGCCAGAAACTATCAGAACTAATGTTGTTGGTACTTTGACATTAGCTGATGTCTGTAGAGAACACGGTTTGTTAATGATGAATTACGCTACTGGTTGTATATTCGAATACGACGCTCAACATCCAGAAGGTTCTGGTGTTGGTTTCAAAGAAGAAGATACACCAAATTTCGCTGGTTCTTTCTACTCCAAAACTAAGGCAATGGTCGAAGAATTGCTAAAGGACTATGATAACGTTTGTACTTTGAGAGTTAGAATGCCAATCTCCTCTGATTTGTCTAACCCTAGAAACTTTATTACTAAGATTTCTAGATACAACAAAGTTGTTAACATTCCAAACTCTATGACTGTTTTGGATGAATTGTTACCAATTTCAGTCGAAATGGCTAAGAGAAACTTGAGAGGTATCTGGAACTTCACTAATCCAGGTGTTGTTTCCCATAATGAAATCTTGGAAATGTATAAGAAGTACATGGATCCAGGTTTTAAGTGGTGTAATTTCACTTTGGAAGAACAAGCTAAGGTTATTGTTGCTGCTAGAAGTAATAACGAAATGGATGCCTCAAAGTTGAAAAAAGAATTCCCTGAATTGTTATCTATTAAGGACTCCTTGATTAAGTATGTTTTTGAACCAAATAAGAAGGTTTAA | |  |
| *VvRHM* | | ATGGCTACCCACACTCCAAAGAATATTTTGATTACTGGTGCCGCCGGTTTCATTGCTTCTCATGTTGCTAATAGATTGATTAGAAACTACCCTGATTACAAAATCGTTGTTCTAGATAAGTTGGATTACTGTTCTAATTTGAAGAACCTACTTCCATCCAAATCCTCTCCTAATTTCAAGTTCGTTAAGGGTGACATTGGTTCCGCTGACTTGGTTAATTTCTTGTTGATCACTGAATCCATTGATACTATCATGCATTTTGCTGCTCAAACACATGTTGACAACTCCTTCGGTAACTCTTTCGAGTTTACTAAGAACAACATCTACGGTACCCACGTTTTATTAGAAGCCTGTAAGGTTACCGGTCAAATTAGAAGGTTCATTCACGTTTCTACTGACGAAGTTTACGGTGAAACTGATGAAGATGCTGTCGTTGGTAATCATGAAGCTTCTCAATTATTGCCAACAAACCCATACTCTGCTACTAAGGCTGGTGCTGAAATGTTAGTTATGGCCTACGGTAGGTCTTATGGTTTGCCAGTCATCACTACTAGAGGTAATAATGTTTACGGTCCAAACCAATTCCCAGAAAAGTTAATTCCAAAGTTCATTTTGTTAGCCATGCGTGGCAAGCCATTACCAATCCATGGTGACGGTTCTAACGTAAGATCTTACTTGTATTGTGAAGATGTTGCTGAAGCTTTCGAAGTTATTTTACATAGAGGTGAAGTTGGTCACGTTTATAACATTGGTACTAAGAAGGAGAGGAGGGTTATCGATGTTGCTAAAGATGTCTGTAATCTATTTTCAATGGATCCAGAAACAAGCATTAAGTTTGTTGAAAACCGTCCATTCAACGACCAAAGATACTTCCTGGACGACCAAAAGCTAAAGATTTTGGGTTGGTCAGAAAGAACAACCTGGCAAGAAGGTTTGAAGAAAACTATGGAATGGTACATTAACAACCCTAATTGGTGGGGTGACGTTTCAGGAGCCTTGTTGCCACACCCAAGAATGTTGATGATGCCTGGTGGTATTGAGAGACATTTTGATGGTTCTGAAGATTCTGATTCTACTGCTTCTCCAGTTTCTTCTAATTTGAATCAAACTAGAATGGTCGTTCCAGTGCCTAAGTCGGTATCTTCTCCAAGAAAGCCATCACTTAAGTTCTTGTTATATGGTCGTACTGGTTGGATTGGTGGTTTATTGGGTAAATTGTGTGAAAAACAAGGTATTCCCTACGAATACGGTAGAGGTAGACTTGAAGATAGAGCTTCCTTGTTAGCTGATATTCAAAACGTTAAGCCAACTCACGTGTTTAACGCAGCTGGTGTTACCGGTAGACCAAATGTAGACTGGTGTGAATCCCATAAACCAGAAACCATTAGAGCTAATGTTGCTGGTACATTGACTTTAGCTGACGTCTGTAGAGAACACGGTTTGTTAATGATGAACTTTGCTACAGGTTGCATTTTCGAATATGACGCTGCTCACCCAGAAGGTTCAGGTATTGGTTTCAAAGAAGAAGATACCCCTAACTTTGCCGGTTCTTTCTATTCAAAGACCAAAGCTATGGTTGAAGAATTGTTAAAGGAATTCGATAATGTGTGTACTTTGAGAGTTAGAATGCCAATTAGTTCAGACTTGAACAATCCAAGAAATTTCATCACCAAGATTTCTAGATATAATAAGGTTGTAAATATCCCAAACTCTATGACAGTTTTAGACGAATTATTACCAATTTCTATTGAAATGGCTAAGCGTAATTGTAGAGGTATTTGGAATTTCACTAATCCAGGTGTTGTTTCTCATAATGAAATCTTGGAAATGTATAAATCCTACATTGACCCAAATTTTAAATGGGCTAACTTCACTTTAGAGGAACAAGCTAAAGTTATCGTTGCTGCTAGATCTAACAACGAAATGGATGCTTCTAAATTGAAGAATGAATTTCCAGAATTGTTGCCAATCAAAGATTCTTTGATTAAGTACGTCTTTGAACCAAATCAAAAGTCTTTAGCCGCTTAA | |  |
| *AtNRE/ER* | | aaaaatttatcactactccaattatctacacatgttttgcttaatttttggtaatgtcacctaaatttaacaatttcctaaaatattcatgaaaaaaaactcatgggtcacaaatctcaaataagaaacgatgggtggtcaaaagaaaaaaactaaaaagattattgcttccccgagaaattgggataaaaagaagcaacaccgatcacaacgtctccctataccatctccttcagatccacacactctctcttatatctctccggcgaaaactttccgatcaccgacaaaaaaaaaaaatggttgcagacgcaaacggttcatcatcaagctcatttaacttcctaatctacggtaaaaccggatggatcggtggtttactcggtaaactctgcgaagctcaaggaatcacttacacttacggctccggtcgtcttcaagatcgtcaatcgatcgtcgccgacatcgaatccgtgaaacctagccacgtgttcaacgctgctggagtcaccggtcgtcctaatgttgattggtgcgaatctcacaaagttgagacgattcgtactaatgtcgccggaaccctaactctcgctgacatttgcagagagaaaggacttgttctgatcaattacgctacgggttgtatatttgagtatgattcgggtcatcctctcgggtcgggtattggattcaaggaggaggatactcctaatttcaccggatctttctactctaaaaccaaagctatggtggaggagctgctcaagaactatgaaaatgtatgcacgctaagagtgcgaatgccgatttcatcggatctaacaaacccgagaaacttcatcacgaagattgctcggtatgagaaagttgtggacatcccaaactcgatgacaatcctcgatgagcttctcccgatatcaatcgaaatggcgaagaggaacttaaccgggatctacaatttcactaacccgggtgttgtgagccacaacgagatcttggagatgtacagagactacattgacccgagttttacttggaagaacttcacattggaggaacaagctaaagtgattgtggcgccaaggagtaacaatgagcttgatgcaactaagttgaagactgagttccctgagttgatgtctatcaaagagtctctgatcaagttcgtgtttgagcccaacaagaagactgaagttaaagcttgaaatgtggttttgtaaggtaaacggttcttaaccccaaaaaaacaaatcaaatgaaagacaaaaaaagtttgggtttttaaaagaagtatcttttttttttttgggtttatgggctgaaaaaattgttgtgttttttgtattttatttttttgtttttctttttcccaatgttgttgtattgttaaaataaacttttgtttcattacaaataagaaatgtactattttgcattatttactcttgttaatcaaactatctttc | | |
| *AtRHM1-NRE/ER* | | ATGGCTTCCTACACTCCAAAGAATATTTTGATTACTGGTGCTGCTGGTTTCATTGCTTCTCACGTTGCTAATAGACTAATTAGATCCTACCCAGATTACAAGATAGTCGTTTTGGATAAATTAGATTACTGTTCTAACTTGAAGAACTTGAACCCATCTAAGCACAGCCCAAATTTTAAGTTCGTTAAGGGTGACATTGCCTCTGCTGATTTGGTTAATCACTTGTTAATTACCGAAGGTATCGACACTATTATGCATTTCGCTGCTCAAACTCACGTTGATAACTCTTTTGGTAATTCTTTTGAATTCACCAAGAATAATATATATGGTACTCATGTTTTGTTGGAAGCATGTAAGGTCACTGGTCAAATTAGAAGATTCATTCACGTTTCCACCGATGAAGTTTACGGTGAAACCGATGAAGATGCTTTGGTTGGTAACCACGAAGCTTCCCAATTGTTACCTACTAACCCATATTCTGCAACTAAAGCTGGTGCTGAAATGTTGGTTATGGCATACGGTAGAAGCTATGGTTTGCCAGTGATTACAACTAGAGGTAATAACGTTTACGGTCCAAACCAATTCCCTGAAAAGTTGATTCCTAAGTTCATCTTGTTGGCCATGAGAGGTCAAGTTTTACCAATCCATGGCGATGGTTCTAACGTCAGATCTTACTTATACTGTGAAGATGTTGCTGAAGCTTTTGAAGTCGTTTTGCATAAAGGTGAAGTTGGTCACGTTTATAATATTGGTACTAAGAAAGAAAGAAGAGTTAATGACGTTGCTAAAGATATTTGTAAATTGTTCAACATGGATCCAGAAGCTAATATCAAGTTTGTAGATAACCGTCCTTTTAATGATCAAAGATACTTTTTGGACGACCAAAAGTTGAAAAAGTTAGGTTGGTCTGAACGTACTACTTGGGAAGAAGGTTTGAAGAAAACTATGGATTGGTATACTCAAAACCCAGAATGGTGGGGCGATGTCTCTGGCGCTTTATTGCCACATCCTAGAATGTTGATGATGCCAGGTGGTCGTCATTTCGACGGTTCCGAAGATAATTCTTTGGCCGCTACTTTGTCCGAAAAGCCATCTCAAACTCATATGGTTGTTCCTTCTATGGTTGCTGATGCTAACGGTTCCTCTTCTTCTTCTTTCAATTTCTTGATCTACGGTAAAACCGGTTGGATTGGTGGTTTGTTGGGTAAATTGTGTGAAGCTCAAGGTATTACTTATACATACGGTTCTGGTAGATTGCAAGATAGACAATCTATTGTTGCTGACATTGAATCTGTTAAGCCATCTCATGTTTTCAACGCTGCTGGTGTTACTGGTAGACCAAACGTTGATTGGTGTGAATCTCATAAGGTTGAAACAATTAGAACTAATGTTGCTGGTACCTTGACTTTGGCCGATATTTGTAGAGAAAAAGGTTTAGTTTTAATTAACTACGCTACTGGTTGTATTTTTGAATACGATTCCGGTCATCCATTGGGTTCTGGTATTGGTTTTAAGGAAGAAGATACTCCAAATTTCACTGGTTCTTTCTACTCTAAAACTAAGGCTATGGTTGAAGAATTGTTAAAGAACTACGAAAATGTCTGTACTTTAAGAGTTAGGATGCCAATTTCCTCTGATTTGACTAACCCAAGAAACTTCATTACTAAAATTGCTAGATACGAAAAAGTTGTTGATATTCCAAACTCCATGACTATCTTGGATGAATTGTTACCAATTTCTATTGAAATGGCTAAAAGAAACTTGACTGGTATCTACAATTTCACTAACCCAGGTGTCGTTTCTCATAACGAAATTTTGGAAATGTACAGAGATTACATTGATCCATCTTTTACTTGGAAGAATTTCACTTTGGAAGAACAAGCTAAGGTTATTGTTGCTCCAAGATCTAACAATGAATTGGATGCTACTAAGTTGAAAACTGAATTCCCAGAATTGATGTCTATTAAGGAATCTTTAATTAAGTTTGTTTTTGAACCAAACAAGAAAACTGAAGTTAAGGCTTAA | |  |
| *OlRHM- NRE/ER* | | ATGGCTTCTCATACCCCAAAGAACATTTTGATTACCGGTGCCGCAGGTTTCATTGCTTCTCATGTTGCTAATAGATTGGTTAGAAAGTATCCACAATACAAAATTGTTGTTTTGGATAAATTAGATTACTGTTCTAACTTGAAGAACTTGAGACCTTCACAATTGTCCCCAAACTTTAAGTTCGTTAAAGGTGACATCGCTTCAGCCGACTTAGTTAATTATTTGTTGATTACTGAATCTATTGACACTATTATGCATTTCGCTGCTCAAACTCACGTTGACAACAGTTTCGGTAATTCTTTTGAATTCACAAAAAATAATATCTACGGTACTCACGTTTTGTTGGAAGCTTGTAAAGTTACTGGTCAAATTAAGCGTTTCATCCATGTTTCTACTGATGAAGTCTACGGTGAAACCGATGAGGACGCCGTAGTCGGTAACCACGAAGCTTCCCAATTGTTACCAACTAACCCATACTCTGCTACTAAGGCTGGTGCTGAAATGTTAGTTATGGCTTACGGTAGATCTTATGGTTTGCCAGTTATTACTACCAGAGGTAATAATGTTTATGGTCCAAACCAATTTCCTGAAAAGTTGATTCCAAAATTCATTTTATTAGCTATGAGAGGTAAATCTTTGCCAATTCATGGTGACGGTTCTAACGTCCGTTCCTACTTATATTGTGAAGATGTTGCTGAAGCTTTCGAATTAATTTTACATAAAGGTGAAGTTGGTCATGTTTATAACATTGGTACCAAAAAAGAAAGAAGAGTCATTGATGTAGCTAAAGACGTTTGTAAGTTATTCTCTTTGGATGCTGATTCCGTTATCAAATTCGTTGAAAATAGACCTTTCAATGATCAAAGATATTTCTTGGATGACCAAAAATTGACTAACTTGGGTTGGTCTGAAAGAACCACTTGGGAAGAAGGTTTGAGAAAGACTATGGAATGGTATACTTCTAATCCAGAATGGTGGGGAGATGTTTCTGGTGCCTTATTGCCTCACCCTCGTATGTTAATGATGCCTGGTATTGAAAAACAATTTGATGGCCCAGCTGATATTAACGGCACTTTGTCTGAATTAATGAAAAAGCCAACACAAACTGAAAAAGAAGTTGAAGCTATGGTTGCTGATGCTAACGGTTCCTCTTCTTCTTCTTTCAATTTCTTGATCTACGGTAAAACCGGTTGGATTGGTGGTTTGTTGGGTAAATTGTGTGAAGCTCAAGGTATTACTTATACATACGGTTCTGGTAGATTGCAAGATAGACAATCTATTGTTGCTGACATTGAATCTGTTAAGCCATCTCATGTTTTCAACGCTGCTGGTGTTACTGGTAGACCAAACGTTGATTGGTGTGAATCTCATAAGGTTGAAACAATTAGAACTAATGTTGCTGGTACCTTGACTTTGGCCGATATTTGTAGAGAAAAAGGTTTAGTTTTAATTAACTACGCTACTGGTTGTATTTTTGAATACGATTCCGGTCATCCATTGGGTTCTGGTATTGGTTTTAAGGAAGAAGATACTCCAAATTTCACTGGTTCTTTCTACTCTAAAACTAAGGCTATGGTTGAAGAATTGTTAAAGAACTACGAAAATGTCTGTACTTTAAGAGTTAGGATGCCAATTTCCTCTGATTTGACTAACCCAAGAAACTTCATTACTAAAATTGCTAGATACGAAAAAGTTGTTGATATTCCAAACTCCATGACTATCTTGGATGAATTGTTACCAATTTCTATTGAAATGGCTAAAAGAAACTTGACTGGTATCTACAATTTCACTAACCCAGGTGTCGTTTCTCATAACGAAATTTTGGAAATGTACAGAGATTACATTGATCCATCTTTTACTTGGAAGAATTTCACTTTGGAAGAACAAGCTAAGGTTATTGTTGCTCCAAGATCTAACAATGAATTGGATGCTACTAAGTTGAAAACTGAATTCCCAGAATTGATGTCTATTAAGGAATCTTTAATTAAGTTTGTTTTTGAACCAAACAAGAAAACTGAAGTTAAGGCTTAA | |  |
| *VvRHM- NRE/ER* | | ATGGCTACCCACACTCCAAAGAATATTTTGATTACTGGTGCCGCCGGTTTCATTGCTTCTCATGTTGCTAATAGATTGATTAGAAACTACCCTGATTACAAAATCGTTGTTCTAGATAAGTTGGATTACTGTTCTAATTTGAAGAACCTACTTCCATCCAAATCCTCTCCTAATTTCAAGTTCGTTAAGGGTGACATTGGTTCCGCTGACTTGGTTAATTTCTTGTTGATCACTGAATCCATTGATACTATCATGCATTTTGCTGCTCAAACACATGTTGACAACTCCTTCGGTAACTCTTTCGAGTTTACTAAGAACAACATCTACGGTACCCACGTTTTATTAGAAGCCTGTAAGGTTACCGGTCAAATTAGAAGGTTCATTCACGTTTCTACTGACGAAGTTTACGGTGAAACTGATGAAGATGCTGTCGTTGGTAATCATGAAGCTTCTCAATTATTGCCAACAAACCCATACTCTGCTACTAAGGCTGGTGCTGAAATGTTAGTTATGGCCTACGGTAGGTCTTATGGTTTGCCAGTCATCACTACTAGAGGTAATAATGTTTACGGTCCAAACCAATTCCCAGAAAAGTTAATTCCAAAGTTCATTTTGTTAGCCATGCGTGGCAAGCCATTACCAATCCATGGTGACGGTTCTAACGTAAGATCTTACTTGTATTGTGAAGATGTTGCTGAAGCTTTCGAAGTTATTTTACATAGAGGTGAAGTTGGTCACGTTTATAACATTGGTACTAAGAAGGAGAGGAGGGTTATCGATGTTGCTAAAGATGTCTGTAATCTATTTTCAATGGATCCAGAAACAAGCATTAAGTTTGTTGAAAACCGTCCATTCAACGACCAAAGATACTTCCTGGACGACCAAAAGCTAAAGATTTTGGGTTGGTCAGAAAGAACAACCTGGCAAGAAGGTTTGAAGAAAACTATGGAATGGTACATTAACAACCCTAATTGGTGGGGTGACGTTTCAGGAGCCTTGTTGCCACACCCAAGAATGTTGATGATGCCTGGTGGTATTGAGAGACATTTTGATGGTTCTGAAGATTCTGATTCTACTGCTTCTCCAGTTTCTTCTAATTTGAATCAAACTAGAATGGTCGTTCCAGTGATGGTTGCTGATGCTAACGGTTCCTCTTCTTCTTCTTTCAATTTCTTGATCTACGGTAAAACCGGTTGGATTGGTGGTTTGTTGGGTAAATTGTGTGAAGCTCAAGGTATTACTTATACATACGGTTCTGGTAGATTGCAAGATAGACAATCTATTGTTGCTGACATTGAATCTGTTAAGCCATCTCATGTTTTCAACGCTGCTGGTGTTACTGGTAGACCAAACGTTGATTGGTGTGAATCTCATAAGGTTGAAACAATTAGAACTAATGTTGCTGGTACCTTGACTTTGGCCGATATTTGTAGAGAAAAAGGTTTAGTTTTAATTAACTACGCTACTGGTTGTATTTTTGAATACGATTCCGGTCATCCATTGGGTTCTGGTATTGGTTTTAAGGAAGAAGATACTCCAAATTTCACTGGTTCTTTCTACTCTAAAACTAAGGCTATGGTTGAAGAATTGTTAAAGAACTACGAAAATGTCTGTACTTTAAGAGTTAGGATGCCAATTTCCTCTGATTTGACTAACCCAAGAAACTTCATTACTAAAATTGCTAGATACGAAAAAGTTGTTGATATTCCAAACTCCATGACTATCTTGGATGAATTGTTACCAATTTCTATTGAAATGGCTAAAAGAAACTTGACTGGTATCTACAATTTCACTAACCCAGGTGTCGTTTCTCATAACGAAATTTTGGAAATGTACAGAGATTACATTGATCCATCTTTTACTTGGAAGAATTTCACTTTGGAAGAACAAGCTAAGGTTATTGTTGCTCCAAGATCTAACAATGAATTGGATGCTACTAAGTTGAAAACTGAATTCCCAGAATTGATGTCTATTAAGGAATCTTTAATTAAGTTTGTTTTTGAACCAAACAAGAAAACTGAAGTTAAGGCTTAA | |  |
| *Cs1,6-RhaT* | | ATGCATGCTCCATCCAACCAACACCACAAGATGGGTACTGAATCTGCTGAAGCTGACCAATTGCACGTTGTTATGTTCCCATGGTTTGCTTTCGGTCACATCTCTCCATTCGTTCAATTGTCTAATAAGTTATCTTTGCATGGTGTTAAGGTCTCTTTCTTCTCCGCTCCAGGTAACATCCCAAGAATTAAGTCATCTTTGAACTTGACACCTATGGCTGACATCATTCCATTACAAATTCCTCATGTCGATGGTTTGCCACCAGGTTTAGATTCTACTTCAGAAATGACTCCACATATGGCTGAATTGTTGAAGCAAGCTTTGGATTTGATGCAACCACAAATTAAGACTTTGTTGTCCCAATTGAAACCACATTTTGTTTTCTTTGATTTCACTCACTATTGGTTACCAGGTTTAGTTGGTTCCCAATTGGGTATTAAGACTGTCAACTTCTCCGTCTTTTCTGCCATTTCACAAGCTTATTTGGTTGTTCCAGCTAGAAAGTTGAACAACTCTTTAGCTGATTTGATGAAGTCTCCAGATGGTTTCCCAGCTACTTCTATTACTTCTCTAGATGAATTCGTTGCCAGAGATTACTTGTACGTCTACACCAAATTCAATGGTGGTCCATCCGTTTATGAAAGAGGTATCCAAGGTGTTGATGGTTGTGACGTTTTAGCTATCAAAACTTGTAATGAAATGGAAGGTCCTTACTTAGACTTTGTTAGAACTCAATTCAAAAAACCAGTCTTGTTAACTGGTCCATTGGTTAATCCTGAACCACCATCAGGTGAATTAGAAGAAAGATGGGCTAATTGGTTGGGTAAATTCCCACCAAAGTCTGTTATCTATTGTTCTTTCGGTTCTGAAACTTTCTTGACCGTCGATCAAATTAAGGAATTAGCTATCGGTTTGGAAATTACCGGTTTGCCTTTCTTTTTGGTTCTAAACTTCCCTCCAAACGTCGATGGTCAATCCGAATTGGTTCGTACTTTGCCACCAGGTTTCATGGACAGAGTTAAGGACAGAGGTGTCGTTCATACTGGTTGGGTTCAACAACAATTAATTTTGAGACATGAATCTGTTGGTTGTTACGTTTGTCATTCCGGTTTTTCTTCTGTCACCGAAGCTGTTATTTCTGATTGCCAATTGGTTTTATTGCCATTGAAGGGCGATCAATTTTTGAACTCTAAGTTGGTTGCTGGCGATTTGAAGGCTGGTGTTGAAGTTAATAGACGTGATCATGATGGTCACTTCGGTAAAGAAGATATTTTTAAAGCTGTTAAGACTGTTATGGTTGATGTTAATAAGGAACCAGGTGCTTCCATTAGAGCTAACCAAAAGTGGTGGAGAGAATTCTTGTTGAACGGTCAAATTCAAGATAAGTTTATTGCTGATTTTGTTAAGGATTTGAAGGCTTTAGCTTAA | |  |
| *Ci1,6-RhaT* | | atgtctatgaacggtaaagataaggaattgcatttggttatgttcccattcttcgccttgggtcatatttctccattcgttcaattgtctaacaagttaagttcttacccaggtattaagatttcattcttagctgcttccgcttctgttgatagaattaagtccatgttgaacccaattactactacccaaattattccattgactttgccacacgttgatggtttgccaaagggtgttgaatgtactgctgatacctctcctgctggtgccgaattgttaaaagttgccttggatttgatgcaaccacaaattaagaccttattaactcatttgaaaccagatttcgtctttttcgactttgctcaatggtggttgccacgtatggcttgtgaattgggtatcaaaactatttgtttctctgttttcatgaccattgctacttctttcttaatcgttccatctagattatctcacaatgaatcccaaaatttggaagaaatcaagaaaccacctcctggtttcccaaagactatcccactgaaaaccttcgaggcccagaactacacctacatcttcaagtcattccacggtactccatccgtcatcgacagattcattacatgcttggacggctgtaatgctattttggttaaatcttgtactgaaatggaaggtccatacattgattacttctcaaagcagtttaagaagcctgtcttgttgatcggcccagttgttccagaaccacacactggtcaattagaagatacttgggctaactggctgaaccaattccctaacaagtctgttatctattgtagtttcggtagtgaaacttatttgactgacaaccaaattaaggaattggccttaggtttggaattaactggtttgccattcttcttagtccttaatttctctactaacctaaattcttctgaacaattagaaagaactttgccacaaggtttcttagaaagagttaaagatattggtattgttcattctggttgggttcaacaaagacatattttggctcatgaatccgtcggttgttatttgtctcatgctggtttttcttctgttattgaaggtttagttaacgattgtcaattggtcatgttacctttaaagggcgatcagtttatgaactctaaattgattgaattagaatggcgtgttggtgttgaagtgtatagaagagatgaagatggttactttggtaaagatgatgtttttgaagctgtcaaatctgttatgatggaaacagaaaaggaaccagttaagtccattcgcgaaaaccacaagaaatggaaggagttcctgcaaaataatgaaatacaatctaactatataagtgacttagttgaaaacctacaagccttaacccaagacatagtcttgtaa | |  |
| *Cm1,6-RhaT* | | atgcatgctccatctaaccaacaccataagactggtactgaatctgctgaagatgatcaattgcatgttgttatgtttccatggtttgctttcggtcatattagaccattcgttcaattgtcaaacaagttatctttacatggtgttaaggtttctttcttctccgctccaggtaacattccaagaattaagtctagtttaaacttgactccaatggctgaaattattcctttgcaaattccacacgtggatggtttgccaccaggtttggatattacttctgaaatgacccctcacatggctgaattgttgaaacaagctttagatttgatgcaaccacaaattaagactttgttatctcaattgaagccacacttcgtctttttcgatttcactcactactggttgccaggtttagttggttcccaattaggtatgaaaacagtttacttttctgttttctctgctatttctcaagcttatcttttggttccagcccgtaagttaaacaattctttggctgacttgatcaaatctccagatagatttccagctgcttctatcatctctttgcatgaatttgttgctagagattacttgtatgtttacactaactttaatggtggtccatctgtttatgaaagaggtttccaagggatcggtggttgcgacgttttggccatcaagacctgtaacgagatggaaggtccctacgtcgacttcatgcgtacccaattcaagaagccagtgttgttgaccggtccattggtgaacgagcctccatccggtgaactggaagaacgttgggctaagtggctgggaaagtacccaccaaaatcggtcatctactgtagcttcggttctgaaaccttcctgaccgttgatcaaatcaaggaactggccttcggtttggaaattaccggtctgccattcttcttggttttgaacttgcccccaaacgtcgacggtcaatctgaactggttagaattttgcccccagacttcatggatagagttaaggacagaggtgtcgtgcacactggttgggtccaacagcagttgatcttgagacatgaatctgtcggttgttacgtctgtcactccggcttctcttccgtcacggaagctgtcatctccgactgccaattggtcttgctgcctctgaagggtgaccaattcttgaactccaagttggtcgccggcgacttgaaggccggtgttgaagtcaaccgtcgcgatcacgatggtcacttcggtaaagaggacattttcaaggccgtcaagaccgtcatggtcgacgtcaacaaggaaccaggtgcttccactagggccaaccaaaagtggtggagggaattcttgctgaacggtcaaatccaagataagttcatcgccgatttcgttaaggatttgaaggctttggcttaa | |  |
| *Cc1,6-RhaT* | | atgcacgcttcctccaacatgcacgctccatctaaccaacaccataaaatggggaccgagtccgccgaagctgatcagcttcatgtcgtcatgttcccatggttcgctttcggtcatatttctccattcgttcaattatctaataagttatctttgcacggtgtcaaagtttcattcttctctgctccaggtaatattccaaggattaagtctagtttaaacttaactccaatggctgacattattccattgcaaatcccacatgttgatggtttgccaccaggtttagattcaacctctgaaatgaccccacatatggctgaattattgaagcaagctttagatttgatgcaaccacaaattaagaccttattgtctcaattgaaaccacacttcgttttctttgatttcactcattactggttgccaggcttagttggttctcaattaggtattaagactgttaatttttccgtttttagcgcaatttctcaagcttacttagttgttccagctagaaagttgaataactctttagctgatttgatgaaatctccagatggttttccagctacttctattacttctttggatgaattcgttgctagagattatctttatgtttacaccaagtttaacggtggtccatctgtttacgaaagaggtattcaaggtgttgatggttgtgatgttttagccattaagacttgtaatgaaatggaaggtccatacttagatttcgttagaacccaattcaaaaaaccagtcttattgacaggtcctttggtcaacccagaaccaccatctggggaattggaagaaagatgggctaagtggttgtgtaaatacccaccaaagtccgttatctattgttctttcggttccgaaaccttcctaaccgttgatcaaatcaaggaattagctattggtttggaaattactggtttaccattcttcttagttttaaactttccaccaaacgttgatgctcaatctgaattagtcagaaccttgccacctggttttatggatagagttaaagacagaggtgttgttcatactggttgggtccaacaacaattgatcttaagacatgaatctgttggttgctatgtttgtcattctggtttttcttctgttactgaagctgttatctctgattgtcaattggttttgttaccattgaaaggtgaccaattcttaaactctaagttagttgccggtgacttgaaggctggtgttgaagttaatagaagagatcacgatggtcactttggtaaagaagatattttcaaggctgttaagactgttatggttgatgttaataaggaaccaggtgcttctattagagctaaccaaaagtggtggcgtgaattcttgttgaatggtcaaattcaagataagtttattgctgattttgttaaggatttgaaaactttggcttaa | |  |
| *SmF3'H* | | atgactatcttgccattgttgttgtacgctagtataaccggtttgctaatctacgtcctattgaacttgaggactactcctaggtctaaccacttgccattgcctccaggcccgacaccctggcctataataggtaacctcccccacctggggcgtattccccatcacgccttagctgccatggccactaagtacggtccattaatgcatttgagattgggtgtcgttgatgttgttgttgctgcttcagcttctgttgctgctcaattcttaaaggtccatgatgctaatttcgcttctcgtccaccaaattccggtgctaagcacattgcttacaactatcaagatctagttttcgctccatacggtcaaaagtggagaatgttaagaaagatctgttcagtacacttattctctaataaggctttagatgattttagacacgttagacaagaagaagtcgctattttagttagagctttagctggtgctggacgtagtaccgccgctgctttgggtcaattgttgaatgtttgtaccactaacgctttggcccgtgttatgttaggtagacgtgtttttgttgatggttctgaaggtaacagagatgctgatgagtttaaggatatggttgttgaagtaatggttttggctggtgaattcaacattggtgactttattccagctttagactggttggatttgcaatctgttactaaaaagatgaagaagttacacttgagatttgactctttcttgaacaagattttggaagatcatagaaacggtggtgacgttacctctggtaatgttgacttattgtccactttgattagtttaaaagacgatgccgatggtgagggtggtaaattatctgatattgagatcaaggctttgttattgaacttattcactgctggtaccgacacctcttcatctacagttgaatgggctatggctgaattaatcagacacccacaattattgaaacaagctcaagaagaacttgatactgttgttggtaaagacagattggtttctgaattggatttgtctagattaactttcttagaagctattgttaaggaaacttttagattgcacccaagtactccattgtccttaccaagaatcgcttctgaatcttgtgaagtcgatggttactatattccaaagggtactacattattagttaatgtttgggctattgctagagatccaaagatgtggactgatccattagaattcagaccaactagattcttgcctggtggtgaaaagccaaacgctaacgttaagggtaatgattttgaaatcatccctttcggtgctggtagaagaatttgtgcaggtatgtcattaggtttaagaatggtccaattgttaactgctactttagtacatgcttttgattggaagttggcaaatggtttggatccagaaaaattgaacatggaagaagcttatggtttgactttgcaaagagctgctccattgatggttcatccaactccaagattagctccacacttgtacgaatcttctcaaggtttgtaa | |  |
| *GhF3'H* | | atgacacctttaaccttgttgatcggtacatgcgttaccggtttattcttatacgttttgcttaatagatgtactagaaaccctaatagattgccacctggtccaacaccatggccagtcgttggtaacttgccacacttgggtaccatcccacatcattctctagctgccatggctaagaaatatggtccattgatgcacttgagattgggttttgttgatgttgttgttgctgctagtgcttctgtcgctgctcaatttttgaaaacacacgatgctaatttcgctgatcgtccaccaaactctggtgctaagcacatcgcttacaactaccaagacctcgttttcgctccatacggcccaagatggagaatgttgagaaagatttgttctgttcacttgttctcaaccaaggctttggatgacttcagacatgttagacaagaagaagtcgctattttggctagagctttagttggtgctggcaaatccccagttaagttaggtcaattgttgaacgtttgtaccactaatgctttggctagagttatgctgggtagaagagtttttgattcaggtgacgcccaagctgatgaattcaaggatatggtggttgaattgatggttctagctggtgaattcaacatcggtgactttattccagtcttagattggttagacttgcaaggtgtcactaagaagatgaagaagttgcacgcaaaatttgactccttcttgaatactattcttgaagaacataagacaggtgctggtgacggtgttgcctctggtaaagttgatctactttctactttgatttcattgaaggatgatgctgatggtgaaggtggcaagttgtctgatattgagattaaggctttattgttgaacttgttcaccgctggtactgatacttcatcatctactatcgaatgggccattgctgaattgattagaaaccctcaattgttgaatcaagctagaaaggaaatggatactatcgtcggtcaagatagattggttactgaatctgatctaggtcaattaactttcttgcaagcaatcattaaggaaacttttagattgcatccatctactccattatctttgcctagaatggctttggaatcttgtgaagttggtggttattatatcccaaagggctccaccttgttagttaacgtctgggctattagtagagatccaaagatttgggctgacccattagaattccagccaactagattcctaccaggtggtgaaaagccaaacactgatattaagggtaatgatttcgaagtgattccatttggtgctggtagaagaatttgtgttggtatgtctttgggtttgagaatggttcaattgttaactgctaccttaattcatgcttttgattgggaattagctgatggtttgaaccctaagaagttaaacatggaagaagcttacggtttgactttgcaaagagccgcccctttagtagtccaccctagacctagattagcgccacatgtttatgaaaccactaaggtctaa | |  |
| *ThF3'H* | | ATGAGCATCCTGATCATCATCATCCTGTTCACCCTGGTGTTCGCAAGCCTGCTGTATCACCTGCTGAGCGGCCCGGCGCACCGTCTGCCGCCGGGCCCGCGTGGTTGGCCGATCCTGGGTAACCTGCCGCAGCTGGGTCCGAAACCGCACCAGACCCTGCACGCGCTGAGCAAAGCACACGGTCCGCTGTTCCTGCTGCGTCTGGGCTCTGTTGACGTTGTTGTGGCGGCCAGCGCGGCGGTTGCGGCTGCGTTCCTGCGTCAGCATGATGCGATCTTCTCTAACCGTCCGCCGAACTCCGGTGCGGAACACATCGCGTACAACTACCAGGACCTGGTTTTCGCACCGTATGGTCCGCGCTGGCGTCACCTGCGTAAACTGTGCAGCCTGCACTTGTTCAGCTCCAAAGCGCTGGATGATCTGCGCCCGATCCGTGAACAGGAAATTCAGCGTCTGACTCGTTCTCTCCTGGTTGGTCGTCCGGTTAACCTGGGTGAAGCTGTAAACGTGTGTGCGACCAACGCTCTGGCTCGTGCGACCGTTGGCTGGCGTGTGTTCGTGGGCGAAGGCGAAGATACTGCTGCGGAAGAATTTAAAGAAATGGTTCTGGAAATCATGAAACTGGCCGGCGTTTTCAACATCGGTGACTTCGTGCCGGGTATTGGCTGGATGGATCTGCAGGGTGTTGTTGGCAAAATGAAAAAACTGCACAAACGCATGGATGCGTTCCTGGATAAACTGATTAACGAACACAAACGCGGCTCTGGCGGCCGTGATCTGCTGTCTGTGCTGGTTAGCCTGAAAGACGACGAAGGTGAAGATGAAGCGAACAAACTGAGCCACACCGAAATCAAAGCACTGCTGCTGAACCTGTTCACCGCTGGCACCGATACCACCAGCTCTACCGTTGAATGGGCTCTGGCGGAACTGATCCGTCACCCGACGCTGCTGCACAAAGCGCAGCAGGAACTGGACAACGTGGTGGGTCGCCAGCGTCTGGTGTCTGAAACTGACCTGCCGAACCTGCCGTTCCTCCAGGCGATCATTAAAGAGACCTTCCGCCTGCACCCGTCTACCCCGCTGAGCCTGCCGCGCATTAGCAGCGAACCGTGCGAAATCAACGGTTATTATATCCCGAAAAACGCAACTCTGCTGGTTAACGTTTGGGCAATTGCTCGTGATCCGGCTGTATGGAGCGATCCGCTGGAATTCAAACCGGAACGTTTTATGCCGGGCGGTGAAAAAGCGAATGTTGATGTGAAAGGCAACGACTTTGAAGTTATTCCGTTCGGCGCAGGCCGTCGTATCTGCGCAGGCATGTCCCTGGGCCTGCGTATGGTGCAGTTCATGACCGCAACCCTGGTTCACGGCTTCGAATGGGGCCTGCCGGAAGGTGTTAACGCTGAAAAACTGGATATGGAAGAAAGCTACGGCCTGACCCTGCAGCGTAAAGTTCCGCTGACCGTTCAGCCGATCCCGCGTCTGGTTCGTGGTGCGTATGAAGTTGTTGAATGCTAA | |  |
| *SmCPR* | | atgcaatctgattcctccttggaaacttcttctttcgatttgattactgctgccttaaaggaaaaggttattgacactgctaacgcttctgactctggtgactccactatgccaccagctttggctatgatcttagaaaatagagaattgttcatgatgttaacgactactgtcgctctattgttgggtttcattgtcgtttcattctggaaaagatcttctgaaaagaagtctgccaaagacttggaattgccaaaaattgttgttccaaagagacaacaagaacaagaagtcgacgacggcaagaagaaagttacaattttgttcggtacacaaactggtactgccgaaggtttcgccaaggctttgttggaagaagctaaggctagatatgaaaaggccacctttaaggtcgttgatttggatgattatgctgtcgacgatgatgaatacgaagaaaagctgaaaaaagaatcattcgctttcttcttcttagctacatacggtgacggtgaaccaactgataacgctgcaagattctataaatggttcacagaaggtggtgaaaagggtgtttggttggaaaagttgcaatacggtgtttttggtttaggtaatagacaatatgaacattttaataagattgctaaagaagttgatgatggtttggcagaacaaggcgctaagaggttggttcctgtcggtttgggagatgatgatcaatctattgaagatgatttcactgcttggaaggaattggtttggcctgaattggatgaattattgcgtgatgaagatgacaagggtgtcgctactccatacactgctgccattccagagtatagagttgtttttcacgaaaagcatgatacttctgctgaggaccaaattcaaactaatggtcatgctgtgcatgatgcccaacatccatgtagatccaacgttgcagttaagaaggaattgcataccccagaatcggataggtcgtgcacccatctagaatttgacatttctcatactggtttgtcatatgaaaccggtgaccatgttggtgtttactgtgaaaatttgtctgaagtcgtcgaagaagctgaaagattgatcggtttgccatctgatacctatttctccgtccatactgataacgaagatggtacccctttaggtggtgcttctttacttcctccattcccaccatgtactttgagaaaggctttggctaattatgctgatgtgttgacctccccaaagaagtctgctttgattgctttagctgctcacgcttctgatcctactgaagctgaaagattgaagtttttggcctctccagccggtaaagatgaatactctcaatggattattgcctctcaacgttctttgttagaagttatggaagcttttccttctgctaagccaccattgggtgtctttttcgctgctattgctccaagacttcaaccaagatactattctatctcttcttcaccaaagatggctccatctagaattcacgttacctgcgccttagtctacgaaaaaaccccagccggtagactccataagggtatctgtagcacctggatgaagaacgctgttccaatgacagaatcgcaagactgttcctgggctccgattttcgttcgtacttctaacttcagactaccaaccgacccaaaagtgccagtcattatgatcggtccgggtaccggcctagctcctttcagaggtttcctgcaagaaaggttagccttaaaagaggccggtactgaattaggttcttctattttgttctttggttgtagaaacagaaaagtggacttcatctacgaaaatgaattgaaggactttgtggaaaatggcgctgttagtgaattgatcgtcgctttctccagagagggtccaaacaaagaatacgtccaacataaaatgtctgatagagcttctgatttgtggaacttgttgtctgaaggtgcttacctatatgtttgtggtgacgctaagggtatggctaaggatgtccacagaactttacacacaattgttcaagaacaaggtagtttggattcctccaaagctgaattgtatgttaaaaacttgcaaatgtcaggtagatacttgcgtgatgtctggtaa | |  |
| *AtCPR* | | ATGAGCTCTAGCTCTTCTTCTTCTACCAGCATGATTGATCTGATGGCGGCGATCATCAAAGGTGAACCGGTGATCGTGTCTGACCCGGCTAACGCGAGCGCATACGAATCTGTAGCGGCTGAACTGAGCTCTATGCTGATCGAAAACCGTCAGTTCGCTATGATCGTGACCACCAGCATCGCGGTTCTGATCGGTTGCATCGTTATGCTGGTTTGGCGCCGTAGCGGTAGCGGTAACTCCAAACGTGTTGAACCGTTGAAACCGCTGGTGATCAAACCGCGTGAAGAGGAAATCGATGATGGCCGTAAAAAAGTTACCATCTTCTTCGGCACTCAGACCGGCACGGCGGAAGGTTTCGCGAAAGCGCTGGGTGAAGAAGCCAAAGCTCGTTATGAAAAAACCCGCTTCAAAATCGTTGACTTGGACGATTACGCGGCAGATGATGATGAATATGAAGAAAAACTGAAAAAAGAAGATGTTGCGTTTTTCTTCCTGGCTACCTACGGTGACGGCGAACCGACTGATAACGCGGCTCGTTTCTATAAATGGTTCACTGAAGGTAACGATCGTGGTGAATGGCTGAAAAACCTGAAATACGGTGTGTTTGGCCTGGGTAACCGCCAGTATGAACACTTCAACAAAGTTGCGAAAGTGGTAGACGATATCCTGGTTGAACAGGGCGCACAGCGTCTGGTTCAGGTAGGCCTGGGTGATGATGACCAGTGCATCGAAGATGACTTCACCGCTTGGCGCGAAGCGCTGTGGCCGGAACTGGATACGATCCTGCGTGAAGAAGGTGATACCGCCGTGGCAACCCCGTATACCGCTGCGGTTCTGGAATACCGTGTTAGCATCCATGATAGCGAAGATGCTAAATTCAACGACATTAACATGGCGAACGGCAACGGCTACACTGTGTTCGACGCACAGCATCCGTACAAAGCGAACGTGGCCGTTAAACGTGAACTGCATACCCCAGAATCTGACCGCTCCTGTATCCACCTGGAATTCGACATTGCGGGCAGCGGTCTGACCTATGAAACCGGTGACCACGTTGGCGTTCTGTGTGACAACCTGTCTGAAACCGTTGATGAAGCTCTGCGTTTGCTGGACATGTCTCCGGATACCTATTTCAGTCTGCATGCTGAAAAAGAAGATGGTACCCCGATCTCATCCTCCCTCCCGCCACCGTTCCCGCCGTGCAACCTGCGCACTGCGCTGACCCGCTACGCATGCCTGCTGAGCTCCCCGAAAAAATCCGCGCTGGTAGCGCTGGCGGCGCACGCATCCGACCCAACCGAAGCCGAACGTCTGAAACACCTGGCCTCTCCGGCAGGCAAAGACGAATACTCTAAATGGGTGGTGGAAAGCCAGCGCTCTCTGCTGGAAGTTATGGCGGAATTCCCGAGCGCCAAACCGCCGCTGGGCGTGTTTTTCGCTGGCGTGGCTCCGCGCCTTCAGCCGCGTTTCTATTCCATCTCTAGCAGCCCGAAAATCGCTGAAACCCGCATTCACGTTACTTGCGCGCTGGTGTATGAAAAAATGCCGACTGGTCGTATCCACAAAGGCGTATGTAGCACCTGGATGAAAAACGCGGTTCCATACGAAAAATCTGAAAACTGCTCCTCCGCGCCGATCTTCGTGCGCCAGAGCAACTTTAAACTGCCGTCTGATTCTAAAGTTCCGATTATTATGATCGGTCCGGGTACCGGTCTGGCTCCGTTCCGTGGCTTCCTGCAGGAACGTCTGGCGCTGGTTGAATCTGGCGTTGAACTGGGTCCGTCCGTTCTGTTCTTCGGCTGCCGTAACCGCCGTATGGATTTCATCTACGAAGAAGAACTGCAGCGCTTTGTTGAAAGCGGTGCGCTGGCCGAACTGTCCGTCGCGTTCAGCCGTGAAGGTCCGACCAAAGAATATGTTCAGCACAAAATGATGGATAAAGCAAGCGATATCTGGAACATGATTTCTCAGGGCGCGTACCTGTACGTTTGTGGCGATGCAAAAGGTATGGCGCGTGATGTTCACCGTTCTCTGCACACCATCGCGCAAGAACAGGGTTCTATGGATTCTACCAAAGCGGAAGGTTTCGTGAAAAACCTGCAGACCTCTGGCCGTTACCTGCGTGACGTTTGGTAA | |  |
| *CrCPR* | | ATGGATTCTTCATCTGAAAAGTTGTCACCATTCGAATTGATGTCTGCTATCTTGAAAGGTGCAAAGTTGGATGGTTCCAACTCATCTGATTCAGGTGTTGCTGTTTCTCCAGCAGTTATGGCTATGTTGTTGGAAAACAAGGAATTGGTTATGATCTTGACTACATCTGTTGCTGTTTTGATCGGTTGTGTTGTTGTTTTGATCTGGAGAAGATCATCTGGTTCAGGTAAAAAAGTTGTTGAACCACCAAAGTTGATCGTTCCAAAGTCTGTTGTTGAACCAGAAGAAATCGATGAAGGTAAGAAAAAATTCACTATTTTCTTTGGTACTCAAACAGGTACTGCAGAAGGTTTTGCTAAAGCATTGGCTGAAGAAGCAAAAGCTAGATATGAAAAAGCTGTTATCAAGGTTATCGATATCGATGATTATGCTGCAGATGATGAAGAATACGAAGAAAAGTTCAGAAAGGAAACATTGGCTTTCTTTATCTTGGCAACTTATGGTGATGGTGAACCAACAGATAATGCTGCAAGATTCTACAAGTGGTTCGTTGAGGGTAACGATCGTGGTGATTGGTTGAAGAACTTGCAATACGGTGTTTTCGGTTTAGGCAACAGACAATACGAACATTTCAACAAGATTGCTAAAGTTGTTGATGAAAAAGTTGCAGAACAAGGTGGTAAAAGAATTGTTCCATTGGTTTTAGGTGATGATGATCAATGTATCGAAGATGATTTTGCTGCATGGAGAGAAAATGTTTGGCCAGAATTGGATAACTTGTTGAGAGATGAAGATGATACTACAGTTTCAACTACATACACTGCTGCAATTCCAGAATACAGAGTTGTTTTCCCTGATAAGTCTGATTCATTGATCTCTGAAGCAAATGGTCATGCTAACGGTTACGCAAACGGTAACACAGTTTACGATGCTCAACATCCATGTAGATCTAACGTTGCAGTTAGAAAGGAATTGCATACTCCAGCTTCTGATAGATCATGTACACATTTGGATTTCGATATTGCTGGTACTGGTTTATCATATGGTACAGGTGATCATGTTGGTGTTTACTGTGATAACTTGTCTGAAACTGTTGAAGAAGCTGAAAGATTGTTGAACTTGCCACCAGAAACTTACTTCTCTTTGCATGCAGATAAAGAAGATGGTACACCATTGGCTGGTTCATCTTTACCACCACCATTTCCACCATGTACATTGAGAACTGCTTTGACAAGATACGCAGATTTGTTGAACACACCTAAAAAATCTGCATTGTTAGCTTTGGCTGCATACGCTTCTGATCCAAACGAAGCAGATAGATTGAAGTACTTAGCATCACCAGCTGGTAAAGATGAATACGCTCAATCATTGGTTGCAAACCAAAGATCTTTGTTGGAAGTTATGGCTGAATTTCCATCTGCAAAACCACCATTGGGTGTTTTCTTTGCTGCAATTGCTCCAAGATTGCAACCAAGATTCTACTCAATCTCATCTTCACCAAGAATGGCACCATCTAGAATTCATGTTACTTGTGCTTTGGTTTACGAAAAAACACCAGGTGGTAGAATTCATAAAGGTGTTTGTTCAACTTGGATGAAAAATGCAATTCCATTGGAAGAATCTAGAGATTGTTCATGGGCTCCAATCTTCGTTAGACAATCTAACTTCAAGTTGCCAGCTGATCCAAAGGTTCCAGTTATTATGATTGGTCCAGGTACTGGTTTGGCACCATTTAGAGGTTTCTTGCAAGAAAGATTGGCTTTAAAAGAAGAAGGTGCTGAATTGGGTACAGCAGTTTTCTTTTTCGGTTGTAGAAACAGAAAGATGGATTACATCTACGAAGATGAATTGAACCATTTCTTGGAAATCGGTGCTTTGTCAGAATTGTTGGTTGCATTCTCTAGAGAAGGTCCAACTAAGCAATACGTTCAACATAAGATGGCAGAAAAGGCTTCAGATATCTGGAGAATGATCTCTGATGGTGCTTATGTTTACGTTTGTGGTGATGCTAAGGGTATGGCAAGAGATGTTCATAGAACTTTGCATACAATTGCACAAGAACAAGGTTCTATGGATTCAACTCAAGCTGAAGGTTTCGTTAAGAACTTGCAAATGACAGGTAGATACTTAAGAGATGTTTGGTAA | |  |

### Table S3

**Table S3.** Primers used in this study.

| Primers | Sequences (5’-3’) | |
| --- | --- | --- |
| PY26-F | | cttggcgtaatcatggtcatagctg |
| PY26-R | | gaaacattttgaagctatggccgtcgttttacaacgtcg |
| ApGT-F | | aataaaatacGTTCGCTCTATTAAGATGGGCTACCATAGCCACATCG |
| ApGT-R | | TTATTTAGAGATGCCGATGATTTCCAGCAGT |
| AtGT-F | | aataaaatacGTTCGCTCTATTAAGATGGGCACCCCGGTTGAAGTTA |
| AtGT-R | | TTAAACTTTTTCTTTCTGCAGTTTAACCAGGGTCA |
| BvGT-F | | aataaaatacGTTCGCTCTATTAAGatggatgataagtctcaacagcttcatattgttc |
| BvGT-R | | tcaagtagaaagacctctcaattcatcaattagagc |
| CjGT-F | | aataaaatacGTTCGCTCTATTAAGATGACCATGCGTAAACTGAACCTG |
| CjGT-R | | TTAGCAGCCGATGTTAGCCATCAGT |
| CsGT-F | | aataaaatacGTTCGCTCTATTAAGATGGTTCAGCACGGCCACAT |
| CsGT-R | | TTACAGGCAATCACCACCAACTTCTTC |
| MdGT-F | | aatacGTTCGCTCTATTAAGatggttcaacatagatttttgttagtaacatttccag |
| MdGT-R | | tcaatgtctagcatccttaagaaccttaatttgatca |
| SlGT-F | | aataaaatacGTTCGCTCTATTAAGATGGAAGATACCATCGTGATCTACACCA |
| SlGT-R | | TTAATCACGGGTCGCCAGCAG |
| StGT-F | | aataaaatacGTTCGCTCTATTAAGatggctatggaacaaaatgaagaaactgct |
| StGT-R | | tcaaaaagacttcttaaaagcacatctcttaatttcttcaatc |
| VlGT-F | | aataaaatacGTTCGCTCTATTAAGATGGATAAACACCACTTCCTGCTGC |
| VlGT-R | | TTAGTGGGTACGAACTTCCAGAGATTCAAC |
| pY26-GJ1-F | | gagctccagcttttgttccct |
| pY26-GJ1-R | | tctagaactagtggatcccccgg |
| GJ1-TDH1p-F | | gggaacaaaagctggagctcATGTCCCACCAGCCAACACT |
| GJ1-TDH1p-R | | tttgttttgtgTGTAAATTTAGTGAAGTACTGTTTTTTG |
| GJ1-AtRHM1-F | | AAATTTACAcacaaaacaaaATGGCTTCCTACACTCCAAAGAATATTTTG |
| GJ1-OlRHM-F | | TCACTAAATTTACAcacaaaacaaaATGGCTTCTCATACCCCAAAGAAC |
| GJ1-OlRHM-R | | atggcaccttgaggcttttgacattTTAAACCTTCTTATTTGGTTCAAAAACATACTTAA |
| GJ1-VvRHM-F | | TCACTAAATTTACAcacaaaacaaaATGGCTACCCACACTCCAAAGA |
| GJ1-VvRHM-R | | atggcaccttgaggcttttgacattTTAAGCGGCTAAAGACTTTTGATTTGGT |
| GJ1-NRS/ER-F | | CCTTCACAGTTGTTACTCCAATGGTTGCTGATGCTAACGGTT |
| GJ1-NRS/ER-R | | atggcaccttgaggcttttgacattTTAAGCCTTAACTTCAGTTTTCTTGTTTGGT |
| GJ1-Terr22-F | | aatgtcaaaagcctcaaggtgcc |
| GJ1-Terr22-R | | ataggttggcttccatgttggc |
| GJ1-AtGT-F | | caacatggaagccaacctatTTAAACTTTTTCTTTCTGCAGTTTAACCAGGG |
| GJ1-AtGT-R | | caacaagaaaagcCAAAATCATGGGCACCCCGGTTGAA |
| GJ1-CsGT-F | | caacatggaagccaacctatTTACAGGCAATCACCACCAACTTC |
| GJ1-CsGT-R | | caacaagaaaagcCAAAATCATGGTTCAGCACGGCCACAT |
| GJ1-ADH6p-F | | GATTTTGgcttttcttgttgttgtgttgaa |
| GJ1-ADH6p-R | | TACCTCACCTGAgttttgcttttttctcT |
| GJ1-SHM2p-F | | tgtagaaaaaaaaaaagaaaaaagaagtgaaatttttcaaatatttcaca |
| GJ1-SHM2p-R | | GTTAAGGAGGATTCGGTTTAAGCTGTTATG |
| GJ1-Cs1,6-RhaT-F | | TAAACCGAATCCTCCTTAACATGCATGCTCCATCCAACCAAC |
| GJ1-Cs1,6-RhaT-R | | ggggatccactagttctagaTTAAGCTAAAGCCTTCAAATCCTTAACAAAATCA |
| GJ1-Ci1,6-RhaT-F | | TAAACCGAATCCTCCTTAACatgtctatgaacggtaaagataaggaattgca |
| GJ1-Ci1,6-RhaT-R | | ggggatccactagttctagattacaagactatgtcttgggttaaggctt |
| GJ1-Cm1,6-RhaT-F | | CATAACAGCTTAAACCGAATCCTCCTTAACatgcatgctccatctaaccaacac |
| GJ1-Cm1,6-RhaT-R | | ggggatccactagttctagattaagccaaagccttcaaatccttaacg |
| GJ1-Cc1,6-RhaT-F | | TAAACCGAATCCTCCTTAACatgcacgcttcctccaacat |
| GJ1-Cc1,6-RhaT-R | | ggggatccactagttctagattaagccaaagttttcaaatccttaacaaaatcagc |
| pRS424-GJ2-F | | tttgttttgtgtgtaaatttagtgaagtactgttttttgt |
| pRS424-GJ2-R | | aattcttcgccagaggtttggtc |
| GJ2-TDH1p-F | | GAATCTATTAGAAGTGACATtttgttttgtgtgtaaatttagtgaagtactgttttttg |
| GJ2-TDH1p-R | | actaaagggaacaaaagctggaaaccacaccgtggggcct |
| GJ2-PGM1-F | | aaatttacacacaaaacaaaATGTCACTTCTAATAGATTCTGTACCAACAGTTG |
| GJ2-PGM1-R | | caaacctctggcgaagaattCTATGTGCGGACTGTTGGTTCG |
| GJ2-INO1p-F | | tgttacttctttttcactggaa |
| GJ2-INO1p-R | | gaagacgatgaggccggtg |
| GJ2-PGM2-F | | gaatgtaagcgtgacataacTTAAGTACGAACCGTTGGTTCTTCAGT |
| GJ2-PGM2-F | | gaatgtaagcgtgacataacTTAAGTACGAACCGTTGGTTCTTCAGT |
| GJ2-UGP1-F | | caaacctctggcgaagaattTCAATGTTCCAAGATTTGCAAATTACCAGT |
| GJ2-UGP1-R | | aaatttacacacaaaacaaaATGTCCACTAAGAAGCACACCAAAAC |
| GJ2-URA6-F | | caaacctctggcgaagaattctataagctatcacggatagcgtcttgg |
| GJ2-URA6-R | | aaatttacacacaaaacaaaatgacagctgccactacatcaca |
| GJ2-YNK1-F | | caaacctctggcgaagaatttcattcataaatccacttagcttgattagattccc |
| GJ2-YNK1-R | | aaatttacacacaaaacaaaatgtctagtcaaacagaaagaacttttattgcg |
| pY26-GJ3-F | | ACATTAACCTCTCTTACGTTCGCTCctggcgttacccaacttaatcgc |
| GJ3-SED1p-F | | CTCTCCTTCCGTGTAACGCGTTATGAATTATCTCCCAGACGGCACCG |
| GJ3-SED1p-R | | CTTAATAGAGCGAACgtattttattttgcttGTCT |
| GJ3-AtCPR-F | | aatacGTTCGCTCTATTAAGATGAGCTCTAGCTCTTCTTCTTCTACCAG |
| GJ3-AtCPR-R | | atAAATCATAAGAAATTCGCTTACCAAACGTCACGCAGGTAACG |
| GJ3-TEF1p-F | | ATTGACCACACCTCTACCGGCCACACACCATAGCTTCAAAATGTTTCT |
| GJ3-TEF1p-R | | TTTGTAATTAAAACTTAGATTAGATTGCTATGCTTTCTTTCTAAT |
| GJ3-GhF3'H-F | | TCTAATCTAAGTTTTAATTACAAAatgacacctttaaccttgttgatcgg |
| GJ3-GhF3'H-R | | gcgtgacataactaattacatgattagaccttagtggtttcataaacatgtggc |
| GJ3-SmF3'H-F | | TCTAATCTAAGTTTTAATTACAAAatgactatcttgccattgttgttgtacg |
| GJ3-SmF3'H-R | | gcgtgacataactaattacatgattacaaaccttgagaagattcgtacaagtgt |
| GJ3-ThF3'H-F | | TCTAATCTAAGTTTTAATTACAAAATGAGCATCCTGATCATCATCATCCT |
| GJ3-ThF3'H-R | | gcgtgacataactaattacatgaTTAGCATTCAACAACTTCATACGCACC |
| GJ3-CrCPR-F | | aataaaatacGTTCGCTCTATTAAGATGGATTCTTCATCTGAAAAGTTGTCACCAT |
| GJ3-CrCPR-R | | ataaaTCATAAGAAATTCGCTTACCAAACATCTCTTAAGTATCTACCTGTCATTT |
| GJ3-SmCPR-F | | aataaaatacGTTCGCTCTATTAAGatgcaatctgattcctccttggaaactt |
| GJ3-SmCPR-R | | aaatcatAAATCATAAGAAATTCGCttaccagacatcacgcaagtatctacct |
| GJ3-INO1p-F | | ATTGACCACACCTCTACCGGagGCCGGTGCCGATGT |
| GJ3-INO1p-R | | ATGATGATCAGGATGCTCATTGttacttctttttcactggaaaaaaaagggaat |
| GJ3-TDH1p-F | | ATTGACCACACCTCTACCGGATGTCCCACCAGCCAACACT |
| GJ3-TDH1p-R  pRS423-F  pRS423-R | | ATGATGATCAGGATGCTCATtttgttttgtgTGTAAATTTAGTGAAGTACTGTTTTTT  ataagtaaatgcatgtatactaaactcacaaattagagct  ttgaaaagctgtggtatggtgcac |
| CAS-F | | cctgatgcggtattttctccttacg |
| CAS-F | | ggagaaaataccgcatcaggcatagcttcaaaatgtttctactccttttttactct |
| ALG5-sg-F | | TAAATACAGTGAAAGCACCAgttttagagctagaaatagcaagttaaaataaggctagt |
| ALG5-sg-R | | TGGTGCTTTCACTGTATTTAgatcatttatctttcactgcggagaagtttc |
| GLC3-sg-F | | TTTACCTGCGTGGATCACAAgttttagagctagaaatagcaagttaaaataaggctagt |
| GLC3-sg-R | | TTGTGATCCACGCAGGTAAAgatcatttatctttcactgcggagaagtttc |
| FKS1-sg-F | | CGGTGTTCTATTCTTCTGTAgttttagagctagaaatagcaagttaaaataaggctag |
| FKS1-sg-R | | TACAGAAGAATAGAACACCGgatcatttatctttcactgcggagaagtttc |
| HUT1-sg-F | | TGGCGTTGGTCAATCCATCAgttttagagctagaaatagcaagttaaaataaggctagt |
| HUT1-sg-R | | TGATGGATTGACCAACGCCAgatcatttatctttcactgcggagaagtttc |
| YEA4-sg-F | | CGAATTTCTGGATGTTCATCgttttagagctagaaatagcaagttaaaataaggctagt |
| YEA4-sg-R | | GATGAACATCCAGAAATTCGgatcatttatctttcactgcggagaagttt |
| EXG1-sg-F | | TGGTGTCAACATTGGTGGTTgttttagagctagaaatagcaagttaaaataaggctagt |
| EXG1-sg-R | | AACCACCAATGTTGACACCAgatcatttatctttcactgcggagaagtttc |
| SPR1-sg-F | | AATTAGTAAACTGTAATCCTgttttagagctagaaatagcaagttaaaataaggctagt |
| SPR1-sg-R | | AGGATTACAGTTTACTAATTgatcatttatctttcactgcggagaagtttc |
| EGH1-sg-F | | CACTTCGAATGACTTACCAGgttttagagctagaaatagcaagttaaaataaggctagt |
| EGH1-sg-R | | CTGGTAAGTCATTCGAAGTGgatcatttatctttcactgcggagaagtttc |
| SCW2-sg-F | | GGAGTCATAAGCATAACATGgttttagagctagaaatagcaagttaaaataaggctagt |
| SCW2-sg-R | | CATGTTATGCTTATGACTCCgatcatttatctttcactgcggagaagtttc |
| SIM1-sg-F | | CACTGTAACTCCTGCTGCTTgttttagagctagaaatagcaagttaaaataaggct |
| SIM1-sg-R | | AAGCAGCAGGAGTTACAGTGgatcatttatctttcactgcggagaagtt |
| ALG5A-F | | caatatattattattttttaacattTATTGCGATGCtgctgaaaat |
| ALG5A-R | | TTTTGTTGTGCTTTGTGTAATTTTTGGCTG |
| ALG5B-F | | TTACACAAAGCACAACAAAATCCTGGCATGAGGTTGATGGC |
| ALG5B-R | | ATTGATATTACAAAAGTCGAAAATCATaaccaaaattcaaaaagt |
| GLC3A-F | | TATACCACACGTACGACTATAATTACAAACAACTATCAA |
| GLC3A-R | | TAAGCAATATAGTATAGAGTTTTATTCTTGACGGTTCTTTATACTTGGTTTGG |
| GLC3B-F | | ACTCTATACTATATTGCTTACTGAAAATCAAGActcaataa |
| GLC3B-R | | attgcagattcccttttatggattcctaaatcc |
| FKS1A-F | | TCGCGTTTTGATGAAGCACAGg |
| FKS1A-R | | TCAAGCAAGTATTGATTGTAGGTCTGACCGTTGTATGAAAGACTTGA |
| FKS1B-F | | TACAATCAATACTTGCTTGAACGCTTGATTTT |
| FKS1B-R | | TAATGGCTgcgtaaaaattttgtttcttgtTC |
| HUT1A-F | | GGAGAATAGGCAACCATCTTTAGTTTCAAG |
| HUT1A-R | | TTGTTGCGATCTTCACTAATACAAATATACGAATACTGCCTTGTttacttcttct |
| HUT1B-F | | ATTAGTGAAGATCGCAACAAATAAAAGttaatatttataaaatttGTCT |
| HUT1B-R | | GGATGCTATCTACTTACGATTTTCCCTATCG |
| YEA4A-F | | AAGACGTTTTTGTAACTATGTTCTACCTTTTTTAACG |
| YEA4A-R | | GGTTAGTTTATAATGTGTGTTTTTTAGATTCTCCCAATCCAATTACTTCCCTG |
| YEA4B-F | | ACACACATTATAAACTAACCTTAAATCCTACTTGTATGTT |
| YEA4B-R | | AAGAATAGGCAAACTCGTTTGAACATCAC |
| EXG1A-F | | ACGAGCCTGAGACAAGCCC |
| EXG1A-R | | TTTAGTTGGTAATTAActagaaaaagaaagtaaacaaaaaatcaaagg |
| EXG1B-F | | ctttctttttctagTTAATTACCAACTAAAACgaaagaaaggaaaacaCAAGACGTT |
| EXG1A-R | | tggattttctttttcggAGATTCTgtagaattttttt |
| SPR1A-F | | AGTTTGGCACGTCATTTATGAAATTCACA |
| SPR1A-R | | TTACTTCTAAAGTTTATAAAGATTTTATGCGGAGCA |
| SPR1B-F | | AAATCTTTATAAACTTTAGAAGTAAaattttgtggcatatatttAAGGTCTTGGGAAT |
| SPR1B-R | | atATGTCTTCATTATCTGAATCGGCTAGTGTAAG |
| EGH1A-F | | ttttttttttgacgaACCAGATTGTCAAATAAACAT |
| EGH1A-R | | ATGATCTGTTCTTattaaattcaaacaaTTTAGGTacgaa |
| EGH1B-F | | tttgaatttaatAAGAACAGATCATacaaaaattgaacCAAAAGCTATAGTagtca |
| EGH1B-R | | GCAAAGAGAAGTGGAATGATATAGATCAGATAGC |
| SCW2A-F | | CGAGTAAATATGAGTTTCTAACGTGCAATGT |
| SCW2A-R | | TGAATCAATCTCTAATAACTTCTATTATtaattattttatatttaaatTAGAATTTCAA |
| SCW2B-F | | AGTTATTAGAGATTGATTCATAGAGTCcgaatatttttttttgc |
| SCW2B-R | | GCGTTGGTAGTATTTTCACCGCA |
| SIM1A-F | | CTTTATTTTTAGTAAGTTATTTACCACAATTTTTCTCATACACCTTTAC |
| SIM1A-R | | ttctgcaaaaaaataaaggattGGATATTAGTTAGTAaagtg |
| SIM1B-F  X-4-F  X-4-R  XI-1-F  XI-1-R  XII-2-F  XII-2-R  XII-3-F  XII-3-R | | CCaatcctttatttttttgcagaaGCTACGTGACTActacttttccttttttttttct  GTTGCTGCTCTTGAATGGCGgttttagagctagaaatagcaagttaaaataaggctagt  CGCCATTCAAGAGCAGCAACgatcatttatctttcactgcggagaagttt  GCAATGCGATGTTAGTTTAGgttttagagctagaaatagcaagttaaaataaggctagt  CTAAACTAACATCGCATTGCgatcatttatctttcactgcggagaagtttc  TGAAACTCTAATCCTACTATgttttagagctagaaatagcaagttaaaataaggct  ATAGTAGGATTAGAGTTTCAgatcatttatctttcactgcggagaagttt  CTTTATGCATAGAGCTAATTgttttagagctagaaatagcaagttaaaataaggctagt  AATTAGCTCTATGCATAAAGgatcatttatctttcactgcggagaagttt |

### Table S4

**Table S4.** Plasmids involved in this study.

| Name | | Relevant genotype | Origin | |
| --- | --- | --- | --- | --- |
| pY26 | shuttle vector plasmid, Amp^R^, Ura 3 | | This lab | |
| pRS423 | shuttle vector plasmid, Amp^R^, His 3 | | This lab | |
| pRS424 | shuttle vector plasmid, Amp^R^, Trp 1 | | This lab | |
| P01 | PY26-SED1p-*ApGT* | | This study | |
| P02 | pY26-SED1p-*AtGT* | | This study | |
| P03 | pY26-SED1p-*BvGT* | | This study | |
| P04 | pY26-SED1p-*CjGT* | | This study | |
| P05 | pY26-SED1p-*CsGT* | | This study | |
| P06 | pY26-SED1p-*MdGT* | | This study | |
| P07 | pY26-SED1p-*SlGT* | | This study | |
| P08 | pY26-SED1p-*StGT* | | This study | |
| P09 | pY26-SED1p-*VlGT* | | This study | |
| P10 | pY26-TDH1p-*OlRHM*-*NRS/ER*-ADH6p-*CsGT*-SHM2p-*Cs1,6-RhaT* | | This study | |
| P11 | pY26-TDH1p-*AtRHM1*-*NRS/ER*-ADH6p-*CsGT*-SHM2p-*Cs1,6-RhaT* | | This study | |
| P12 | pY26-TDH1p-*VvRHM*-*NRS*-ADH6p-*CsGT*-SHM2p-*Cs1,6-RhaT* | | This study | |
| P13 | pY26-TDH1p-*OlRHM*-*NRS/ER*-ADH6p-*AtGT*-SHM2p-*Cs1,6-RhaT* | | This study | |
| P14 | pY26-TDH1p-*AtRHM1*-*NRS/ER*-ADH6p-*AtGT*-SHM2p-*Cs1,6-RhaT* | | This study | |
| P15 | pY26-TDH1p-*VvRHM*-*NRS*-ADH6p-*AtGT*-SHM2p-*Cs1,6-RhaT* | | This study | |
| P16 | pY26-TDH1p-*OlRHM*-*NRS/ER*-ADH6p-*AtGT*-SHM2p-*Ci1,6-RhaT* | | This study | |
| P17 | pY26-TDH1p-*OlRHM1*-*NRS/ER*-ADH6p-*AtGT*-SHM2p-*Cm1,6-RhaT* | | This study | |
| P18 | pY26-TDH1p-*OlRHM*-*NRS*-ADH6p-*AtGT*-SHM2p-*Cc1,6-RhaT* | | This study | |
| P19 | pRS424-TDH1p-*PGM1* | | This study | |
| P20 | pRS424-TDH1p-*PGM1*-INO1p-*UGP1* | | This study | |
| P21 | pRS424-TDH1p-*PGM2* | | This study | |
| P22 | pRS424-TDH1p-*PGM1*-INO1p-*PGM2* | | This study | |
| P23 | pRS424-TDH1p-*UGP1* | | This study | |
| P24 | pRS424-TDH1p-*URA6* | | This study | |
| P25 | pRS424-TDH1p-*YNK1* | | This study | |
| P26 | pRS424-TDH1p-*YNK1*-INO1p-*URA6* | | This study | |
| P27 | pRS424-TDH1p-*PGM2*-INO1p-*UGP1* | | This study | |
| P28 | pY26-SED1p-*AtCPR*-TEF1p-*GhF3'H* | | This study | |
| P29 | pY26-SED1p-*AtCPR*-TEF1p-*SmF3'H* | | This study | |
| P30 | pY26-SED1p-*AtCPR*-TEF1p-*ThF3'H* | | This study | |
| P31 | pY26-SED1p-*CrCPR*-TEF1p-*GhF3'H* | | This study | |
| P32 | pY26-SED1p-*CrCPR*-TEF1p-*SmF3'H* | | This study | |
| P33 | pY26-SED1p-*CrCPR*-TEF1p-*ThF3'H* | | This study | |
| P34 | pY26-SED1p-*SmCPR*-TEF1p-*GhF3'H* | | This study | |
| P35 | pY26-SED1p-*SmCPR*-TEF1p-*SmF3'H* | | This study | |
| P36 | pY26-SED1p-*SmCPR*-TEF1p-*ThF3'H* | | This study | |
| P37 | PY26-SED1p-*AtCPR*-INO1p-*ThF3'H* | | This study | |
| P38 | PY26-SED1p-*AtCPR*-TDH1p-*ThF3'H* | | This study | |
| P39 | PY26-INO1p-*AtCPR*-TDH1p-*ThF3'H* | | | This study |
| P40 | PY26-INO1p-*AtCPR*-TEF1p-*ThF3'H* | | | This study |
| P41 | PY26-INO1p-*AtCPR*-SED1p-*ThF3'H* | | | This study |
| P42 | PY26-TDH1p-*AtCPR*-SED1p-*ThF3'H* | | | This study |
| P43 | PY26-TDH1p-*AtCPR*-TEF1p-*ThF3'H* | | | This study |
| P44 | PY26-TDH1p-*AtCPR*-INO1p-*ThF3'H* | | | This study |
| P45 | PY26-TEF1p-*AtCPR*-INO1p-*ThF3'H* | | | This study |
| P46 | PY26-TEF1p-*AtCPR*-SED1p-*ThF3'H* | | | This study |
| P47 | PY26-TEF1p-*AtCPR*-TDH1p-*ThF3'H* | | | This study |
| P48 | pRS423-TDH1p-*AtCPR*-SED1p-*ThF3'H* | | | This study |
| Pcas1 | Cas9-gRNA-*ALG5* | | | This study |
| Pcas2 | Cas9-gRNA-*GLC3* | | | This study |
| Pcas3 | Cas9-gRNA-*FKS1* | | | This study |
| Pcas4 | Cas9-gRNA-*HUT1* | | | This study |
| Pcas5 | Cas9-gRNA-*YEA4* | | | This study |
| Pcas6 | Cas9-gRNA-*EXG1* | | | This study |
| Pcas7 | Cas9-gRNA-*SPR1* | | | This study |
| Pcas8 | Cas9-gRNA-*EGH1* | | | This study |
| Pcas9 | Cas9-gRNA-*SCW2* | | | This study |
| Pcas10 | Cas9-gRNA-*SIM1* | | | This study |
| Pcas11 | Cas9-gRNA-X-4 | | | This study |
| Pcas12 | Cas9-gRNA-XI-1 | | | This study |
| Pcas13 | Cas9-gRNA-XII-2 | | | This study |
| Pcas14 | Cas9-gRNA-XII-3 | | | This study |

### Table S5

**Table S5.** gRNA used in this study.

| Site | gRNA sequences |
| --- | --- |
| *ALG5* | TAAATACAGTGAAAGCACCA |
| *GLC3* | TTTACCTGCGTGGATCACAA |
| *FKS1* | CGGTGTTCTATTCTTCTGTA |
| *HUT1* | TGGCGTTGGTCAATCCATCA |
| *YEA4* | CGAATTTCTGGATGTTCATC |
| *EXG1* | TGTCAGAATTCCTATCGGTT |
| *SPR1* | AATTAGTAAACTGTAATCCT |
| *EGH1* | CACTTCGAATGACTTACCAG |
| *SCW2* | GGAGTCATAAGCATAACATG |
| *SIM1* | CACTGTAACTCCTGCTGCTT |
| X-4 | CGCCATTCAAGAGCAGCAAC |
| XI-1 | GCAATGCGATGTTAGTTTAG |
| XII-2 | TGAAACTCTAATCCTACTAT |
| XII-3 | CTTTATGCATAGAGCTAATT |

### Table S6

**Table S6.** *S. cerevisiae* strains involved in this study.

| Strains | Parent strain | | Relevant genotype | Origin |
| --- | --- | --- | --- | --- |
| CEN.PK2-  1D | CEN.PK  2-1D | *MATα*; *ura3-52*; *trp1-289*; *leu2-3,112*; *his3Δ1*; *MAL2-8C*; *SUC2* | | This lab |
| C800 | CEN.PK  2-1D | *Gal80::G418* | | This lab |
| E033 | C800 | Δ*EGH1*; Δ*EXG1*;Δ*SPR1* | | This lab |
| ZY00 | C800 | XI-5::GAL10p-*AtC4H*-GAL1p-*AtPAL2*-TDH3p-*AtATR2*-SED1p-*CYB5*; XI-2::GAL7p-*FjTAL*; X-3::GAL10p-*SjCHS1*-FBA1p-*MsCHI*-SED1p-*Pc4CL*; XI-3::GAL10p-*SjCHS1*-FBA1p-*MsCHI*-SED1p-*Pc4CL*; XII-5::GAL10p-*SjCHS1*-FBA1p-*MsCHI*-SED1p-*Pc4CL*; XII-1::GAL10p-*SjCHS1*-FBA1p-*MsCHI*-SED1p-*Pc4CL*; ARO9::ENO2p-*ARO4^K229L^*-FBA1p-*ARO7^G141S^*; X-2::FBA1p-*ACC1^S659A, S1157A^* | | This lab |
| ZY01 | E033 | PY26-SED1p-*ApGT* | | This study |
| ZY02 | E033 | pY26-SED1p-*AtGT* | | This study |
| ZY03 | E033 | pY26-SED1p-*BvGT* | | This study |
| ZY04 | E033 | pY26-SED1p-*CjGT* | | This study |
| ZY05 | E033 | pY26-SED1p-*CsGT* | | This study |
| ZY06 | E033 | pY26-SED1p-*MdGT* | | This study |
| ZY07 | E033 | pY26-SED1p-*SlGT* | | This study |
| ZY08 | E033 | pY26-SED1p-*StGT* | | This study |
| ZY09 | E033 | pY26-SED1p-*VlGT* | | This study |
| ZY10 | E033 | pY26-TDH1p-*OlRHM*-*NRS/ER*-ADH6p-*CsGT*-SHM2p-*Cs1,6-RhaT* | | This study |
| ZY11 | E033 | pY26-TDH1p-*AtRHM1*-*NRS/ER*-ADH6p-*CsGT*-SHM2p-*Cs1,6-RhaT* | | This study |
| ZY12 | E033 | pY26-TDH1p-*VvRHM*-*NRS*-ADH6p-*CsGT*-SHM2p-*Cs1,6-RhaT* | | This study |
| ZY13 | E033 | pY26-TDH1p-*OlRHM*-*NRS/ER*-ADH6p-*AtGT*-SHM2p-*Cs1,6-RhaT* | | This study |
| ZY14 | E033 | pY26-TDH1p-*AtRHM1*-*NRS/ER*-ADH6p-*AtGT*-SHM2p-*Cs1,6-RhaT* | | This study |
| ZY15 | E033 | pY26-TDH1p-*VvRHM*-*NRS*-ADH6p-*AtGT*-SHM2p-*Cs1,6-RhaT* | | This study |
| ZY16 | E033 | pY26-TDH1p-*OlRHM*-*NRS/ER*-ADH6p-*AtGT*-SHM2p-*Ci1,6-RhaT* | | This study |
| ZY17 | E033 | pY26-TDH1p-*OlRHM1*-*NRS/ER*-ADH6p-*AtGT*-SHM2p-*Cm1,6-RhaT* | | This study |
| ZY18 | E033 | pY26-TDH1p-*OlRHM*-*NRS*-ADH6p-*AtGT*-SHM2p-*Cc1,6-RhaT* | | This study |
| ZY19 | E033 | pY26-TDH1p-*OlRHM*-*NRS*-ADH6p-*AtGT*-SHM2p-*Cc1,6-RhaT*; pRS424-TDH1p-*PGM1* | | This study |
| ZY20 | E033 | pY26-TDH1p-*OlRHM*-*NRS*-ADH6p-*AtGT*-SHM2p-*Cc1,6-RhaT*; pRS424-TDH1p-*PGM1*-INO1p-*UGP1* | | This study |
| ZY21 | E033 | pY26-TDH1p-*OlRHM*-*NRS*-ADH6p-*AtGT*-SHM2p-*Cc1,6-RhaT*; pRS424-TDH1p-*PGM2* | | This study |
| ZY22 | E033 | pY26-TDH1p-*OlRHM*-*NRS*-ADH6p-*AtGT*-SHM2p-*Cc1,6-RhaT*; pRS424-TDH1p-*PGM1*-INO1p-*PGM2* | | This study |
| ZY23 | E033 | pY26-TDH1p-*OlRHM*-*NRS*-ADH6p-*AtGT*-SHM2p-*Cc1,6-RhaT*; pRS424-TDH1p-*UGP1* | | This study |
| ZY24 | E033 | pY26-TDH1p-*OlRHM*-*NRS*-ADH6p-*AtGT*-SHM2p-*Cc1,6-RhaT*; pRS424-TDH1p-*URA6* | | This study |
| ZY25 | E033 | pY26-TDH1p-*OlRHM*-*NRS*-ADH6p-*AtGT*-SHM2p-*Cc1,6-RhaT*; pRS424-TDH1p-*YNK1* | | This study |
| ZY26 | E033 | pY26-TDH1p-*OlRHM*-*NRS*-ADH6p-*AtGT*-SHM2p-*Cc1,6-RhaT*; pRS424-TDH1p-*YNK1*-INO1p-*URA6* | | This study |
| ZY27 | E033 | pY26-TDH1p-*OlRHM*-*NRS*-ADH6p-*AtGT*-SHM2p-*Cc1,6-RhaT*; pRS424-TDH1p-*PGM2*-INO1p-*UGP1* | | This study |
| ZY28 | E033 | pY26-TDH1p-*OlRHM*-*NRS*-ADH6p-*AtGT*-SHM2p-*Cc1,6-RhaT*; pRs424-TDH1p-*URA6*-INO1p-*UGP1* | | This study |
| ZY29 | E033 | *∆ALG5*; pY26-TDH1p-*OlRHM*-*NRS*-ADH6p-*AtGT*-SHM2p-*Cc1,6-RhaT* | | This study |
| ZY30 | E033 | *∆GLC3*; pY26-TDH1p-*OlRHM*-*NRS*-ADH6p-*AtGT*-SHM2p-*Cc1,6-RhaT* | | This study |
| ZY31 | E033 | *∆FKS1*; pY26-TDH1p-*OlRHM*-*NRS*-ADH6p-*AtGT*-SHM2p-*Cc1,6-RhaT* | | This study |
| ZY32 | E033 | *∆HUT1*; pY26-TDH1p-*OlRHM*-*NRS*-ADH6p-*AtGT*-SHM2p-*Cc1,6-RhaT* | | This study |
| ZY33 | E033 | *∆YEA4*; pY26-TDH1p-*OlRHM*-*NRS*-ADH6p-*AtGT*-SHM2p-*Cc1,6-RhaT* | | This study |
| ZY34 | ZY00 | pY26-SED1p-*AtCPR*-TEF1p-*GhF3'H* | | This study |
| ZY35 | ZY00 | pY26-SED1p-*AtCPR*-TEF1p-*SmF3'H* | | This study |
| ZY36 | ZY00 | pY26-SED1p-*AtCPR*-TEF1p-*ThF3'H* | | This study |
| ZY37 | ZY00 | pY26-SED1p-*CrCPR*-TEF1p-*GhF3'H* | | This study |
| ZY38 | ZY00 | pY26-SED1p-*CrCPR*-TEF1p-*SmF3'H* | | This study |
| ZY39 | ZY00 | pY26-SED1p-*CrCPR*-TEF1p-*ThF3'H* | | This study |
| ZY40 | ZY00 | pY26-SED1p-*SmCPR*-TEF1p-*GhF3'H* | | This study |
| ZY41 | ZY00 | pY26-SED1p-*SmCPR*-TEF1p-*SmF3'H* | | This study |
| ZY42 | ZY00 | pY26-SED1p-*SmCPR*-TEF1p-*ThF3'H* | | This study |
| ZY43 | ZY00 | pY26-SED1p-*AtCPR*-INO1p-*ThF3'H* | | This study |
| ZY44 | ZY00 | pY26-SED1p-*AtCPR*-TDH1p-*ThF3'H* | | This study |
| ZY45 | ZY00 | pY26-INO1p-*AtCPR*-TDH1p-*ThF3'H* | | This study |
| ZY46 | ZY00 | pY26-INO1p-*AtCPR*-TEF1p-*ThF3'H* | | This study |
| ZY47 | ZY00 | pY26-INO1p-*AtCPR*-SED1p-*ThF3'H* | | This study |
| ZY48 | ZY00 | pY26-TDH1p-*AtCPR*-SED1p-*ThF3'H* | | This study |
| ZY49 | ZY00 | pY26-TDH1p-*AtCPR*-TEF1p-*ThF3'H* | | This study |
| ZY50 | ZY00 | pY26-TDH1p-*AtCPR*-INO1p-*ThF3'H* | | This study |
| ZY51 | ZY00 | pY26-TEF1p-*AtCPR*-INO1p-*ThF3'H* | | This study |
| ZY52 | ZY00 | pY26-TEF1p-*AtCPR*-SED1p-*ThF3'H* | | This study |
| ZY53 | ZY00 | pY26-TEF1p-*AtCPR*-TDH1p-*ThF3'H* | | This study |
| ZY54 | ZY00 | XII-2::TEF1p-*AtCPR*-TDH1p-*ThF3'H* | | This study |
| ZY55 | ZY54 | XI-1::TEF1p-*AtCPR*-TDH1p-*ThF3'H* | | This study |
| ZY56 | ZY55 | XII-3::TEF1p-*AtCPR*-TDH1p-*ThF3'H* | | This study |
| ZY57 | ZY56 | X-4::TEF1p-*AtCPR*-TDH1p-*ThF3'H* | | This study |
| ZY58 | ZY57 | pRS423-TDH1p-*AtCPR*-SED1p-*ThF3'H* | | This study |
| ZY59 | ZY00 | *∆EXG1* | | This study |
| ZY60 | ZY00 | *∆SPR1* | | This study |
| ZY61 | ZY00 | *∆EGH1* | | This study |
| ZY62 | ZY00 | *∆SCW2* | | This study |
| ZY63 | ZY00 | *∆SIM1* | | This study |
| ZY64 | ZY00 | *∆EXG1*; *∆SPR1* | | This study |
| ZY65 | ZY00 | *∆EXG1*; *∆EGH1* | | This study |
| ZY66 | ZY00 | *∆SPR1*; *∆EGH1* | | This study |
| ZY67 | ZY00 | *∆EXG1*; *∆SPR1*; *∆EGH1* | | This study |
| ZY68 | ZY58 | *∆EXG1*; *∆SPR1*; *∆EGH1*; pY26-TDH1p-*OlRHM*-*NRS*-ADH6p-*AtGT*-SHM2p-*Cc1,6-RhaT* | | This study |
| ZY69 | ZY68 | pRS424-TDH1p-*PGM2*-INO1p-*UGP1* | | This study |
| ZY70 | ZY69 | *∆ALG5*; | | This study |
| ZY71  ZY72 | ZY68  ZY68 | *∆ALG5*; *∆GLC3*; pRS424-TDH1p-*YNK1*-INO1p-*URA6*  *∆ALG5*; *∆GLC3*; pRS424-TDH1p-*URA6*-INO1p-*UGP1* | | This study  This study |

### Table S7

**Table S7.** Characterization of UF7GT genes through bioinformatics analysis.

| Original organisms | Accession | ORF (bp) | Number of amino acids (aa) | Molecular weight (kDa) | Theoretical pI | Instability index | Aliphatic inde |
| --- | --- | --- | --- | --- | --- | --- | --- |
| *Andrographis paniculata* | MH379335 | 1365 | 454 | 49.25 | 6.04 | 47.97 | 89.69 |
| *Arabidopsis thaliana* | AY090273 | 1467 | 488 | 54.84 | 5.51 | 36.41 | 85.66 |
| *Citrus japonica* | MN393519 | 1497 | 498 | 55.94 | 5.11 | 44.75 | 93.94 |
| *Camellia sinensis* | KP682364 | 1419 | 472 | 52.45 | 5.84 | 51.92 | 87.97 |
| *Glycyrrhiza uralensis* | KT759000 | 1466 | 481 | 53.87 | 5.88 | 49.69 | 83.28 |
| *Nicotiana tabacum* | U32644 | 1431 | 476 | 53.61 | 5.76 | 43.07 | 81.09 |
| *Scutellaria laeteviolacea* | AB362989 | 1368 | 455 | 50.16 | 5.38 | 35.77 | 94.7 |
| *Solanum lycopersicum* | NM_001246931 | 1449 | 482 | 54.23 | 5.8 | 42.27 | 94.07 |
| *Solanum tuberosum* | MF134427 | 1518 | 505 | 57.05 | 5.55 | 42.76 | 91.52 |
| *Vitis labrusca* | EF533705 | 1344 | 447 | 49.71 | 5.41 | 44.3 | 90.92 |
| *Beta vulgaris* | AY526081 | 1786 | 492 | 54.4 | 5.5 | 47.68 | 92.7 |
| *Malus domestica* | AY786997 | 1446 | 481 | 53.49 | 4.94 | 39.58 | 89.75 |
| *Pyrus communis* | AY954922 | 1446 | 481 | 53.26 | 5.01 | 36.12 | 91.56 |
| *Dorotheanthus bellidiformis* | Y18871 | 1470 | 489 | 55.24 | 5.95 | 42.35 | 80.02 |
| *Scutellaria baicalensis* | AB031274 | 1431 | 476 | 53.1 | 5.57 | 40.44 | 77.44 |
| *Paeonia delavayi* | KX394687 | 1446 | 481 | 54.45 | 6.1 | 39.58 | 89 |
| *Glycine max* | NM_001317558 | 1422 | 473 | 52.03 | 5.36 | 44.13 | 95.48 |
| *Marchantia emarginata* | MW392738 | 1479 | 492 | 55.58 | 5.85 | 47.18 | 88.6 |
| *Withania somnifera* | FJ560880 | 1371 | 456 | 51.42 | 5.32 | 48.2 | 93.6 |
| *Albuca bracteata* | MF621962 | 1440 | 479 | 53.17 | 5.29 | 41.15 | 90 |
| *Torenia hybrid* | AB477351 | 1386 | 461 | 50.82 | 6.35 | 40.29 | 91.56 |
| *Antirrhinum majus* | AB371298 | 1488 | 495 | 55.32 | 5.6 | 42.09 | 92.69 |
| *Tripterygium wilfordii* | MK035745 | 1428 | 475 | 52.68 | 6.25 | 38.65 | 93.16 |
| *Hieracium pilosella* | EU561019 | 1404 | 467 | 52.36 | 5.53 | 27.77 | 88.91 |
| *Ziziphus jujuba var. spinosa* | OQ603459 | 1440 | 479 | 53.55 | 5.49 | 47.55 | 89.52 |
| *Perilla frutescens* | AB362992 | 1419 | 472 | 51.89 | 5.29 | 42.82 | 97.73 |
| *Rubus hybrid cultivar* | KM061379 | 1380 | 459 | 50.75 | 5.79 | 43.66 | 92.14 |
| *Scutellaria barbata* | GU339042 | 1434 | 477 | 52.85 | 5.47 | 47.27 | 83.82 |
| *Allium cepa* | AY262062 | 1440 | 479 | 53.58 | 6.07 | 42.71 | 93.01 |
| *Dianthus caryophyllus* | AB191248 | 1428 | 475 | 53.62 | 5.59 | 41.54 | 79.49 |

### Table S8

**Table S8.** REME platform forecast information for UF7GTs genes.

| Original organisms | Accession | Km Mol/L （KM_prediction） | | Kcat 1/s（TurNuP） | Kcat 1/s（DLKcat） | | | Optimum  temperature（DeepET） | optimum pH（epHod） | | |
| --- | --- | --- | --- | --- | --- | --- | --- | --- | --- | --- | --- |
| *Andrographis paniculata* | MH379335 | 0.04 | 21.9 | | | 20.71 | 66.9 | | | 8 |  |
| *Arabidopsis thaliana* | AY090273 | 0.27 | 19.86 | | | 9.14 | 56.3 | | | 7.9 |  |
| *Citrus japonica* | MN393519 | 0.31 | 28.14 | | | 12.21 | 56.2 | | | 8.08 |  |
| *Camellia sinensis* | KP682364 | 0.26 | 35.42 | | | 14.08 | 57.3 | | | 7.95 |  |
| *Glycyrrhiza uralensis* | KT759000 | 0.3 | 28.39 | | | 14.41 | 55.2 | | | 8.05 |  |
| *Nicotiana tabacum* | U32644 | 0.24 | 16.67 | | | 23.63 | 56.7 | | | 8.23 |  |
| *Scutellaria laeteviolacea* | AB362989 | 0.06 | 15.04 | | | 100.51 | 63.8 | | | 7.87 |  |
| *Solanum lycopersicum* | NM_001246931 | 0.19 | 35.35 | | | 37.03 | 57.6 | | | 8.16 |  |
| *Solanum tuberosum* | MF134427 | 0.22 | 36.03 | | | 22.6 | 58.9 | | | 8.19 |  |
| *Vitis labrusca* | EF533705 | 0.1 | 23.9 | | | 29.25 | 59.6 | | | 7.98 |  |
| *Beta vulgaris* | AY526081 | 0.1 | 31.38 | | | 9.87 | 56 | | | 8.01 |  |
| *Malus domestica* | AY786997 | 0.34 | 18.72 | | | 2.77 | 56.7 | | | 7.98 |  |
| *Pyrus communis* | AY954922 | 0.33 | 22.37 | | | 3.07 | 58.9 | | | 7.99 |  |
| *Dorotheanthus bellidiformis* | Y18871 | 0.11 | 25.2 | | | 13.13 | 56.3 | | | 8.15 |  |
| *Scutellaria baicalensis* | AB031274 | 0.09 | 22.37 | | | 34.59 | 55 | | | 8.16 |  |
| *Paeonia delavayi* | KX394687 | 0.28 | 21.86 | | | 6.17 | 55.9 | | | 8.02 |  |
| *Glycine max* | NM_001317558 | 0.31 | 20.25 | | | 17.39 | 55.2 | | | 8.21 |  |
| *Marchantia emarginata* | MW392738 | 0.11 | 24.16 | | | 69.03 | 56.9 | | | 8.04 |  |
| *Withania somnifera* | FJ560880 | 0.11 | 29.57 | | | 14.51 | 57 | | | 8.07 |  |
| *Albuca bracteata* | MF621962 | 0.37 | 19.04 | | | 7.27 | 55 | | | 8.12 |  |
| *Torenia hybrid* | AB477351 | 0.05 | 24.21 | | | 38.01 | 59.7 | | | 8.05 |  |
| *Antirrhinum majus* | AB371298 | 0.22 | 19.86 | | | 6.07 | 60.8 | | | 7.89 |  |
| *Tripterygium wilfordii* | MK035745 | 0.11 | 22.76 | | | 20.67 | 54.7 | | | 8.05 |  |
| *Hieracium pilosella* | EU561019 | 0.27 | 23.75 | | | 41.72 | 55.7 | | | 8.01 |  |
| *Ziziphus jujuba var. spinosa* | OQ603459 | 0.1 | 22.56 | | | 12.1 | 55.9 | | | 8 |  |
| *Perilla frutescens* | AB362992 | 0.07 | 16.03 | | | 15.02 | 57.4 | | | 8.13 |  |
| *Rubus hybrid cultivar* | KM061379 | 0.31 | 20.46 | | | 27.68 | 58 | | | 8.16 |  |
| *Scutellaria barbata* | GU339042 | 0.25 | 21.86 | | | 6.97 | 55.6 | | | 8.17 |  |
| *Allium cepa* | AY262062 | 0.1 | 30.11 | | | 5.34 | 55.2 | | | 7.97 |  |
| *Dianthus caryophyllus* | AB191248 | 0.25 | 23.75 | | | 6.73 | 55.2 | | | 8.05 |  |

### Figure S1


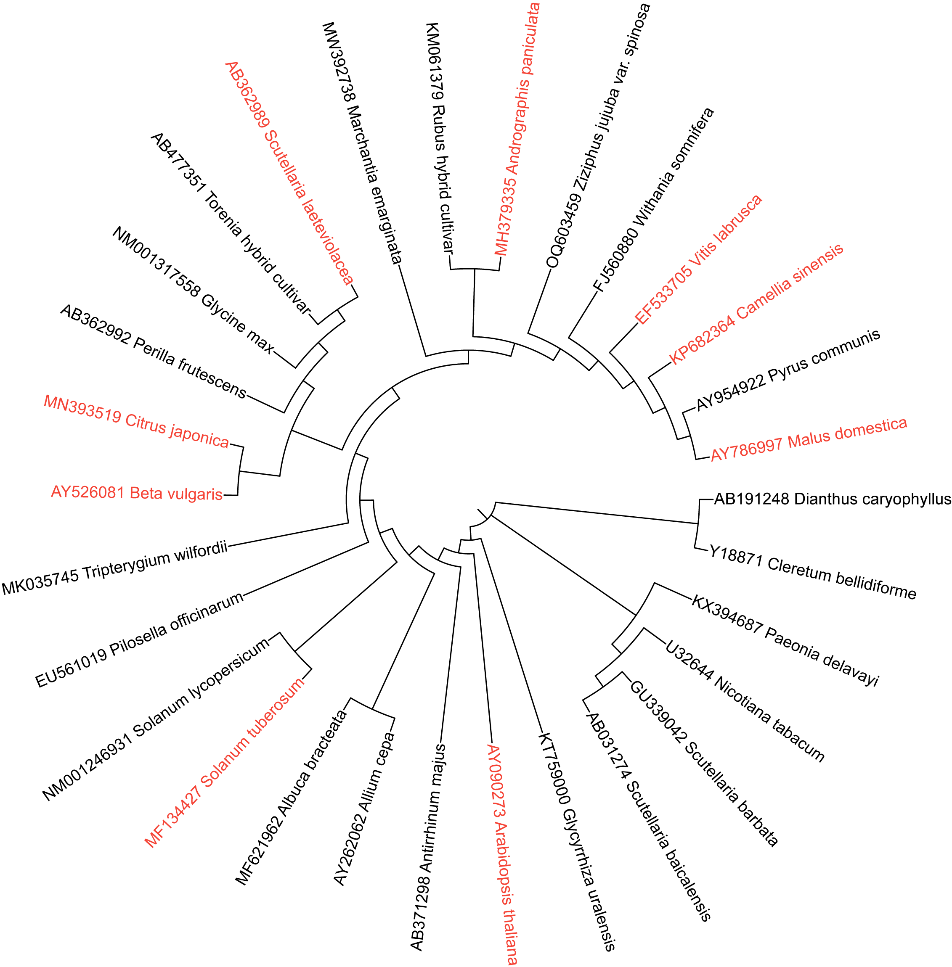


**Figure S1.** Phylogenetic tree of UF7GTs. the red identifier indicates UF7GT used in this experiment.

### Figure S2


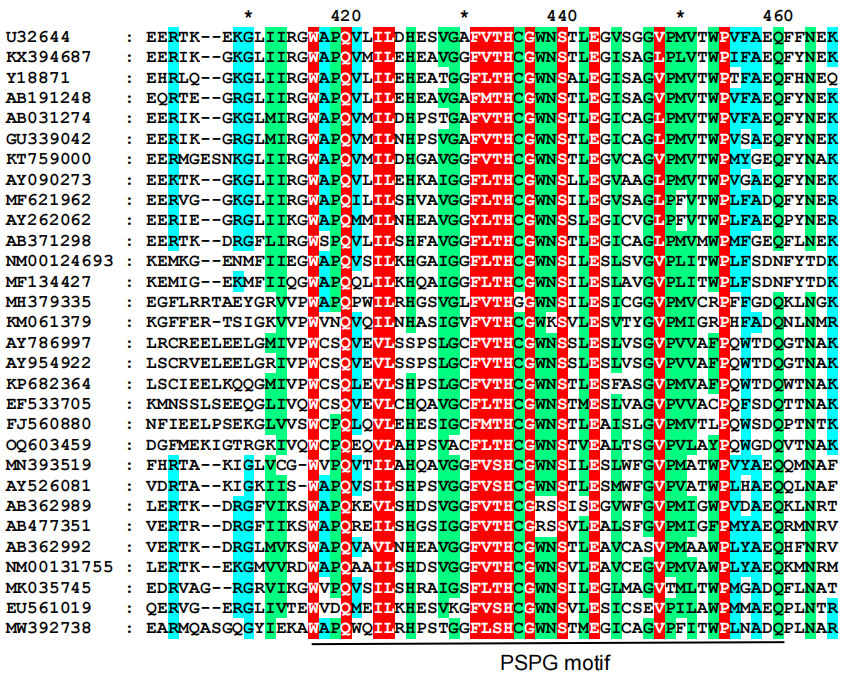


**Figure S2.** Multiple sequence alignment of the PSPG motifs between the thirty flavonoid glucosyltransferases, with identical and similar residues highlighted.

### Figure S3


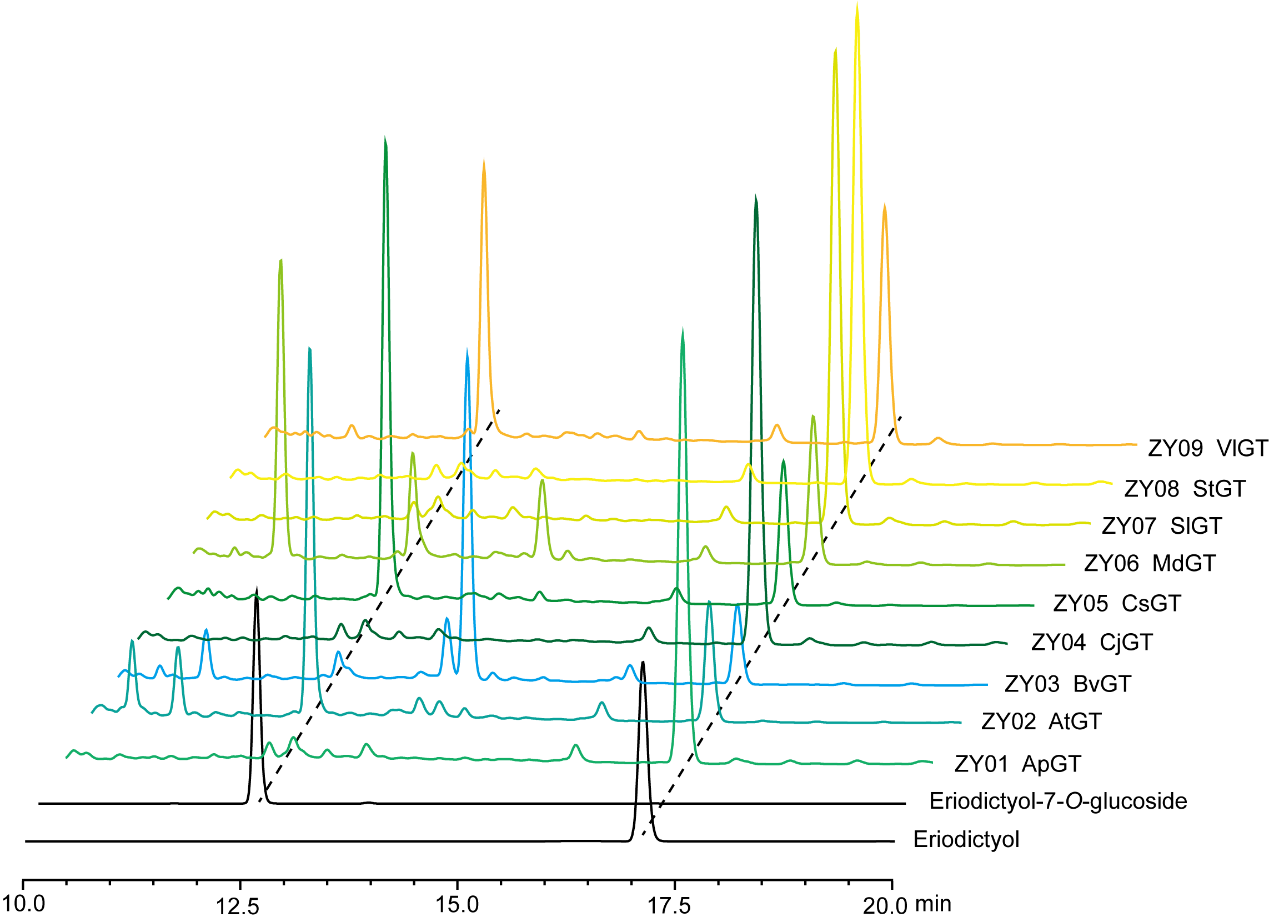


**Figure S3.** HPLC profiles of nine UF7GTs.

### Figure S4


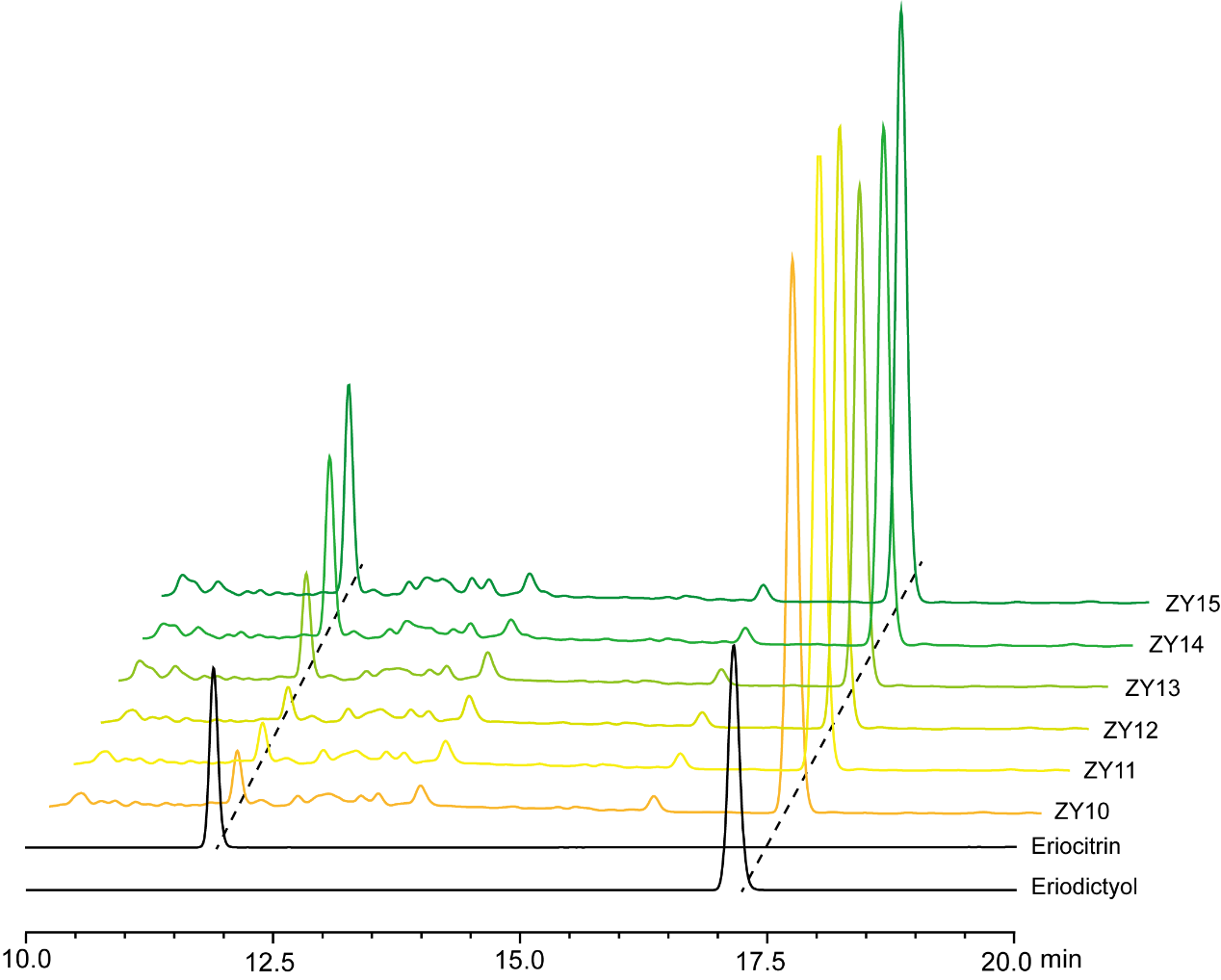


**Figure S4.** HPLC profiles of strain ZY10-ZY15.

### Figure S5


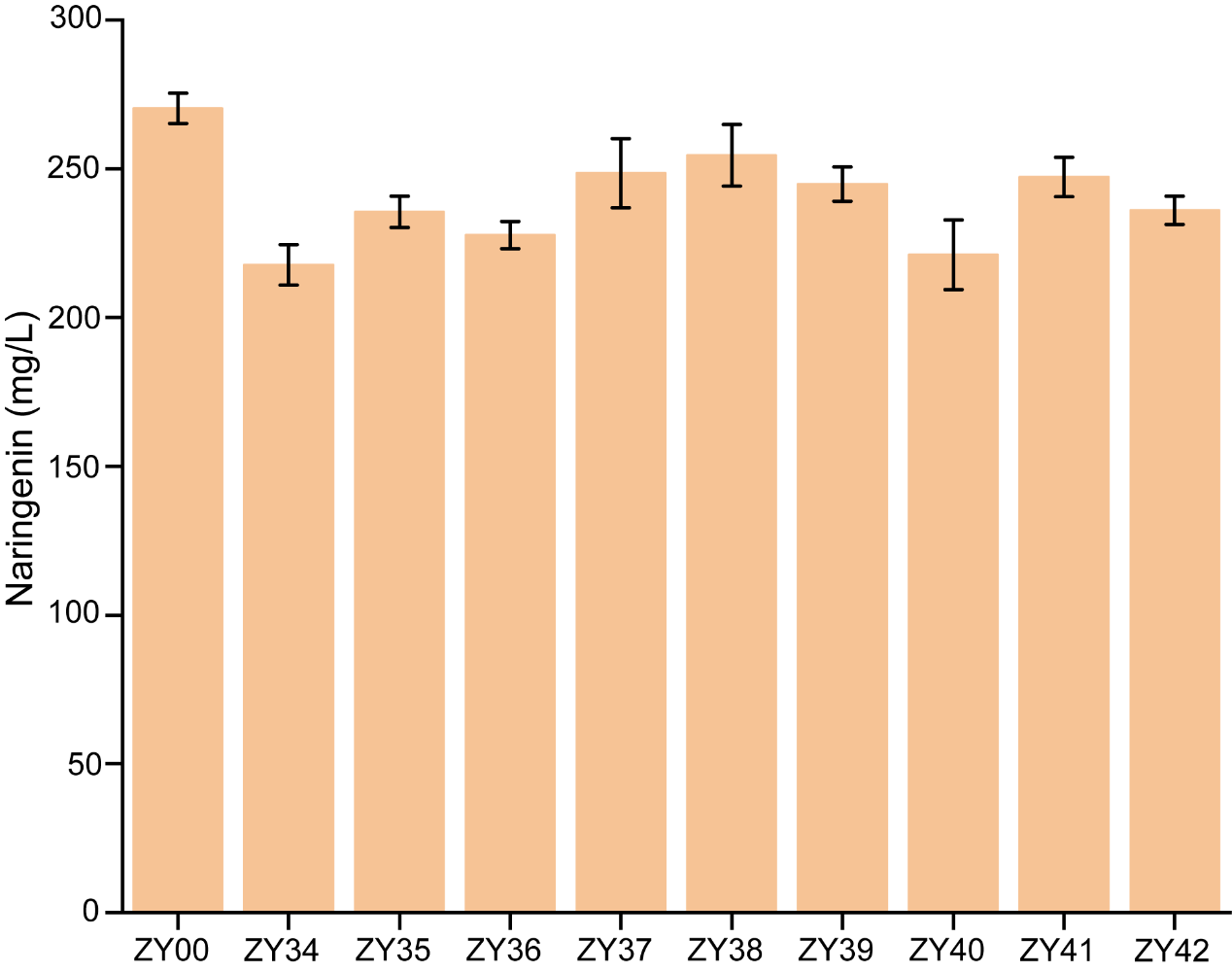


**Figure S5.** Quantification of residual naringenin in strains ZY34-ZY42 after fermentation.

### Figure S6


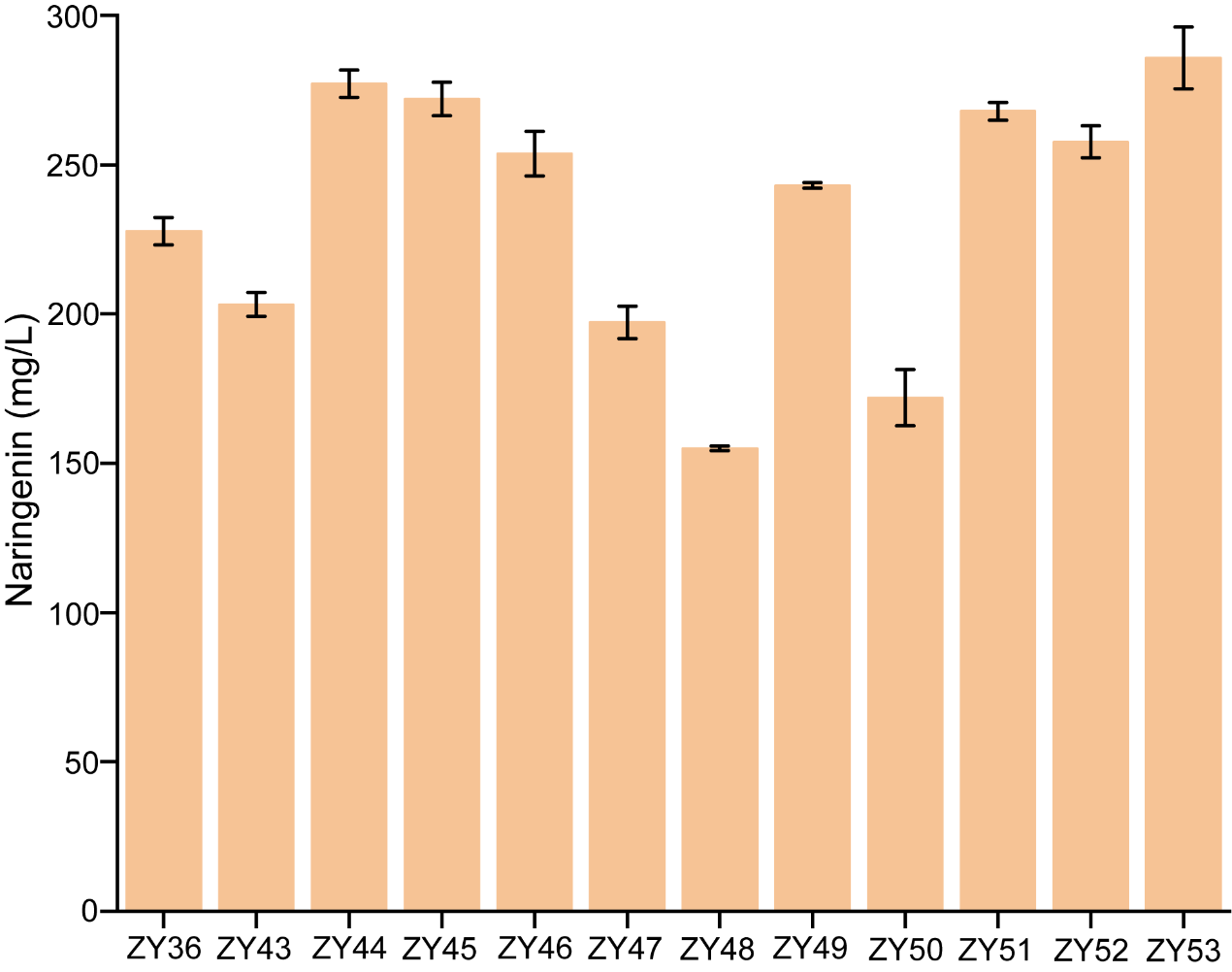


**Figure S6.** Quantification of residual naringenin in strains ZY43-ZY53 after fermentation.

### References

[1] S. Gao, X. Xu, W. Zeng, S. Xu, Y. Lyv, Y. Feng, G. Kai, J. Zhou, J. Chen. Efficient biosynthesis of (*2S*)-eriodictyol from (*2S*)-naringenin in *Saccharomyces cerevisiae* through a combination of promoter adjustment and directed evolution. ACS Synth Biol 2020;9:3288-3297. <https://doi.org/10.1021/acssynbio.0c00346>
